# Implications of the Cultivation of Rosemary and Thyme (*Lamiaceae*) in Plant Communities for the Development of Antioxidant Therapies

**DOI:** 10.3390/ijms241411670

**Published:** 2023-07-19

**Authors:** Emanuela-Alice Luță, Andrei Biță, Alina Moroșan, Dan Eduard Mihaiescu, Dragoș Paul Mihai, Liliana Popescu, Ludovic Everard Bejenaru, Cornelia Bejenaru, Violeta Popovici, Octavian Tudorel Olaru, Cerasela Elena Gîrd

**Affiliations:** 1 Faculty of Pharmacy, University of Medicine and Pharmacy “Carol Davila”, Traian Vuia 6, 020956 Bucharest, Romania; emanuela.luta@drd.umfcd.ro (E.-A.L.); octavian.olaru@umfcd.ro (O.T.O.); cerasela.gird@umfcd.ro (C.E.G.); 2 Department of Pharmacognosy & Phytotherapy, Faculty of Pharmacy, University of Medicine and Pharmacy of Craiova, Petru Rareș 2, 200349 Craiova, Romania; andrei.bita@umfcv.ro (A.B.); ludovic.bejenaru@umfcv.ro (L.E.B.); 3 Department of Organic Chemistry “Costin Nenițescu”, Faculty of Chemical Engineering and Biotechnologies, University of Politehnica, Gheorghe Polizu 1–7, 011061 Bucharest, Romania; alina.morosan@upb.ro (A.M.); dan.mihaiescu@upb.ro (D.E.M.); 4 Department of Pharmaceutical Botany, Faculty of Pharmacy, University of Medicine and Pharmacy of Craiova, Petru Rareș 2, 200349 Craiova, Romania; cornelia.bejenaru@umfcv.ro; 5 Department of Microbiology and Immunology, Faculty of Dental Medicine, Ovidius University of Constanta, 7 Ilarie Voronca Street, 900684 Constanta, Romania; popovicivioleta@gmail.com

**Keywords:** *Rosmarinus officinalis* L., *Thymus vulgaris* L., phytosociology, medicinal plants, dry extracts, polyphenols, antioxidant activity, molecular docking, BACH1/BACH2

## Abstract

Oxidative stress is the most critical factor in multiple functional disorders’ development, and natural antioxidants could protect the human body against it. Our study aims to investigate the polyphenol content of four extracts of two medicinal plants (*Rosmarinus officinalis* L. and *Thymus vulgaris* L.) and analyze the correlation with their antioxidant activity. The research was carried out on extracts of rosemary and thyme obtained from species cultivated together in plant communities. Both were compared with extracts from species cultivated in individual crops (control crops). Their polyphenols were determined by spectrophotometric methods (dosage of flavones, phenol carboxylic acids, and total polyphenols) and chromatography (UHPLC–MS and FT–ICR MS). Triterpenic acids were also quantified, having a higher concentration in the thyme extract from the culture. The antioxidant activity of the dry extracts was evaluated in vitro (DPPH, ABTS, and FRAP) and in silico (prediction of interactions with BACH1/BACH2 transcription factors). The concentrations of polyphenols are higher in the extracts obtained from the sources collected from the common crops. These observations were also validated following the chromatographic analysis for some compounds. Statistically significant differences in the increase in the antioxidant effect were observed for the extracts from the common batches compared to those from the individual ones. Following the Pearson analysis, the IC_50_ values for each plant extract were strongly correlated with the concentration of active phytoconstituents. Molecular docking studies revealed that quercetin could bind to BTB domains of BACH1 and BACH2 transcription factors, likely translating into increased antioxidant enzyme expression. Future studies must validate the in silico findings and further investigate phytosociological cultivation’s effects.

## 1. Introduction

Advanced research in medicinal plants is essential for phytotherapy. Identifying and characterizing new secondary metabolites create up-to-date therapeutic premises in alleviating to total abolition of some intense symptomatologies—which are difficult to manage sometimes—even with the help of organic-based drugs. In this context, identifying new methodologies for cultivating medicinal plants that would generate higher quantities of active ingredients may concern many manufacturers of supplements/phytomedicines.

Therefore, phytosociology, as a branch of the science of vegetation that deals with plant communities, can be a starting point in research that aims to exalt plant raw materials into active chemical constituents due to increasing their biosynthesis capacity. Sampling strategies usually cover the full vegetation variability within a study’s defined geographical and ecological boundaries while minimizing within-plot heterogeneity. According to modern empirical and theoretical knowledge, the average richness of species increases monotonically with the area, and a minimum region is a delusion caused by the non-linear nature of species–area relationships [1].

Current phytosociology [2,3] is a statistical approach that aims to characterize different vegetation types through combined information from several other plots [4,5]. Thus, some medicinal species from the same family, different genera, or different families (association of plant species) cultivated together (common crops) could generate higher amounts of secondary metabolites with antioxidant activity, substantially benefiting people’s health [6,7]. The incrimination of reactive oxygen species (ROS) in numerous pathologies is well known [8]. Practically, we can say that there is no disease in which these small and highly reactive molecular fractions are absent. ROS can be produced by endogenous sources (mitochondria, cytochrome P450′s metabolism, peroxisomes, and inflammatory cell activation) and exogenous ones (involvement of numerous cofactors from various conditions, drug administration, chemicals, and atmospheric pollutants) [9,10]. Natural antioxidants (from foods or standardized plant extracts) could have a protective role at the cellular level [11]. Most of them are phenolic compounds with a very well-known antioxidant potential [12]. Phenolic compounds can exert antioxidant effects by directly neutralizing free radicals or interacting with signaling pathways involved in cellular redox homeostasis. This implies transcription regulator proteins expressed in various mammalian tissues (transcription factors BTB and CNC homolog 1—BACH1 and BTB and CNC homolog 2—BACH2). BACH1 suppresses the activity of transcription factor nuclear factor erythroid 2-related factor 2 (Nrf2) by binding to antioxidant response element (ARE) promoter elements. It diminishes the expression of endogenous antioxidant enzymes (heme oxygenase-1) [13]. BACH2 competes with Nrf2 for AREs, reducing the antioxidant defense [14]. Therefore, inhibition of BACH1 and BACH2 transcription factors can stimulate the endogenous antioxidant defense in various diseases by derepressing the activity of Nrf2.

Numerous studies from the accessed scientific literature underline the pharmacological potential and phytotherapeutic applications of *Rosmarinus officinalis* L. [15,16,17,18,19] and *Thymus vulgaris* L. [20,21,22,23,24] from the *Lamiaceae* family. Rosemary leaves contain 1–2.5% volatile oil with a variable composition, depending on the crop variety and pedoclimatic conditions. The major constituents are 1–8 cineol (20–50%), alpha-pinene (15–26%), camphor (10–25%), alpha-terpineol (12–24%), camphene (5–10%), borneol (1–6%), and bornyl acetate (1–5%) [25]. Limonene, beta-pinene, beta-caryophyllene, myrcene, polyphenolcarboxylic acids (rosmarinic, caffeic, chlorogenic, and neochlorogenic acid), bitter substances with tricyclic diterpenoid structure (carnosol = picrosalvin, carnosolic acid, rosmanol, rosmadial, rosmaridiphenol), pentacyclic triterpenes (ursolic acid, oleanolic acid, alpha-amirenol, beta-amirenol), and flavonic derivatives (apigenol, luteolin, diosmetin, diosmin, hispidulin, nepetin) [26,27] are identified in small quantities. In vitro and in vivo studies reported that rosemary extracts have spasmolytic action (on the biliary tract and small intestine), inotropic positive, coronary dilator, and diuretic effects, and hepatoprotective, antimicrobial, antiviral, and anti-inflammatory activity [28,29,30,31]. When these extracts are administered externally, activation of blood circulation was also observed due to the revulsive effect [32]. The *Thymus vulgaris* L. (thyme) aerial parts contain 1.5–2% volatile oil. Thyme essential oil is rich in thymol and carvacrol (60%), pinene, terpinene, flavonoids, tannins, triterpenic acids (oleanolic, ursolic), phenolic acids (quinic and caffeic acid), and vitamins (vitamin C and vitamin B complex) [33,34]. The therapeutic effects are due to the phytocomplex; thus, it has antibacterial action through flavonoids and volatile oil, cicatrizing action through tannin and terpenoids, and antioxidant action through tannin and flavonoids [35]. Beyond everyday culinary use, thyme is recommended for many ailments of the respiratory system (cough and upper respiratory congestion, bronchitis) and digestive tube [36].

The main scope of the proposed study was to obtain enriched dry extracts with superior antioxidant activity from two medicinal plants, rosemary and thyme, through cultivation in plant communities, for their potential use in oxidative stress-related disorders. In this regard, the positive impact on antioxidant activity was investigated in a phytosociology study. The dry extracts from both species harvested from phytosociological and control crops were analyzed to evaluate their phenolic content and antioxidant activity. The polyphenolic derivatives and triterpenic acids were quantified by spectrophotometry and chromatography (UHPLC–MS and HR ESI–MS). The antioxidant activity was evaluated using in vitro (analyzing the direct antioxidant activity with free radical-scavenging tests) and in silico (assessing the potential of identified phytochemicals to inhibit BACH1/BACH2 activities) methods. Moreover, extensive statistical analyses were performed, aiming to support our results.

## 2. Results

### 2.1. Determination of Polyphenolic Compounds Using Spectrophotometric Methods

The results obtained from the quantitative analysis of the active phytoconstituents are presented in Table 1. We note that the dry extracts obtained from the four types of plant raw materials were powdery and homogeneous, showing a specific color and smell.

Based on the analysis of the obtained results, it is found that there is a difference between the content of active phytoconstituents determined in the control samples compared to those from the crops where the medicinal species were cultivated in phytosociological ones. Thus, for REF, the flavone content (78.323 ± 12.47) is 1.32 times higher compared to REM (59.061 ± 4.01), TPCAs are 1.27 times higher, and TPC is 1.38 times higher. TEM, one of the control groups, has a content of flavones (72.094 ± 18.04) that is 0.73 times lower compared to TEF (98.203 ± 13.65), a content of TPCAs 0.82 times lower, and a content of TPC (275.507 ± 22.56) 0.85 times lower.

### 2.2. Identification and Quantification of Polyphenolic Compounds Using Ultra-High Performance Liquid Chromatography–MS (UHPLC–MS)

The chromatographic analysis results are displayed in Table 2. The representative chromatograms for each type of extract are shown in Supplementary Materials Figure S8.

Most polyphenolic compounds were identified in all analyzed extracts compared to the standards. Unquantified rutoside and quercetin in the rosemary extract obtained from the control crop represented exceptions. Ferulic acid was not detected in any sample (Table 2).

### 2.3. Identification of Polyphenolic Compounds Using FT–ICR MS

The results suitable for identifying polyphenolic derivatives for ESI+ and ESI− are presented in Table 3, and all mass spectrum data for identified compounds can be found in the Supplementary Materials, Figures S9–S46.

Being a slightly different analysis, FT–ICR MS also uses positive ionization; some compounds can be identified and others not. Thus, the identification of *p*-coumaric acid was different for rosemary extract, which ionizes in the negative. This method highlighted rutoside in ESI+ in both rosemary samples. Moreover, ferulic acid was identified in REM and REF for both ESI+ and ESI− compared to the UHPLC method. It could also be found in thyme extracts in ESI−.

### 2.4. Identification of Triterpenic Acids by UHPLC–MS and FT–ICR MS (ESI+ and ESI−)

**Figure 1:**
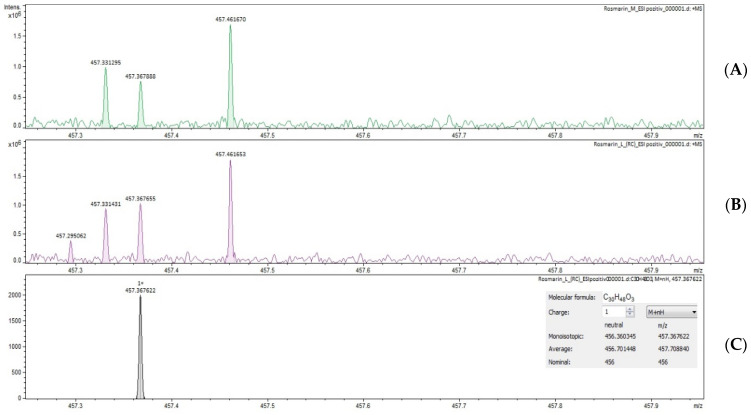
Mass spectra of triterpenic acids, ESI+ ionization; (**A**) REM, (**B**) REF, (**C**) theoretical pick; REM—rosemary extract, control lot; REF—rosemary extract, phytosociological crop.

**Figure 2:**
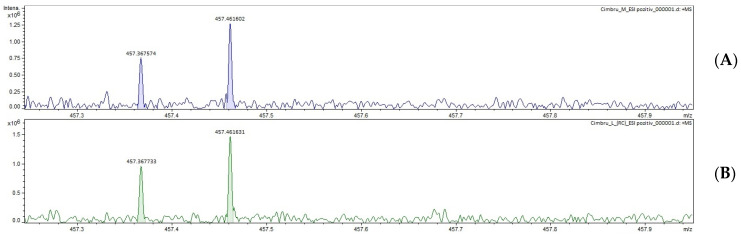

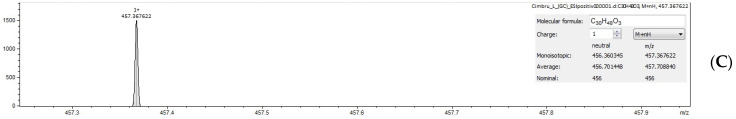
Mass spectra of triterpenic acids, ESI+ ionization; (**A**) TEM, (**B**) TEF, (**C**) theoretical pick; TEM—thyme extract, control lot; TEF—thyme extract, phytosociological crop.

The results are presented in Table 4 and the mass spectra in Figure 1 and Figure 2.

Triterpenic acids have a heterogeneous distribution in the analyzed extracts (Table 4). The content of oleanolic and ursolic acids is higher in the extracts obtained from the control crops. Therefore, the oleanolic acid content is 1.34 times higher than the control in rosemary extract obtained from the common crop.

Through the FT–ICR MS method, triterpenic acids could be identified only as isomers with the same molecular formula. Both rosemary and thyme extracts could only be determined by positive ionization.

### 2.5. In Vitro Evaluation of Antioxidant Activity

Through the three methods (DPPH, ABTS, and FRAP), the antioxidant effect of the common crops was analyzed compared to controls. These antioxidant assays highlighted the significant differences between the extracts from the common cultures and those obtained from the control ones. They could demonstrate an improved antioxidant effect for cultures grown together than individual ones.

Until now, no studies have evaluated phytosociological crops’ phytochemical composition and antioxidant activity. The present research would provide a much better perspective to manufacturers on obtaining ennobled plant extracts in phytosociological cultures.

The antioxidant effect of each experimental batch cultivated in phytosociological conditions related to the investigated plant extracts (REF, TEF) was compared with the antioxidant activity by the control crop (REM, TEM) that was cultivated alone without the influence of other crops (Figure S47).

#### 2.5.1. DPPH Free Radical-Scavenging Activity

Two variables (∆_ROS_DPPH and ∆_THYM_DPPH) were included, quantifying the antiradical effect. They were the difference between DPPH free radical scavenging of the common group and control for rosemary and thyme. The obtained data are displayed in Table 5.

#### 2.5.2. ABTS Method

The differences between inhibition of the free radical ABTS in the phytosociological groups compared to the control groups were statistically evaluated. The data obtained are registered in Table 6.

The ABTS database also includes two variables (∆_ROS_ABTS and ∆_THYM_ABTS). They are expressed as the difference between the ABTS free radical inhibition of the common group and that of the control for the rosemary and thyme extracts.

#### 2.5.3. FRAP Assay

The FRAP technique evaluated the antioxidant activity in terms of optical density, and the results are displayed in Table 7. Similar variables (∆_ROS_FRAP and ∆_THYM_FRAP) to the previous ones were included. A higher optical density corresponds to plant extracts’ more robust antioxidant activity.

### 2.6. Statistical Analysis

#### 2.6.1. Descriptive Analysis and Comparison of Antioxidant Activity between Common and Control Crops

The evaluation of the results was followed by quantifying the antioxidant potency of the extracts and finding if the statistical analysis has significant power. The descriptive statistics are illustrated in Table S3 (Supplementary Materials). Applying parametric tests, we evaluated the statistically significant differences between the increased scavenging activity of the common crop extracts compared to the controls.

The descriptive analysis was carried out on the inhibition and optical density results obtained using the three antioxidant activity evaluations (DPPH, ABTS, and FRAP).

If we look at Table S3 from the Supplementary Materials, an inhibition of the DPPH free radical-scavenging activity (45.4704%) was observed in the control group of rosemary grown alone. The inhibition (57.8046%) increases in the rosemary from the common group. Therefore, an increase in the inhibition of 12.3342% was observed in the phytosociological group compared to the control group in the case of rosemary extracts. In the case of thyme, the average inhibition was 41.7573% for the control group and 48.9385% for the phytosociological group, with the antioxidant effect improving on average by 7.1812%.

We also see an appreciable difference between the standard deviations (SDs) in the last columns (∆_ROS_DPPH vs. ∆_THYM_DPPH: 6.3346% vs. 3.6361%).

For the DPPH method, all data sets passed the test of Shapiro–Wilk normality, which shows that they have a Gaussian distribution (for this reason, we applied parametric statistical tests to evaluate the differences that appear).

In the independent samples *t*-Test (Table 8), we obtained a significant difference between the phytosociological groups, revealed by the two-tailed *p*-value (*p* = 0.039; t = 2.231), considered highly significant (*p* < 0.05).

Therefore, there is a statistically significant difference between the antioxidant effects (DPPH method) of the phytosociological groups grown together (rosemary–thyme) compared to the control groups (*p* = 0.039; *p* < 0.05).

The mean difference is reported in Table 8 as the mean of ∆_ROS_DPPH minus the mean of ∆_THYM_DPPH (mean difference = 5.1530%), with a 95% confidence interval of 0.3004 to 10.0055.

To quantify the size of the antioxidant effect and identify how much one group differs from another—usually a difference between an experimental group compared with a control group—Hedges’ g index was evaluated. Hedges’ g is a measure of effect size (ES = effect size) that is evidenced by testing. A Hedges’ g index of approximately 1 (Hedges’ g _DPPH_ = 0.9555) indicates that the two groups differ by 1 standard deviation (standard deviations are equivalent to z-scores: 1 standard deviation = 1 z-score).

In our study, we report the Hedges’ g because we cannot access the population standard deviation; we only have access to the sample standard deviations (Table 9).

According to the descriptive statistics of the ABTS data set (Table S4), the phytosociological groups exerted grater average inhibition on the reactive species for the ABTS method than the control groups, with rosemary’s antioxidant effect increasing by 12.4696% and thyme’s by 7.4322%.

The data from the last two columns (∆_ROS_ABTS and ∆_THYM_ABTS) did not comply with all the normality assumptions. However, they passed the test of Shapiro–Wilk normality (*p* > 0.05), registering a distortion or asymmetry that deviates from the symmetrical bell curve. As the distribution curve is shifted (highly positively or negatively skewed) and the scientific data are resistant to transformation, we applied non-parametric statistical tests because these tests do not have normality limitations.

**Figure 3:**
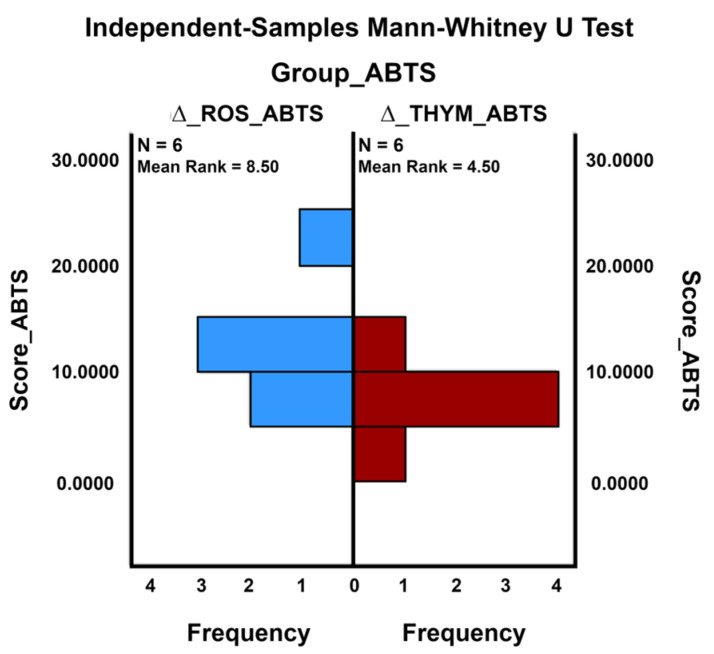
Comparison of ABTS antioxidant effect with Mann–Whitney U test.

When certain distribution assumptions are unmet, the Mann–Whitney U test was used as an alternative non-parametric statistical test to the standard parametric independent samples *t*-Test. Thus, the Mann–Whitney U test compared the analyzed antioxidant groups (rosemary and thyme) concerning the mean ranks of the antioxidant effect measured in each set (Figure 3).

A Mann–Whitney U test was performed to evaluate the differences between the antioxidant activity of the rosemary and thyme dry extracts from the same phytosociological crop, considering the control group’s results. Then, running the analysis of two-group data with a quantitative response variable (the inhibition of the activity of the ABTS free radical), we examined the distributions of scores on a quantitative variable acquired from two independent groups.

The results indicated that there is a statistically significant difference between the antioxidant effects of the rosemary group (∆_ROS_ABTS) and the antioxidant activity of the thyme group (∆_THYM_ABTS): *U* = 6.000; *z* = −1.922; *p* = 0.055 (Table 10).

In other words, if the histogram of ABTS scores is analyzed, the statistically significant difference between the mean ranks related to the two plant extracts can be confirmed. Thus, the increase in the antioxidant effect for the rosemary extract from the common crop compared to the control crop was significantly higher than the increase in antioxidant activity recorded for the thyme extract from the common crop compared to the control crop (mean rank _∆_ROS_ABTS_ = 8.50 > mean rank _∆_THYM_ABTS_ = 4.50).

Although the *p*-value exceeds the established α significance threshold (*p* = 0.055 > 0.05), we considered the evaluated difference between groups to be on the edge of significance, even if, from a theoretical point of view, it is non-significant. The *p*-value was interpreted in the context of the experiment conditions because statistical significance can be helpful to rank results rather than answer specific questions about the data sets (like “Is there an effect?”). So, in this case, we prefer to consider statistical significance as a gradual and continuous measure regarding a particular set of data, not a null hypothesis. The questions being asked (and answered) are of the form: “How strong is the difference?” or, even better, “Is the difference between crops strongly significant?” It is known that the smaller the *p*, the more significant the difference between crops. Furthermore, the statistical significance is higher when the *p*-value is closer to the alpha value. Therefore, we observed statistically significant differences in the growth of the rosemary and thyme antioxidant activity evaluated through the ABTS assay.

Descriptive statistics and normality tests for the FRAP method were applied to all data sets included in the study (Table S5).

The square root transformation was used for the variable ∆_THYM_FRAP since the skewness values exceeded the range [−1, +1] and outliers were identified outside the box plot distribution, which indicates insufficient normality for the use of parametric tests. The skewness values became fairly close to zero after variable transformation, and together with the Shapiro–Wilk normality test, the ∆_THYM_FRAP (sqrt) data set displayed a symmetrical distribution with no outliers in the box plot diagram.

The independent samples *t*-Test evaluated the difference between the optical density of the sample group and the optical density of the control group for the rosemary and thyme extracts after performing the FRAP determination method (Table S6).

Considering that the FRAP method for measuring antioxidant activity is less sensitive than the other methods (DPPH and ABTS), the *p*-value was estimated in this case at an alpha statistical significance level equal to 0.10 and a 90% confidence interval of the difference. In this way, it was possible to avoid ambiguous situations where the established significance level was not achieved.

Thus, a highly statistically significant difference (*p* < 0.1) was obtained between the increase in the optical density for the rosemary extract from the common crop compared to the control crop (∆_ROS_FRAP) and the increase in the optical absorbance recorded for the thyme extract from the common crop compared to the control crop (∆_THYM_FRAP (sqrt)): *p* = 0.078; t = 1.890.

Hedges’ g index was calculated to analyze the difference between the increases in optical density for the two types of plant extracts, estimating the standardized difference between the means. Hedges’ g index (Hedges’ g _FRAP_ = 0.871) represents the antioxidant effect size in standard-score units reported for the two independent groups using the *N* − 1 value, which is the sample-based standard deviation (Table 11).

The *t*-Test compared the optical density for the rosemary common crop and the rosemary control crop (FRAP_REF vs. FRAP_REM). Therefore, a statistically significant difference (*p* = 0.020; *p* < 0.05) was evidenced, which revealed considerable increases in the antioxidant activity of rosemary extract from the common crop compared to the control. The results were obtained at a 0.05 significance level and are stated in Table 12.

However, such substantial differences were not shown by the thyme extract.

Considering the statistical results obtained, the phytosociological batches of thyme and rosemary studied as common crops and cultivated together in cohabitation conditions proved enhanced antioxidant activity, unlike the control batches that were grown individually in the absence of mutual influence.

Overall, the rosemary vegetal extract exhibits the most statistically significant increase in the antioxidant effect by all applied methods (DPPH, ABTS, and FRAP) compared with the thyme extract.

#### 2.6.2. Pearson Correlation between Phenolic Constituents’ Content (TPCAs and TPC) and Antioxidant Activities (IC_50_ and EC_50_)

The aim of the Pearson correlation applied in this stage of the statistical analysis is to express the strength, magnitude, and direction of the linear relationship of correlation between the concentration of active phytoconstituents [g active principle/100 g dry extract] and the antioxidant activity [IC_50_ or EC_50_ value, mg/mL].

The data used for Pearson correlation are presented in Table 13.

The Pearson coefficients between each pair of experimental data were determined for different significance levels (1%, 5%, and 10%) and are presented in Table 14.

First, we observe a remarkable correlation (*r* > 0.900) between the experimentally applied methodologies (DPPH, ABTS, and FRAP) revealed by the Pearson coefficient (*r*) for each set of data pairs obtained by the different methods (DPPH vs. ABTS: *r* = 0.997, *p* = 0.003; DPPH vs. FRAP: *r* = 0.978, *p* = 0.022; ABTS vs. FRAP: *r* = 0.988, *p* = 0.012). Thus, the results obtained for the IC_50_ and EC_50_ values calculated through three methods for antioxidant activity evaluation are well correlated and statistically significant at the 0.05 level.

The Pearson correlation analysis indicates a robust and inverse correlation between total phenolic content (TPC) and the antioxidant effect of the plant extracts expressed as IC_50_ value. As the IC_50_ value is lower, the TPC is higher, and the extract has higher antioxidant activity (negative Pearson coefficient). The relationship is statistically significant at the 0.1 level only between total phenolic content and sensitive antioxidant methods like DPPH and ABTS (TPC vs. DPPH: *r* = −0.934, *p* = 0.066; TPC vs. ABTS: *r* = −0.937, *p* = 0.063), whereas between TPC and the EC_50_ value—in the FRAP method—the correlation is strong but not statistically significant (*r* = −0.887, −0.70 > *r* > −0.89).

Nevertheless, a strong inverse correlation was found between total phenolic acid content (TPCA) and the antioxidant activity assessed by the DPPH and ABTS methods (TPCAs vs. DPPH: *r* = −0.793, −0.70 > *r* > −0.89; TPCAs vs. ABTS: *r* = −0.765, −0.70 > *r* > −0.89) but it was non-statistically significant. The values of the Pearson coefficient are negative, which explains the inverse correlation between the data (the higher the concentration of phenolcarboxylic acids, the lower the IC_50_ value of the extracts; therefore, the stronger the antioxidant activity). Additionally, a moderate correlation was outlined by the Pearson coefficient (*r* = −0.657) for TPCAs vs. FRAP, emphasizing the existing relationship between the data even if the *p*-value failed to reach statistical significance.

These findings would suggest that TPCA contributes less to the antioxidant profile of rosemary and thyme extracts than TPC due to the statistically higher antioxidant impact of the TPC correlation compared to the antioxidant activity and TPCA correlation.

**Figure 4:**
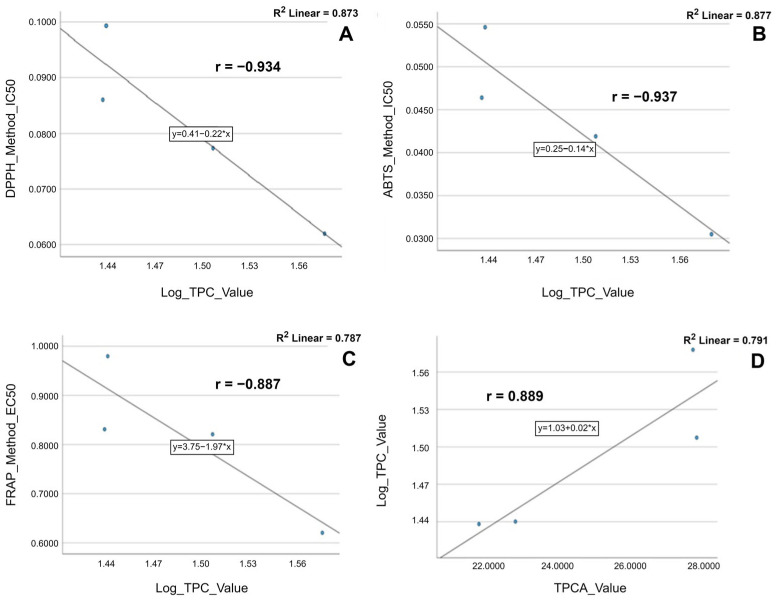
Pearson correlation scatter plot of the relationship between (**A**) DPPH and TPC; (**B**) ABTS and TPC; (**C**) FRAP and TPC; (**D**) TPC and TPCAs.

The Pearson correlation coefficient scatter plots of antioxidant activity and active phytoconstituents concentrations are shown in Figure 4.

**Figure 5:**
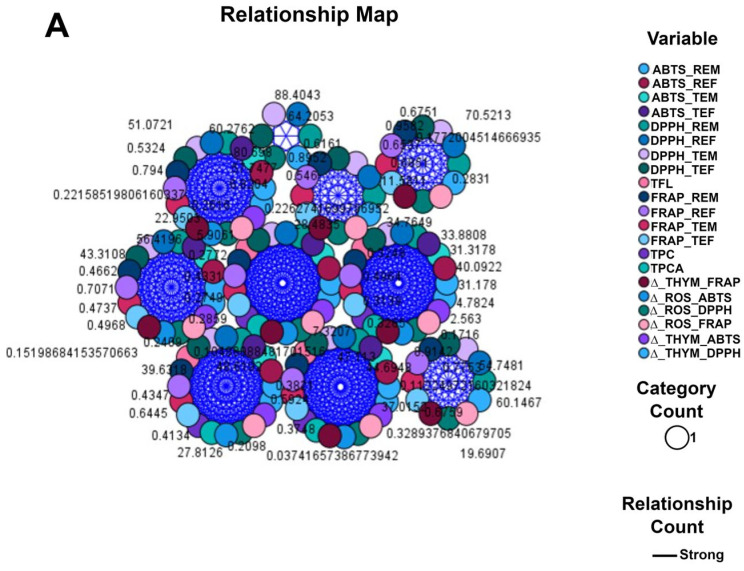

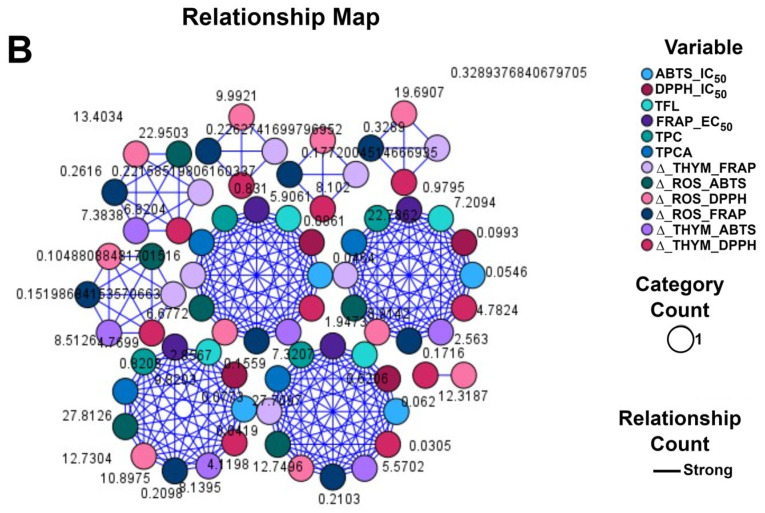
Relationship Maps—Circle Layout.

Relationship maps (Figure 5) were created to quantify the correlation between the antioxidant power and the concentration of active phytoconstituents in the plant extracts.

Relationship charts help to determine how variables relate by visualizing the connections and influences that each node and link has with the others [37].

In the first relationship map (Figure 5A), the connections between different antioxidant method results (ABTS, DPPH, and FRAP) of rosemary and thyme common and control extracts and the concentration of bioactive compounds (total phenolic content and total phenolic acid content), taking into account the increases in the antioxidant effect against free radicals, are represented. The thickness of each link, representing the strength of the relationship created, is so accentuated that the graph looks like a cloud of strongly correlated connections.

The second relationship chart (Figure 5B) emphasized the correlation between IC_50_ and EC_50_ values from different antioxidant methods and the high phytochemical levels of rosemary and thyme extract crops (common and control). We revealed an increase in the antiradical effect in common crops compared to the control ones for each vegetal extract. The symmetry of the node sizes for all tested variables confirmed the existing strong relationship. Moreover, it was evidenced by the uniformity of the connections and links.

#### 2.6.3. Correlations between Antioxidant Variables Expressed by Inhibition and Optical Density

We made a Pearson correlation between the concentration of active compounds and antioxidant inhibition or optical density (for the three methods) to evaluate if the initial data sets are strongly correlated. Using XLSTAT software 2022.2.1.1309, we analyzed the controls and common extracts through Pearson analysis, calculating *p*-values, correlation coefficient (*r*), and determination coefficient (*R*).

Data analysis shows a substantial and statistically significant correlation between antioxidant activities (expressed as inhibition or optical density) assessed through all three methods for all extracts (controls and common): *p <* 0.05–*p* < 0.001; and *r =* 0.042–0.001.

In control extracts, REM and TEM, all phenolic constituents (TPC, TFL, and TPCAs) meaningfully influence the antioxidant activity (*R* ≥ 0.981). Their role in FRAP for TEM and DPPH IC_50_ for REM is statistically significant (*p* < 0.05).

The same observation is seen for TEF and REF (*R* ≥ 0.964, *R* ≥ 0.983, respectively). Moreover, statistical significance is functional for FRAP in TEF (*p* = 0.032–0.031) and DPPH (*p* = 0.024) and ABTS (*p* = 0.008) in REF.

#### 2.6.4. Principal Component Analysis

**Figure 6:**
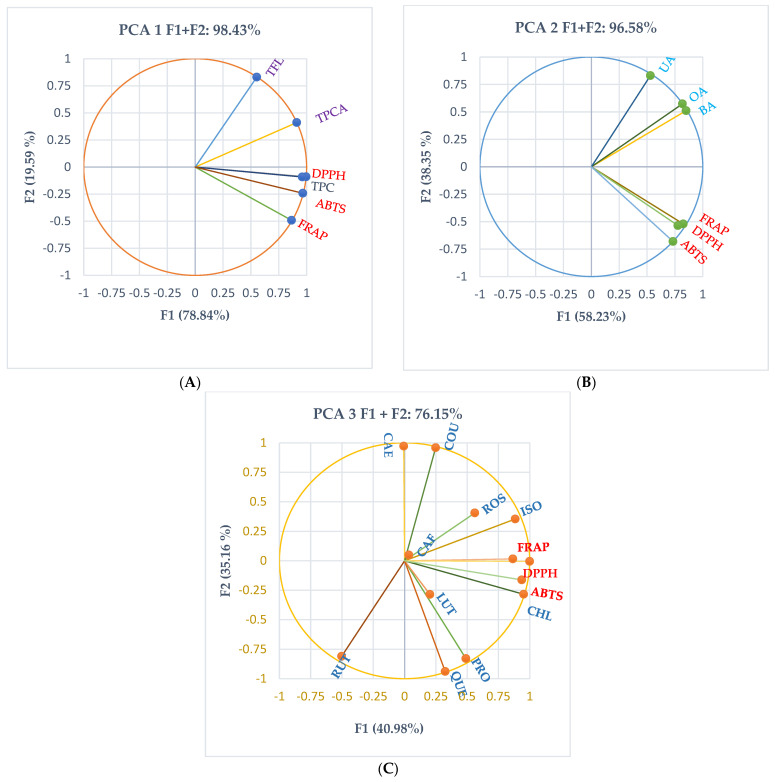
Principal component analysis (PCA). PCA correlation circle between quantified phytoconstituents of all rosemary and thyme extracts and antioxidant activity determined through various methods (DPPH, FRAP, ABTS): (**A**) PCA correlation circle between TPC, TPCAs, and TFL and antioxidant activity; (**B**) PCA correlation circle between triterpenic acids and antioxidant activity; (**C**) PCA correlation circle between individual polyphenolic compounds and antioxidant activity; TPC—total phenolic content, TPCAs—total phenolic acids content; TFL—total flavonoid content; PRO—protocatechuic acid, RUT—rutin, CAF—caffeic acid, CHL—chlorogenic acid, LUT—luteolin, KAE—kaempferol, ROS—rosmarinic acid, QUE—quercetin, ISO—isoquercitrin, FER—ferulic acid, COU—p-coumaric acid, BA—betulinic acid, OA—oleanolic acid, UA—ursolic acid.

Principal component analysis (PCA) was achieved for all extracts, and the correlations between various phytoconstituents and their antioxidant effects are illustrated in Figure 6.

Therefore, The PCA correlation circle from Figure 6A explains 98.42% of the data variances and correlates the TPC, TPCAs, and TFL with the plant extracts’ antioxidant activity. Figure 6A reveals a remarkable correlation between TPC and antioxidant activity evaluated through ABTS, DPPH, and FRAP assays: *r* = 0.954, *r* = 0.987, *p* < 0.05 and *r* = 0.908, *p* > 0.05, respectively. TPCAs show a good correlation with DPPH and ABTS (*r* = 0.839, *r* = 0.781, *p* > 0.05) and a moderate one with FRAP (*r* = 0.781, *p* > 0.05). TFL is poorly correlated with antioxidant activity: *r* = [0.438, 0.340, 0.077], *p* > 0.05 (Figure 6A).

The PCA correlation circle from Figure 6B explains 96.58% of data variances, correlating triterpenic acids (betulinic acid, oleanolic acid, and ursolic acid) with antioxidant activity. Generally, BA, OA, and UA show a low correlation with antioxidant activity in studied plant extracts: *r* < 0.5, *p* > 0.05 (Figure 6B).

The PCA correlation circle from Figure 6C explains 76.15% of data variances, correlating the individual polyphenols quantified in all plant extracts and their antioxidant activity. According to Figure 6C, chlorogenic acid is substantially associated with antioxidant activity evaluated through all methods: ABTS (*r* = 0.974, *p* < 0.05), DPPH, and FRAP (*r* = 0.948, *r* = 0.879, *p* > 0.05). Isoquercitrin is highly correlated with DPPH (*r* = 0.974, *p* < 0.05) and moderately with ABTS and FRAP (*r* = 0.675, *r* = 0.617, *p* > 0.05). Protocatechuic acid also reveals a moderate correlation with ABTS and DPPH (*r* = 0.510, *r* = 0.503, *p* > 0.05) while and rosmarinic acid only with DPPH (*r* = 0.584, *p* > 0.05). All other individual phenolic constituents poorly correlate with antioxidant activity (*r* < 0.5, *p* > 0.05, Figure 6).

**Figure 7:**
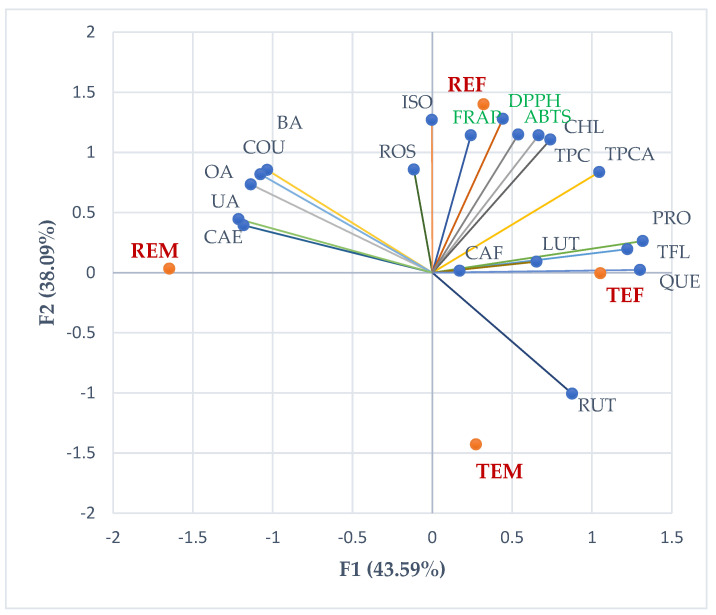
PCA correlation biplot. REM—rosemary extract, control crop; REF—rosemary extract, phytosociological (common) crop; TEM—thyme extract, control crop; TEF—thyme extract, phytosociological (common) crop; TPC—total phenolic content, TPCA—total phenolic acid content; TFL—total flavonoid content; PRO—protocatechuic acid, RUT—rutin, CAF—caffeic acid, CHL—chlorogenic acid, LUT—luteolin, KAE—kaempferol, ROS—rosmarinic acid, QUE—quercetin, ISO—isoquercitrin, FER—ferulic acid, COU—p-coumaric acid, BA—betulinic acid, OA—oleanolic acid, UA—ursolic acid.

As an overview, the correlation biplot from Figure 7 shows the place of all common and control extracts reported for their constituents and antioxidant activities.

### 2.7. Molecular Docking Studies

The molecular docking experiment investigated the potential of assessed phytochemicals to interact with the homodimeric BTB domains of transcription factors BACH1 and BACH2. Therefore, 11 compounds were screened against the target protein domains blindly. After docking with BACH1, kaempferol yielded the lowest binding energy (−7.31 kcal/mol), while protocatechuic acid showed the highest energy (−5.46 kcal/mol). On the other hand, the highest ligand efficiency was obtained for p-coumaric acid (0.514), while rutin had the lowest ligand efficiency (0.168). High values for predicted ligand efficiency were also observed for protocatechuic acid, caffeic acid, luteolin, kaempferol, quercetin, and ferulic acid (Table 15). For BACH2, the highest binding affinity was obtained for isoquercitrin (−7.97 kcal/mol) and protocatechuic acid recorded the lowest affinity (−5.37 kcal/mol). Like docking with the BACH1 BTB domain, *p*-coumaric acid showed the highest ligand efficiency (0.518), while rutin had the lowest value for this parameter (0.173).

**Figure 8:**
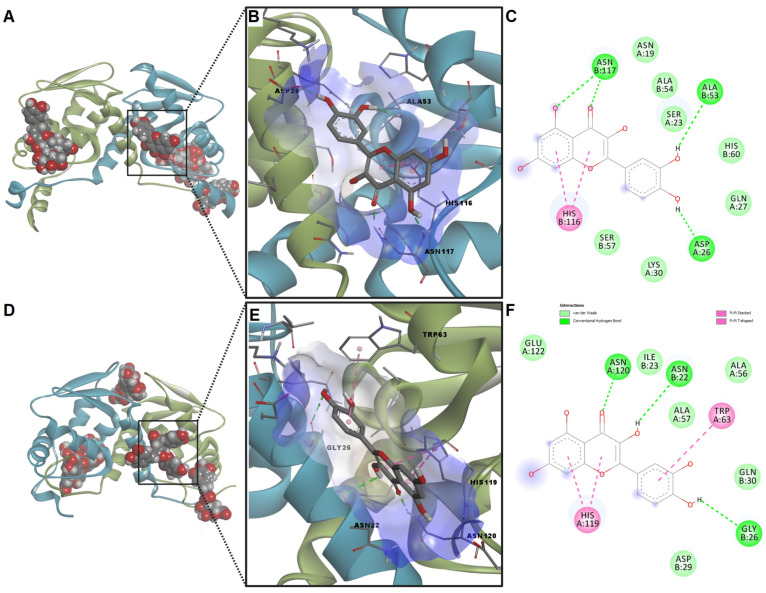
(**A**) Binding poses of screened phytochemicals after docking with BACH1 BTB domain; (**B**) conformation of predicted BACH1–quercetin complex; (**C**) interaction diagram for predicted BACH1–quercetin complex; (**D**) binding poses of screened phytochemicals after docking with BACH2 BTB domain; (**E**) conformation of predicted BACH2–quercetin complex; (**F**) interaction diagram for predicted BACH2–quercetin complex.

Even though both kaempferol and luteolin showed low binding energies and high ligand efficiency values, these compounds formed a low number of hydrogen bonds and non-polar interactions with the predicted binding sites. The molecular docking algorithm identified several potential binding areas (Figure 8A,D). Nonetheless, quercetin was the sole phytochemical that could bind to a common pocket for both BACH1 and BACH2 BTB domains. Quercetin formed pi–pi stacked interactions with histidine residues from both BACH1 (His116) and BACH2 (His119) through the benzopyran-4-one substructure (Figure 8B,C,E,F). The ketone moiety accepted hydrogen bonds from asparagine residues (Asn117 and Asn120). Moreover, quercetin formed another three hydrogen bonds through hydroxyl moieties with Asp29, Ala53, and Asn117 within BACH1. Moreover, quercetin interacted with BACH2 by forming hydrogen bonds with Asn22 and Gly26 and through pi–pi T-shaped interactions between Trp63 and the dihydroxyphenyl substructure.

## 3. Discussion

The novelty of the present study on the two previously described well-known medicinal plants consists in its different design. It started from the hypothesis that two medicinal plants from different species, containing approximately the same active phytoconstituents and coexisting in the same soil, can positively influence each other. Thus, their bioactive metabolite content increases, offering a significant therapeutic value. The present study is based on the premises of the existing data in the literature [36,38] and our previously published research [6,7,39]. Hence, we have investigated the extracts of *R. officinalis* and *T. vulgaris* grown in phytosociological crops (plant communities) and compared them to the control crops. These medicinal plants were grown in phytosociological crops and monitored in 2021. Data regarding crops’ establishment and evolution were previously published [6,7,39]. This study analyzed and compared the ethanol extracts of two medicinal plant products cultivated in phytosociological batches with those obtained from separately cultivated species control batches.

The outcomes of the present study agreed with previously published data from our research on plant raw materials [6,40]. The growth of the two species in phytosociological batches is beneficial for polyphenolic derivative synthesis.

The bioactive compounds of the four plant extracts (REM, REF, TEM, and TEF) were determined using spectrophotometric and chromatographic methods. Polyphenolic derivatives (TFL, TPCAs, and TPC) and triterpenic acids were identified and quantified. However, in all cases, spectrophotometric methods cannot be considered selective analysis methods due to possible interference with other constituents. They are frequently used in phytochemical analyses, being described including in the European Pharmacopoeia 11th edition (Chapter 2.8.14. Tannins in herbal drugs; dosage of flavones, methods described in various plant product monographs, for example, *Betulae folium*—expression in hyperoside; *Sambuci flos*—expression in isoquercitroside) [41].

Rosemary and thyme are aromatic plants from the same botanical family—*Lamiaceae*. Their common culture (phytosociological crop) positively influences the biosynthesis of specific secondary metabolites (polyphenolic compounds). The extracts obtained from these vegetal raw materials had a higher polyphenolic content than those obtained from the control samples. The solvent selected to obtain the extracts was 50% ethanol, based on previous research and literature [6,40,42,43,44] and using more ecological solvents that do not generate toxic metabolites.

The UHPLC–MS and FT–ICR MS analyses allowed the quantification of the content of polyphenols and triterpenic acids. The results indicate higher phenolic derivative content in the control samples. Caffeic acid content was 1.46 times higher in the control batch of rosemary compared to the phytosociological one; luteol content is also 1.62 times higher, and p-coumaric acid is 1.54 times higher. In the thyme extract, caffeic acid is 1.92 times higher and luteol 11.67 times higher in the phytosociological group. Small amounts were identified in all types of kaempferol extracts and thyme rutoside. Regarding triterpenic acids, it is found that they are in greater quantity in the control batches for rosemary and in the phytosociological ones for thyme.

The identified and dosed triterpenic acids, although they can induce, to a lesser extent, an antioxidant effect as shown by the absence of hydroxyl phenolic groups in their structure, can constitute a hydrogen reservoir for the regeneration of polyphenols directly involved in the antioxidant action [45]. We mention that among the triterpenic acids identified and dosed, betulinic acid could not be quantified in the thyme extract. The presence of caffeic acid in appreciable concentrations in all extracts is significant. Moreover, it is recognized for its antioxidant effect through various mechanisms (suppressing lipid peroxidation, completely blocking ROS production, and the xanthine/xanthine oxidase system). It also has antiviral, anti-inflammatory (inhibiting lipoxygenase activity), antitumor, immunomodulatory, and neuroprotective effects [46,47,48,49,50].

Luteol is cited in the scientific literature for its apoptotic effect and inhibition of cell proliferation but also for its chemopreventive effects due to its antioxidant action [51,52,53]. Interestingly, a compound can exert antioxidant action by inhibiting ROS generation or eliminating them.

Isoquercitrin increases SOD activity by up to 40%, upregulating the gene expression that encodes this endogenous antioxidant enzyme [54].

Other polyphenolic derivatives also contribute to the installation of the antioxidant effect in small quantities, probably based on the synergism of action.

Overall, the rosemary vegetal extract exhibits the most statistically significant increase in the antioxidant effect by all applied methods (DPPH, ABTS, and FRAP) compared with the thyme extract.

The antioxidant activity of vegetal extracts or plant sources is caused by different antioxidant components in plant tissue, which act as bioactive phytoconstituents that can scavenge free radicals. There are some chemical constituents present in vegetal extracts that were found in previous studies to exert a significant influence on the antioxidant effect [42,55,56,57,58,59].

Among these active phytoconstituents, total polyphenols and phenolcarboxylic acids are primarily those that can determine antioxidant activity at a noticeably higher degree [42].

Also, polyphenolic compounds and triterpenic acids are secondary metabolites of plants with an essential role in adaptation, growth, and resistance to diseases and pests, strengthening the defense systems of plants. Due to their biological and ecological significance, these active compounds are widely used in medicine with strong efficacy, especially for exerting antioxidant activity at the cellular level [60,61,62,63].

At the plant cultivation level, we found a way to amplify the antioxidant effect of rosemary and thyme. The target was to obtain chemically and therapeutically ennobled crops that are a good source of potent plant extracts. It may become essential in extracting active phytoconstituents and formulating quality plant extracts used in herbal medicine.

According to the results presented in this study, the plant extracts obtained from the common crop (plant community) showed higher concentrations of total polyphenols compared to the ones from the control crop, both for rosemary and thyme.

In the same way, a similar increase in the concentration of phenolcarboxylic acids was observed for the extracts from the common crops grown together compared to the control batch cultivated individually. The rosemary extract obtained from the common crop is the one that had the highest concentration of total polyphenols (378.336 ± 38.698 mg/g Eq expressed in tannic acid), unlike the thyme extract (321.678 ± 40.007 mg/g Eq expressed in tannic acid).

It was observed that the thyme extract from the common culture showed slightly increased TPCAs compared to rosemary (278.126 ± 45.049 mg/g Eq expressed in chlorogenic acid for TEF, compared to 277.097 ± 18.585 mg/g Eq expressed in chlorogenic acid for REF).

The spectrophotometric analysis also showed that the vegetal extracts have a variable amount of flavonoids, with concentration higher for the extracts obtained from the common crops than those obtained from the control groups. Therefore, TEF has the richest total flavonoid content (98.203 ± 13.646 mg/g Eq expressed in rutin), while REF has the lowest concentration of only 78.323 ± 12.470 mg/g Eq expressed in rutin.

The antioxidant activity was evaluated using three in vitro methods (DPPH, ABTS, and FRAP) and expressed as IC_50_ values. It could be observed that the antioxidant values (IC_50_) were significantly lower for the extracts from the common crops than those from the control ones, regardless of the type of antioxidant method. This means that the antioxidant effect of the extracts from common crops is higher than the antiradical activity of the extracts from control crops, considering that a lower IC_50_ value signifies a higher total antioxidant activity.

Between both plant extracts from the common crop, REF and TEF, rosemary extract was the most potent antioxidant, having the lowest IC_50_ values measured through all three antioxidant techniques (IC_50DPPH_ = 0.0620 mg/mL, IC_50ABTS_ = 0.0305 mg/mL, EC_50FRAP_ = 0.6206 mg/mL).

Furthermore, the comparative analysis of the antioxidant capacity of rosemary and thyme extracts with the control groups was assessed. Each extract showed different antiradical activities (expressed as free radical inhibition or optical density) due to their content of polyphenolic compounds and other phytochemicals.

Overall, the phytosociological batches of thyme and rosemary studied as a common group and cultivated together in cohabitation conditions were proved to have enhanced antioxidant activity, unlike the control batches that were grown individually without mutual influence.

The rosemary vegetal extract exhibits the most statistically significant increase (*p* < 0.05) in the antioxidant effect by all applied methods (DPPH, ABTS, and FRAP) compared with the thyme extract.

In addition, after calculating the Pearson coefficient between scavenging activity (IC_50_) and the concentration of therapeutically active phytochemical compounds, a powerful inverse correlation could be demonstrated between the concentration of total phenolic content (TPC) and the antioxidant effect. When the IC_50_ value is lower, the TPC concentration is higher, and the extract is a better antioxidant.

Concerning the link between TPCAs and IC_50_ antioxidant activity, a strong inverse correlation was found for TPCAs vs. DPPH and TPCAs vs. ABTS. In contrast, between TPCAs and FRAP EC_50_ a moderate inverse correlation was outlined by the Pearson *r* value, emphasizing the existing relationship between the data sets (the higher the concentration of phenolcarboxylic acids, the lower the IC_50_ or EC_50_ values of the extracts; therefore, the stronger the antioxidant activity).

Looking at the *p*-values obtained for the Pearson correlation between IC_50_ values and the content of bioactive compounds, our findings suggest that TPCAs have a minimal contribution to the antioxidant profile of rosemary and thyme extracts.

Nevertheless, our research suggests that total phenolic and total phenolic acid contents are the major contributors to the antioxidant effect of the analyzed vegetal extracts (assessed by all three methods). In contrast, total flavonoid content was poorly correlated and non-statistically significant with the antioxidant activity (rather than IC_50_ values or inhibition/optical density). This aspect could be explained by flavonoids’ glycosidic form, which demonstrates lower antioxidant activity than their aglycone.

We decided to perform two correlation analyses using the Pearson coefficient, one between the concentration of active phytoconstituents and IC_50_ antioxidant activities and another one between the concentration of active compounds and antioxidant inhibition or optical density for the three methods (without taking into account the IC_50_ values) to justify the magnitude of the association of compared variables, as a result of an overview from several perspectives.

Data correlation analysis highlighted a substantial and statistically significant relationship (*p* < 0.05 and *p* < 0.001) between antioxidant activities (expressed as inhibition or optical density) using all three methods (DPPH, ABTS, and FRAP) for all extracts (controls and common).

Although antioxidant activities can be expressed by the percentage of free radical scavenging activity (free radical inhibition), this percentage value of scavenging activity could not illustrate the real antioxidant effect of the analyzed extracts due to their complex chemical composition. So, plant extracts have many compounds. However, not all phytoconstituents exhibit antioxidant activities; some may act as antagonists of antioxidants and reduce the ability of antioxidant components.

Consequently, the only value that can more accurately approximate the antioxidant power of plant extracts is the IC_50_/EC_50_ value (an overview value), because it is calculated from the percentage inhibition or absorbance values using a linear regression equation. Then, the Pearson correlation between IC_50_ antioxidant activity and the concentrations of phytochemical compounds is more reliable. Several relationship diagrams were built to express how variables are related to each other and how strong the links created between them are.

Phytochemicals can exert in vivo antioxidant activities by directly interacting with reactive oxygen species or modulating key enzymes and transcription factors involved in the endogenous antioxidant defense. For instance, the antioxidant defense mechanisms can be triggered by stimulating the activity of Nrf2. However, the transcriptional activity of Nrf2 can be repressed by BACH1 and BACH2 [13]. To date, only a few small-molecule BACH1/2 inhibitors have been discovered. For instance, metalloporphyrins, cannabidiol, and HPP-4382 inhibit BACH1 activity by interacting with heme-binding domains within the transcription factor (CP motifs) [64,65], while 1,3-benzodioxin derivatives inhibit BACH1 by interacting with the BTB domain [66].

The molecular docking studies revealed that quercetin (found in both thyme and rosemary extracts obtained after phytosociological cultivation) could bind to BTB domains of both BACH1 and BACH2. This link is possible by interacting with histidine and asparagine residues which are conserved between the two transcription factors. Unfortunately, the lack of crystal structures of known inhibitors in complexes with BACH1/BACH2 limits the reliability of the performed simulations. No other studies have investigated the potential direct interaction between quercetin or other polyphenols and BACH1/BACH2 through in silico approaches. However, the putative binding site we identified for quercetin is adjacent to the previously proposed site for the 1,3-benzodioxin derivatives. Future studies must validate our findings and evaluate quercetin’s capacity to induce heme oxygenase-1 transcription by BACH1/BACH2 derepression.

## 4. Materials and Methods

### 4.1. Materials

All chemicals and reagents were of analytical grade. Ethanol, sodium acetate, aluminum chloride, hydrochloric acid, sodium hydroxide, sodium carbonate, Arnow and Folin–Ciocâlteu reagents, rutin, chlorogenic acid, rosmarinic acid, rutin, quercetin, tannic acid, phosphate buffer, DPPH, potassium persulfate, ABTS, K_3_(FeCN)_6_, and trichloroacetic acid were supplied from Sigma-Aldrich Chemical Company (Saint Louis, MO, USA).

Merck KGaA (Darmstadt, Germany) provided the HPLC gradient-grade solvents (water, acetonitrile, ammonium formate) and the following standards: luteolin and kaempferol. Alfa Aesar GmbH & Co., KG (Karlsruhe, Germany) was the manufacturer and supplier of other standards: protocatechuic acid, caffeic acid, p-coumaric acid, and ferulic acid. At the same time, isoquercitrin was obtained from HWI Analytik (Rheinland-Pfalz, Germany).

#### 4.1.1. Plant Materials

In 2018, we grew two medicinal plants, *Rosmarinus officinalis* L. and *Thymus vulgaris* L., as typical (phytosociological) crops. Rosemary and thyme were planted using experimental plots with the following specifications: area of (50 × 300) cm, 400 cm between batches, 30 cm between seedlings, and 5 seedlings per group. Romanian suburbs of the city of Turnu Măgurele in Teleorman County (43°44′44.16″ northern latitude, 24°52′53.40″ eastern longitude) were used to cultivate the batches [6]. This region experiences 11.5 °C on average per year, 23 °C monthly for the warm months, and less than 2 °C for the cold months. It stands out for its high caloric potential, amplitudes of high air temperature, minimal precipitation, torrential summertime regime that occurs frequently, and numerous dry spells.

The control crops were planted apart from the common culture to prevent them from being impacted (established at a distance of 25–30 m from the common crop and between them to avoid any subsequent influence).

The morphological and phytochemical properties of the harvest were examined by contrasting each crop with the control batch [7,40]. Each year in July, the plants were gathered and dried at a lab at the Faculty of Pharmacy’s Department of Pharmacognosy, Phytochemistry, and Phytotherapy, Carol Davila University of Medicine and Pharmacy, Bucharest. The dried plants were kept in paper bags in a room with controlled humidity, no higher than 65%, the moisture of plant products being a maximum of 10% (according to Ph. Eur. [67,68]) at a temperature of 20–25 °C.

The study was conducted between 2018 and 2021, and the present work contains data collected in 2021.

#### 4.1.2. Plant Extracts

Two sequential reflux extraction processes were performed on 25 g of each herbal product. The first extraction used 1.5 L of solvent for 30 min and the second 750 mL of solvent for the same time. The two extract solutions were combined, concentrated in a rotary evaporator (Vacuum Pump V-700, Buchi Labortechnik, Flawil, Switzerland), and lyophilized (ALPHA 1–2 LDplus freeze dryer, Martin Christ Gefriertrocknungsanlagen GmbH, Osterode am Harz, Germany).

The dry extracts were kept in a glass vacuum desiccator to not capture moisture from the atmosphere. The samples were marked as follows: RE—rosemary extracts, TE—thyme extracts [42], REM—rosemary extract from the control crop, REF—rosemary extract from the common crop, TEM—thyme extract from the control crop, and TEF—thyme extract from the common crop. The solvent used for polyphenolic compound extraction was 50% ethanol for both medicinal plants, ensuring the most effective process [43,69].

The raw materials’ quality was evaluated using established and typical spectrophotometric techniques. The identity of the analyzed chemicals in the plant raw materials was also validated using the UHPLC–MS and HR ESI–MS techniques.

### 4.2. Phenolic Constituent Analysis

Total flavonoid content (TFL), total phenolic acid content (TPCA), and total phenolic content (TPC) were all determined using spectrophotometric techniques.

#### 4.2.1. Determination of Total Flavonoid Content

The TFL analysis was performed using a colorimetric approach based on the reaction of flavonoids with AlCl_3_. Aliquots of 0.2 g extract were dissolved in 25 mL of 50% ethanol for REM, REF, TEM, and TEF. Volumes of 0.1 mL, 0.2 mL, 0.3 mL, 0.4 mL, and 0.5 mL were put into volumetric flasks of 10 mL. Subsequently, 2 mL of 100 g/L sodium acetate and 1 mL of 25 g/L aluminum chloride solution were added. The volumes were then adjusted to 10 mL by adding the same solvent (ethanol 50%). Parallel to the samples to be tested, appropriate control samples were generated under the same conditions but without sodium acetate or aluminum chloride. We considered relevant control samples to be the control solutions for which we had only extractive solutions (the same volumes used above for the sample preparation) diluted with solvent without using other reagents.

After 45 min, the absorbance at 427 nm was measured using a Jasco V-530 spectrophotometer (Jasco Corporation, Tokyo, Japan). Rutin was utilized as a standard for the linear calibration curve with R^2^ = 0.9992 in the concentration range of 5–35 g/mL (Supplementary Materials Figure S1). The extract’s total flavonoid concentration (TFL) was reported as mg rutin equivalents per gram of sample (mg/g) [70].

#### 4.2.2. Determination of Total Phenolic Acid Content

The TPCAs were assessed using their ability to generate nitro derivatives with nitrous acids. Approximately 0.2 g of each dry extract was dissolved in 25 mL of 50% ethanol. Into 10 mL volumetric flasks, volumes of 0.05 mL, 0.1 mL, 1.15 mL, 0.2 mL, and 0.25 mL were poured. Then, 2 mL of 0.5 M hydrochloric acid, 2 mL of Arnow reagent, and 2 mL of sodium hydroxide 85 g/L were added and adjusted to 10 mL with distilled water. The absorbance was measured immediately at 525 nm using a Jasco spectrophotometer and compared to a sample that did not include the Arnow reagent. Chlorogenic acid was used as a standard for the calibration curve in the linear range of 11–53 μg/mL with R^2^ = 0.9998 (Figure S2 from Supplementary Materials). The total phenolic acid content of the extracts was expressed as mg chlorogenic acid equivalents per gram of sample (mg/g) [70].

#### 4.2.3. Determination of Total Phenolic Content

The TPC was determined following Lamuela–Raventós’ [71] methodology with minor adjustments. This technique used 100 mL of 50% ethanol to dissolve the same amount of each dry extract (0.1 g). For REM and REF extracts, volumes of 0.1 mL, 0.2 mL, 0.3 mL, 0.4 mL, and 0.5 mL were taken, adjusted to 1 mL by adding distilled water, and transferred to 10 mL volumetric flasks. Despite that, for TEM and TEF, we utilized different volumes. We put 0.25 mL, 0.35 mL, 0.45 mL, 0.55 mL, and 0.65 mL into 10 mL volumetric flasks and added distilled water to make them 1 mL. Afterward, 1 mL of Folin–Ciocâlteu reagent was added to the mixture, and it was then kept at 25° C for 5–8 min before 8 mL of sodium carbonate solution (200 g/L) was added. The absorbance was measured at λ = 725 nm using a Jasco V-530 spectrophotometer after 40 min in complete darkness. The absorbance value was calculated compared to a blank (a mixture of 1 mL distilled water and 1 mL Folin–Ciocâlteu reagent that had been increased to 10 mL by adding sodium carbonate). The same method was applied to obtain the tannic acid standard curve. With an R^2^ value of 0.999 and a linear concentration range of 2 to 9 μg/mL, tannic acid was employed as the standard for the calibration curve (Supplementary Materials Figure S3). Tannic acid equivalents per gram of sample (mg/g) were used to measure the total phenolic content [70].

#### 4.2.4. Identification and Quantification of Polyphenolic Compounds by Ultra-High Performance Liquid Chromatography–MS (UHPLC–MS)

All separations were performed on the ACQUITY Arc System equipped with an ACQUITY QDa Detector and a CORTECS C_18_ column, 4.6 × 50 mm, 2.7 μm. The mobile phase consisted of two solvents (acetonitrile and water) in which ammonium formate was dissolved to obtain a 10 mM concentration. Ammonium formate 10 mmol (solvent A) and acetonitrile (solvent B) had a flow rate of 0.5 mL/min. The volume injected was 5 μL and the analysis time was 21 min. The gradient conditions were: 0 min 8% B, 8 min 20% B, 16 min 27% B, 19 min 60% B, 20 min 60% B, and 21 min 8% B. The following standards were used: protocatechuic acid, caffeic acid, *p*-coumaric acid, ferulic acid, chlorogenic acid, rosmarinic acid, rutin, quercetin, luteolin, kaempferol, and isoquercitrin. Empower 3 Software was used for data acquisition and processing. This method’s calibration curve with standard chromatogram, retention time, and other details can be found in the Supplementary Materials, Section S1.2 (Tables S1 and S2, Figure S4).

#### 4.2.5. Identification of Polyphenolic Compounds by High-Resolution Electrospray Ionization Mass Spectrometry (HR ESI–MS)

A Fourier-transform ion cyclotron resonance mass spectrometer (FT–ICR MS) equipped with a 15T superconducting magnet—solar X-XR, QqqFT–ICR HR (Bruker Daltonics GmbH & Co., KG, Bremen, Germany)—was used for the HR ESI-MS [72].

The sample was precisely injected with a flow rate of 120 µL/h for negative and positive ESI ionization [73]. For ESI, the parameters were: capillary 5700 V, end plate offset 800 V, carrier gas pressure (N_2_) of 4 bar at 180^0^ C, having a flow rate of 7 L/min; the mass range was measured from 46–800 amu. For ESI+, the different parameters were: carrier gas pressure (N_2_) under 3.2 at 180 °C, with a sample flow rate of 7 L/min [7].

To analyze the chemical composition of small-molecule organic complex mixtures, FT–ICR MS has become a more effective method [74,75]. This method offers the best resolution performance and ultra-high mass-to-charge ratio (*m*/*z*) accuracy of each analyte, including plant metabolomics detected without chromatographic separation stages [76,77,78].

### 4.3. In Vitro Antioxidant Activity

DPPH-free radical scavenging, ABTS-based antioxidant capacity, and ferric-reducing antioxidant power (FRAP) are among the three most popular methods commonly accepted and routinely practiced in research laboratories to date [79,80,81].

#### 4.3.1. 2,2-Diphenyl-1-picrylhydrazyl (DPPH) Free Radical-Scavenging Method

DPPH-scavenging activity was measured to evaluate the antioxidant effect of the analyzed extracts. The DPPH concentration was reduced in the presence of an antioxidant compound at 515 nm, and the optical density gradually disappeared with time. The extractive solutions were prepared by dissolving 0.1 g of each dry extract in 100 mL of 50% ethanol. Then, volumes of 0.3 mL, 0.4 mL, 0.5 mL, 0.6 mL, 0.7 mL, 0.8 mL, 0.9 mL, 1.0 mL, 1.1 mL, and 1.2 mL of each obtained solution were adjusted to 10 mL in volumetric flasks with 50% ethanol. After this, to the 0.5 mL of each dilute solution prepared as above, 3 mL of DPPH 0.1 mM was added. After 30 min in darkness, the absorbance of the samples (A_sample_) was measured against the ethanol blank at 515 nm wavelengths using a Jasco V-530 spectrophotometer. Ascorbic acid was the reference with a 2–22 µg/mL concentration range, and the standard curve was made (Figure S5).

The following equation was used to compute the percentage of DPPH^•^ inhibition:

(1) $$\%\;DPPH\;free\;radical\;scavenging=\frac{A\;\left(blank\right)\;-\;A\;\left(sample\right)}{A\;\left(blank\right)}\times 100$$

$A\;\left(blank\right)$—blank (ethanol) absorbance of DPPH 0.1 mM solution in the absence of extracts (1.00 ± 0.10); $A\;\left(sample\right)$—specimen absorbance of the DPPH solution in the presence of plant extracts.

Inhibition curves (%) were constructed depending on the concentration (mg/mL), and the IC_50_ (mg/mL) values were calculated from the linear equations for each extract, taking the value y = 0.5.

#### 4.3.2. ABTS Method

The reaction agent (the free radical ABTS^•+^) was obtained following the reaction of the ammonium salt of 2,2-azino-bis(3-ethyl-benzothiazoline-6-sulfonic acid) with potassium persulfate (PP) [82]. So, the blue radical was pre-generated a day before by mixing the PP and ABTS to stand overnight for 16 h in darkness at room temperature [83].

We previously described the ABTS working method and the calculation of radical inhibition [7] used to assess the IC_50_ (mg/mL) value. The ABTS free radical-scavenging activity was expressed as inhibition percent (I%). The inhibitory concentration (IC_50_) needed to scavenge 50% of the ABTS^•+^ free radical was evaluated from the linear equation “inhibition − concentration.” A lower IC_50_ value corresponds to a stronger antiradical activity. A trolox calibration curve was used as a reference (Figure S6).

#### 4.3.3. Ferric-Reducing Antioxidant Power Assay (FRAP Assay)

The FRAP assay is based on the ability of antioxidant compounds to reduce Fe^3+^ ions to Fe^2+^, with a color shift from orange to blue. A modified procedure assessed plant extracts’ ferric-reducing capacity [80].

The test used extractive solutions prepared from 0.1 g of dry extracts dissolved in 100 mL of 50% ethanol for every vegetal extract. Volumes of 0.4 mL, 0.5 mL, 0.6 mL, 0.7 mL, 0.8 mL, 0.9 mL, 1.0 mL, 1.1 mL, and 1.2 mL of each obtained solution were poured into volumetric flasks and adjusted to 10 mL by adding the same solvent (50% ethanol). From each previously prepared dilution, volumes of 2.5 mL were taken and added to test tubes. Then, they were mixed with 2.5 mL of phosphate buffer (pH 6.6) and 2.5 mL of K_3_(FeCN)_6_ 1% and kept in a water bath at 50 °C for 20 min. Then, 2.5 mL of 10% trichloroacetic acid was added. Next, 2.5 mL of each solution was combined with 2.5 mL of distilled water and 0.5 mL of 0.1% FeCl_3_. The mixture was left to react for 10 min.

Absorbances were measured on a Jasco V-530 spectrophotometer (Jasco, Tokyo, Japan) at a wavelength of λ = 700 nm against a blank of 5 mL of distilled water and 0.5 mL of 0.1% FeCl_3_. A ferrous sulfate calibration curve was used as a reference (Figure S7).

The antioxidant capacity was determined using the EC_50_ value (mg/mL), which represents the concentration of the solutions to be analyzed at which the absorbance has a value of 0.5 (EC_50_ measures the concentration of a compound that manifests half of its antioxidant effect, EC—effective concentration). The EC_50_ value (mg/mL) is determined from the equation of the regression line concentration (mg/mL) vs. absorbance (for y = 0.5).

As our previously published work demonstrated, different extract volumes were tested to reach the absorbance value of 0.5 due to the variability of plant characteristics and the non-uniformity of pharmacognostic profiles of plant extracts. Experimental values closer to the target value result in a more accurate approximation (IC_50_ for y = 0.5). As mentioned above, the optimized values have been set to conduct an appropriate comparative study within the same technique and other methods of assessing the antioxidant activity [7,42].

### 4.4. Molecular Docking

Molecular docking studies were carried out to assess the potential interactions between identified natural compounds and transcription factors BACH1 and BACH2. Crystal structures of homodimeric BTB domains of BACH1 and BACH2 were retrieved from the RCSB PDB database (PDB IDs: 2IHC [84] and 3OHV [85]). The protein structures were prepared with YASARA Structure [86] by removing water molecules, correcting structural errors, protonation according to the physiological pH (7.4), and optimizing the hydrogen-bonding network and energy minimization. All purposes were assessed using YASARA2 forcefield. Considering there are no readily available crystal structures of BACH1/2 in complex with inhibitors, no validation of the docking protocol could be performed, which limits the current study’s reliability.

Ligands (7 natural phenolic compounds and 4 hydroxycinnamic acids) were prepared by generating 3D structures with DataWarrior 5.2.1 [87]. Ligand structures were energetically minimized using MMFF94s+ forcefield and were protonated according to the physiological pH. The AutoDock Vina v1.1.2 docking algorithm [88] performed 12 runs for each compound. The grid box was set to include the entire protein structure to identify potential binding pockets for the natural ligands. The docking results were retrieved as the best binding pose’s binding energy (ΔG, kcal/mol) and ligand efficiency (LE, ΔG/no. of heavy atoms). The predicted molecular interactions between the ligands and target proteins were analyzed using BIOVIA Discovery Studio Visualizer (BIOVIA, Discovery Studio Visualizer, Version 17.2.0, Dassault Systèmes, 2016, San Diego, CA, USA).

### 4.5. Statistical Analysis

Statistical analysis was performed using IBM SPSS Statistics Software version 29.0.0.0 (241) (IBM Corporation, Chicago, IL, USA). For each set of experimental data, the necessary conditions for applying statistical tests were evaluated, such as the data’s normality and the variances’ homogeneity. The normal distribution of the data was assessed by the Shapiro–Wilk test, the Kolmogorov–Smirnov test, and histograms. The skewness values also supported the results of the normality tests. When specific experimental data deviate from the Gaussian distribution, they are transformed (by reciprocal or square root) to become normally distributed and be subjected to parametric statistical tests. Considering that the main indicators for the choice of the type of transformation are the unit, the experimental design, and the possible robustness of F statistics to “small deviations” from normal, the results were checked after applying transformations and then compared to see if these transformation options could really change the distribution of data and improve experimental accuracy.

The independent samples *t*-Test [89] was applied to compare normally distributed data sets. In addition, the independent samples Mann–Whitney U test was used as a non-parametric test for strongly non-normal data (data resistant to the transformation method).

Levene’s test [90] for equality of variances was used to verify the homogeneity of variances for the experimental data sets (equal variances assumed or not assumed). The Welch *t*-Test and Brown–Forsythe’s test were run as robust tests when variances’ homogeneity was violated.

The Pearson correlation and principal component analysis (PCA) [91] were performed using XLSTAT 2022.2.1.1309 by Addinsoft (New York, NY, USA) [92] and examined the correlations between variable parameters [93].

Another analysis correlated bioactive phytochemicals’ concentration with antioxidant activity expressed by IC_50_/EC_50._ It consisted of bivariate analysis in SPSS, relationship maps, and scatter plots to confirm the antioxidant impact exerted by active compounds. Interpretations were made after the mandatory application criteria were met. In all cases, the significance level was set at 0.05. The results are considered significant when *p* < 0.05.

## 5. Conclusions

The proposed aim of our study was achieved by highlighting the positive impact of cultivation in phytosociological crops (plant communities) on antioxidant activities of medicinal plants, supporting their use as potential antioxidant therapies. We showed that cultivating both species in plant communities increases the biosynthesis of phenolic secondary metabolites, leading to phytochemically enriched extracts. Therefore, secondary metabolites’ concentrations in dry extracts obtained from plants harvested from phytosociological crops were higher than those from the control crops, translating into a superior antioxidant activity. Thus, the cultivation of two *Lamiaceae* medicinal plants (rosemary and thyme) in plant communities positively influenced their antioxidant potential.

The present study adds valuable insight for the drug development community by investigating a strategy based on phytosociological considerations for enhancing the therapeutic potential of medicinal plants. Further research will expand the phytochemical analyses and correlate the polyphenols’ content with the antioxidant effect for the extracts obtained from plant species belonging to the same family or from different families cultivated in plant communities. Moreover, additional studies are warranted to investigate the predicted potential of quercetin’s ability and other phytoconstituents to induce Nrf2 transcriptional activity through BACH1/BACH2 derepression.

## Figures and Tables

**Table 1:** Quantitative analysis of polyphenols in plant extracts.

Plant Extract	TFL (mg/g Eq Expressed in Rutin)	TPCAs (mg/g Eq Expressed in Chlorogenic Acid)	TPC (mg/g Eq Expressed in Tannic Acid)
REM	59.061 ± 4.01	217.756 ± 43.13	236.058 ± 17.88
REF	78.323 ± 12.47	277.097 ± 18.60	378.336 ± 38.70
TEM	72.094 ± 18.04	227.862 ± 36.85	275.507 ± 22.57
TEF	98.203 ± 13.65	278.126 ± 45.05	321.678 ± 40.01

Total flavonoid content (TFL), total phenolic acid content (TPCA), total phenolic content (TPC). Results are expressed as mean ± SD (*n* = 5). REM—rosemary extract, control crop; REF—rosemary extract, common crop; TEM—thyme extract, control crop; TEF—thyme extract, common crop.

**Table 2:** Individual polyphenol concentrations (μg compound/g extract) quantified by UHPLC–MS in each extract.

Sample Name	REM	REF	TEM	TEF
Protocatechuic acid	16.820 ± 1.25	39.442 ± 1.03	32.742 ± 1.09	47.581 ± 1.50
Rutin	ND	ND	12.734 ± 1.22	11.776 ± 0.93
Caffeic acid	524.433 ± 1.22	357.742 ± 1.87	356.001 ± 1.73	684.231 ± 1.56
Chlorogenic acid	21.507 ± 1.15	68.022 ± 1.53	23.873 ± 0.60	46.341 ± 1.27
Luteolin	87.172 ± 1.09	53.355 ± 0.81	29.278 ±0.92	341.923 ± 1.25
Kaempferol	4.934 ± 0.75	2.485 ± 0.78	1.492 ± 0.46	2.312 ± 0.61
Rosmarinic acid	34.681 ± 1.41	33.204 ± 1.46	27.485 ± 1.80	35.947 ± 1.58
Quercetin	ND	19.793 ± 1.12	18.933 ± 1.36	20.057 ± 1.90
Isoquercitrin	143.862 ± 1.55	167.290 ± 1.91	85.778 ± 0.75	151.538 ± 0.88
Ferulic acid	ND	ND	ND	ND
*p*-Coumaric acid	61.683 ± 1.13	39.832 ± 1.85	5.174 ± 1.61	8.021 ± 1.18

REM—rosemary extract, control crop; REF—rosemary extract, phytosociological (common) crop; TEM—thyme extract, control crop; TEF—thyme extract, phytosociological (common) crop, results are expressed as mean ± SD (*n* = 3), ND—not detected.

**Table 3:** Polyphenols identified by FT–ICR MS in rosemary and thyme extracts.

Sample Name	*m*/*z*		REM		REF		TEM		TEF	
	ESI+	ESI−	ESI+	ESI−	ESI+	ESI−	ESI+	ESI−	ESI+	ESI−
Protocatechuic acid	155.033	153.019	+	−	+	−	+	−	+	−
Rutin	611.160	609.146	+	−	+	−	+	+	+	+
Caffeic acid	181.049	179.034	+	+	+	+	+	+	+	+
Chlorogenic acid	355.102	353.087	+	+	+	+	+	−	+	−
Luteolin	287.055	285.040	+	+	+	+	+	+	+	+
Kaempferol	287.055	285.040	+	+	+	+	+	+	+	+
Rosmarinic acid	361.091	359.077	+	+	+	+	+	+	+	+
Quercetin	303.049	301.035	−	+	+	+	+	+	+	+
Isoquercitrin	465.102	463.088	+	+	+	+	+	+	+	+
Ferulic acid	195.065	193.050	+	+	+	+	−	+	−	+
*p*-Coumaric acid	165.054	163.040	−	+	−	+	+	+	+	+

REM—rosemary extract, control crop; REF—rosemary, phytosociological (common) crop; TEM—thyme extract, control crop; TEF—thyme extract, phytosociological (common) crop, “+”: the compound was found; “−“: the compound was not found.

**Table 4:** Quantification of pentacyclic triterpenic acids in plant extracts by UHPLC–MS method.

Plant Extract		REM	REF	TEM	TEF
Triterpenic acids (μg compound/g extract)	BA	10.972 ± 1.13	8.754 ± 0.72	ND	ND
	OA	11.446 ± 0.93	8.511 ± 0.63	1.302 ± 0.21	1.952 ± 0.14
	UA	11.672 ± 0.81	6.224 ± 0.72	3.503 ± 0.43	4.912 ± 0.24

REM—rosemary extract, control crop; REF—rosemary extract, phytosociological crop; TEM—thyme extract, control crop; TEF—thyme extract, phytosociological crop; BA—betulinic acid, OA—oleanolic acid, UA—ursolic acid, ND—not detected.

**Table 5:** The antioxidant effect of all plant extracts expressed as % DPPH free radical scavenging.

DPPH_REM	DPPH_TEM	DPPH_REF	DPPH_TEF	∆_ROS_DPPH	∆_THYM_DPPH
26.8293	28.2696	29.6860	30.2169	2.8567	1.9473
32.2557	29.9825	37.8352	34.7649	5.5795	4.7824
36.3526	32.1799	47.1130	37.7501	10.7604	5.5702
38.1699	35.5120	49.0674	39.6318	10.8975	4.1198
42.0245	38.5409	51.9401	43.3108	9.9156	4.7699
46.8293	44.2517	60.2327	51.0721	13.4034	6.8204
53.4162	46.8773	63.4083	58.4584	9.9921	11.5811
54.4747	50.8127	70.5213	58.9147	16.0466	8.1020
60.1467	54.7481	79.8374	66.5479	19.6907	11.7998
64.2053	56.3985	88.4043	68.7172	24.1990	12.3187

DPPH_REM—inhibition exerted by rosemary extract, control crop; REF—rosemary extract, common crop; TEM—thyme extract, control crop; TEF—thyme extract, common crop; ∆_ROS_DPPH = DPPH_REF − DPPH_REM, ∆_THYM_DPPH = DPPH_TEF − DPPH_TEM.

**Table 6:** The antioxidant effect expressed as ABTS free radical inhibition (%) for the plant extracts included in the study.

ABTS_REM	ABTS_TEM	ABTS_REF	ABTS_TEF	∆_ROS_ABTS	∆_THYM_ABTS
23.3391	21.1628	30.0163	28.4835	6.6772	7.3207
31.1780	31.3178	40.0922	33.8808	8.9142	2.5630
37.0152	34.0213	49.7648	44.6948	12.7496	10.6735
47.3598	40.4797	60.0902	48.6192	12.7304	8.1395
54.1619	47.9070	64.9578	56.4196	10.7959	8.5126
57.7477	52.8924	80.6980	60.2762	22.9503	7.3838

ABTS_REM—inhibition exerted by rosemary extract, control crop; REF—rosemary extract, common crop; TEM—thyme extract, control crop; TEF—thyme extract, common crop; ∆_ROS_ABTS = ABTS_REF − ABTS_REM; ∆_THYM_ABTS = ABTS_TEF − ABTS_TEM.

**Table 7:** The antioxidant effect expressed as optical density using the FRAP assay for the plant extracts included in the study.

FRAP_REM	FRAP_TEM	FRAP_REF	FRAP_TEF	∆_ROS_FRAP	∆_THYM_FRAP
0.2772	0.2749	0.4331	0.2859	0.1559	0.0110
0.3248	0.3139	0.4964	0.3265	0.1716	0.0126
0.3821	0.3734	0.5924	0.3748	0.2103	0.0014
0.4347	0.4147	0.6445	0.4134	0.2098	−0.0013
0.4662	0.4737	0.7071	0.4968	0.2409	0.0231
0.5324	0.5048	0.7940	0.5539	0.2616	0.0491
0.6161	0.5460	0.8952	0.5972	0.2791	0.0512
0.6751	0.6537	0.9582	0.6851	0.2831	0.0314
0.7753	0.6759	1.1042	0.7841	0.3289	0.1082

FRAP_REM—optical density obtained for rosemary extract, control crop; REF—rosemary extract, common crop; TEM—thyme extract, control crop; TEF—thyme extract, common crop; ∆_ROS_FRAP = FRAP_REF − FRAP_REM; ∆_THYM_FRAP = FRAP_TEF − FRAP_TEM.

**Table 8:** Independent Samples *t*-Test for DPPH method.

		Levene’s Test		*t*-Test for Equality of Means							
		F	Sig.	t	*df*	Significance		Mean Diff.	Std. Error Diff.	95% CI of the Difference	
						One-Sided *p*	Two-Sided *p*			Lower Bound	Upper Bound
Score	Equal variances assumed	1.807	0.196	2.231	18	0.019	0.039 *	5.1530	2.3097	0.3004	10.0055

* The significance level is 0.05.

**Table 9:** Independent Samples Effect Sizes for the DPPH method.

		Standardizer ^a^	Point Estimate	95% Confidence Interval	
				Lower	Upper
Score	Cohen’s d	5.1646986	0.998	0.051	1.920
	Hedges’ correction	5.3931164	0.9555 *	0.049	1.839
	Glass’s delta	3.6361205	1.417	0.304	2.480

^a^ The denominator used in estimating the effect sizes. * Hedges’ correction uses the pooled standard deviation plus a correction factor.

**Table 10:** Independent Samples Mann–Whitney U Test Summary.

Mann–Whitney *U*	6.000
Wilcoxon *W*	27.000
Test Statistic	6.000
Standard Error	6.245
Standardized Test Statistic (*z*)	−1.922
Asymptotic Sig. (2-sided test)—*p*-value *	0.055

* The significance level is 0.05.

**Table 11:** Independent Samples Effect Sizes for FRAP method.

		Standardizer ^a^	Point Estimate	90% Confidence Interval	
				Lower	Upper
Score	Cohen’s d	0.0738839	0.918	0.059	1.749
	Hedges’ correction	0.0778539	0.871 *	0.056	1.660
	Glass’s delta	0.0899359	0.754	−0.133	1.593

^a^ The denominator used in estimating the effect sizes. * Hedges’ correction uses the pooled standard deviation plus a correction factor.

**Table 12:** Independent Samples Test for Rosemary group (Control crop vs. Common crop)—FRAP method.

		Levene’s Test		*t*-Test for Equality of Means							
		F	Sig.	t	*df*	Significance		Mean Diff.	Std. Error Diff.	95% CI of the Difference	
						One-Sided *p*	Two-Sided *p*			Lower Bound	Upper Bound
Score	Equal variances assumed	0.896	0.358	−2.578	16	0.010	0.020 *	−0.2379	0.0923	−0.4336	−0.0423

* The significance level is 0.05.

**Table 13:** Concentrations of relevant active phytoconstituents and antioxidant values.

Vegetal Extract	TPC (mg/g Eq Expressed in Tannic Acid)	TPCAs (mg/g Eq Expressed in Chlorogenic Acid)	DPPH IC50 (mg/mL)	ABTS IC50 (mg/mL)	FRAP EC50 (mg/mL)
REM	236.058 ± 17.88	217.756 ± 43.13	0.0861 ± 0.0003	0.0464 ± 0.0002	0.8310 ± 0.0002
TEM	275.507 ± 22.57	227.862 ± 36.85	0.0993 ± 0.0005	0.0546 ± 0.0004	0.9795 ± 0.0001
REF	378.336 ± 38.70	277.097 ± 18.60	0.0620 ± 0.0004	0.0305 ± 0.0002	0.6206 ± 0.0001
TEF	321.678 ± 40.01	278.126 ± 45.05	0.0773 ± 0.0004	0.0419 ± 0.0009	0.8208 ± 0.0000

Total phenolic content (TPC), total phenolic acid content (TPCA), 2,2-diphenyl-1-picryl-hydrazine method (DPPH), 2,2-azinobis-3-ethylbenzotiazoline-6-sulfonic acid method (ABTS), ferric-reducing antioxidant power (FRAP).

**Table 14:** Pearson Correlation Coefficients.

		DPPH_IC_50_	ABTS_IC_50_	FRAP_EC_50_	Log_TPC_Value	TPCAs_Value
DPPH_ Method_IC_50_	Pearson Correlation	1	0.997 ***	0.978 **	−0.934 *	−0.793
	Sig. (2-tailed)		0.003	0.022	0.066	0.207
	*N*	4	4	4	4	4
ABTS_ Method_IC_50_	Pearson Correlation	0.997 ***	1	0.988 **	−0.937 *	−0.765
	Sig. (2-tailed)	0.003		0.012	0.063	0.235
	*N*	4	4	4	4	4
FRAP_ Method_EC_50_	Pearson Correlation	0.978 **	0.988 **	1	−0.887	−0.657
	Sig. (2-tailed)	0.022	0.012		0.113	0.343
	*N*	4	4	4	4	4

*** Correlation is significant at the 0.01 level (2-tailed); ** correlation is significant at the 0.05 level (2-tailed); * correlation is significant at the 0.1 level (2-tailed).

**Table 15:** Predicted binding energies, ligand efficiencies, and interacting residues after molecular docking simulations.

	BACH1				BACH2			
Compound	ΔG (kcal/mol)	LE	Hydrogen Bonds	Pi Interactions	ΔG (kcal/mol)	LE	Hydrogen Bonds	Pi Interactions
Protocatechuic acid	−5.46	0.497	Lys127	Lys100, Val103, Lys127	−5.37	0.488	Thr70, Asn72	−
Rutin	−7.22	0.168	Ser4, Ile97, Ser99, Asn102	Lys82, Ile97	−7.45	0.173	Tyr14, Asn22, Ala57, Arg117, His119	−
Caffeic acid	−5.91	0.455	Pro86, Thr93, Lys95	Pro86, Ile97	−6.03	0.464	Ala113	Phe128
Chlorogenic acid	−6.66	0.267	Gln43, Ile97, Ser99, Asn102	Val81	−7.03	0.281	Ala113, Ser124, Ser127	Phe128
Luteolin	−7.01	0.334	Pro86, Lys95, Ser99	Lyes82, Pro86, Ile97	−7.72	0.368	Cys37	Arg52(A), Arg52(B)
Kaempferol	−7.31	0.348	−	Pro86, Ile97	−7.06	0.336	His119	Cys58, Phe93, Met118, Leu121
Rosmarinic acid	−7.05	0.271	Pro86, Thr93	Val8, Ile97	−7.12	0.274	Asn22, Asp29, Lys32	Leu25, Trp63
Quercetin	−6.59	0.300	Asp26, Ala53, Asn117	His116	−7.47	0.340	Asn22, Gly26, Asn120	Trp63, His119
Isoquercitrin	−6.45	0.195	Ser7, Ile97, Glu101	Lys82, Glu85, Pro86	−7.97	0.242	Arg52, Val67, Asn72	Asp38, Arg52
Ferulic acid	−5.88	0.420	Phe123	Lys100, Lys127	−6.38	0.456	Leu36, Arg52	−
*p*-Coumaric acid	−6.17	0.514	Val8	Leu126, Lys127	−6.21	0.518	Tyr14, Cys125	Phe93, Met118, Leu121

ΔG—predicted binding energy, LE—predicted ligand efficiency.

## Data Availability

Not applicable.

## Supplementary Materials

The following supporting information can be downloaded at: https://www.mdpi.com/article/10.3390/ijms241411670/s1.
