# Supplementary material for: Implications of the Cultivation of Rosemary and Thyme (Lamiaceae) in Plant Communities for the Development of Antioxidant Therapies
Source: Int J Mol Sci. 2023 Jul 19;24(14):11670. doi: 10.3390/ijms241411670 (PMC10380601; doi:10.3390/ijms241411670)
Supplement: Supplementary file 1 [file ijms-24-11670-s001.zip › ijms-2521010-supplementary.pdf]

Article

Implications of the Cultivation of Rosemary and Thyme (*Lamiaceae*) in Plant Communities for the Development of Antioxidant Therapies

S.1. Materials and methods

S1.1. Standard curves

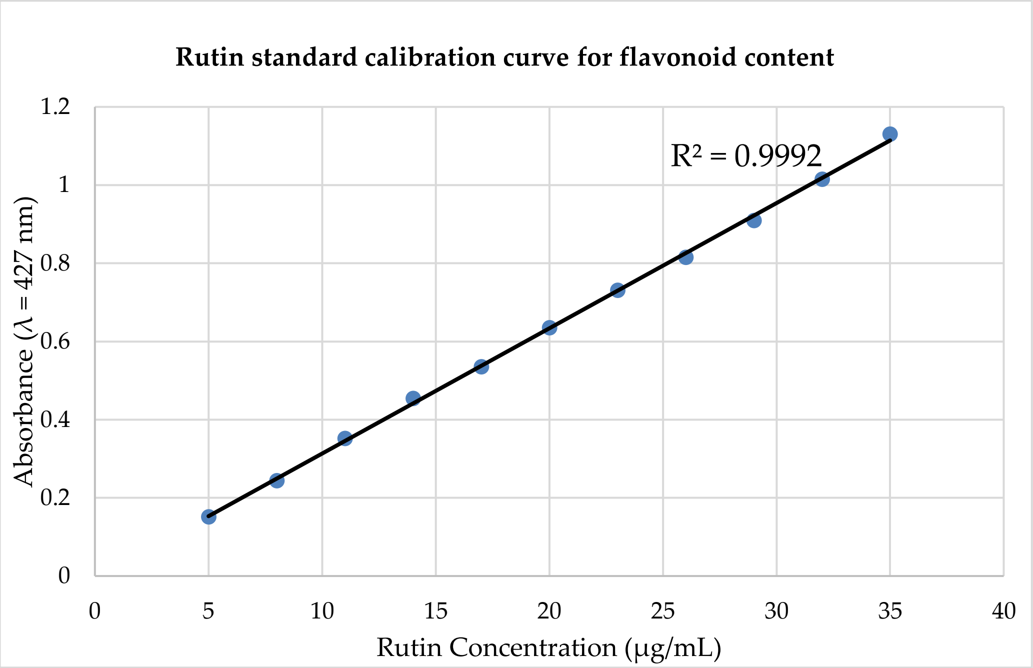

Figure S1. Standard curve of rutin (TFL)

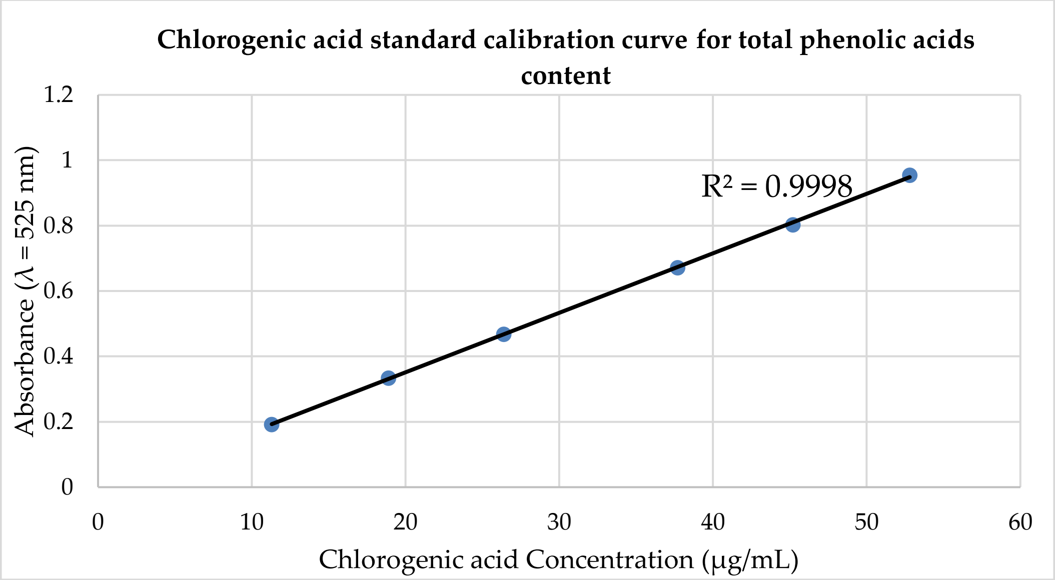

Figure S2. Standard curve of chlorogenic acid (TPCAs)

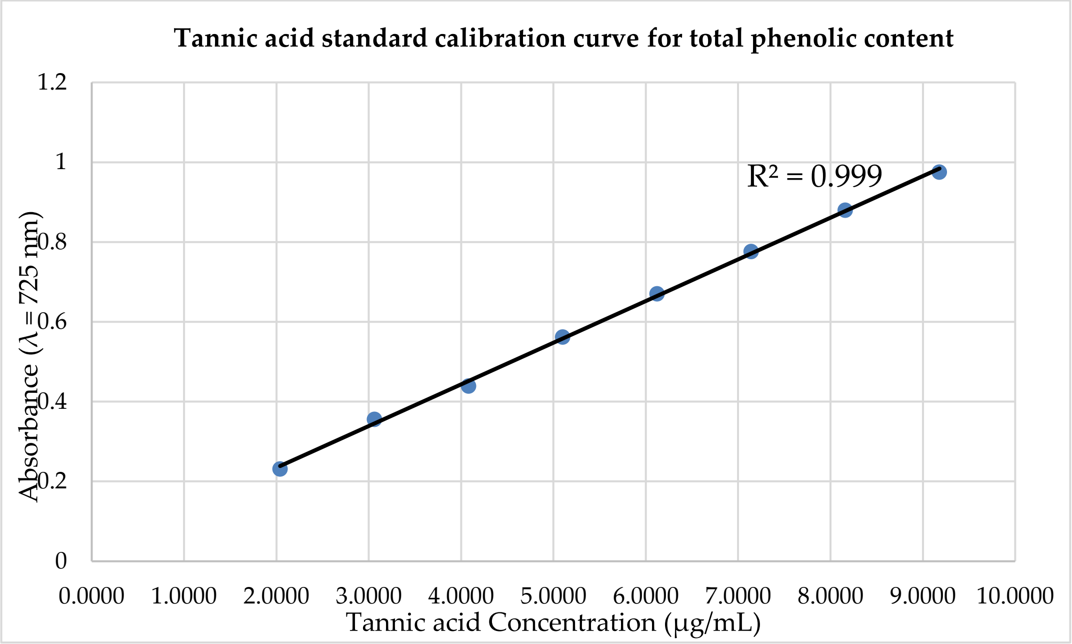

Figure S3. Standard curve of tannic acid (TPC)

S.1.2. UHPLC methodology

The method used to prepare injection solutions was as follows: 1 mg/mL stock solutions of each standard compound were obtained in methanol and kept refrigerated at 4°C until use. The concentration of each level of the calibration curves are presented in Table S1. The solutions were obtained by diluting in the first line of the mobile phase gradient (methanol-water, 9:1). The samples were dissolved in the same mixture of methanol-water (9:1) and filtered through 0.2 μm syringe filters (Acrodisc MS Syringe Filters WWPTFE Membrane) prior to injection.

Table S1. Calibration curve concentration by level (expressed in μg/g) and purity (%).

Level	PRO 99%	CHL 98%	CAF 99%	COU 99%	FER 98%	RUT 94%	ISO 87%	ROS 96%	LUT 99%	QUE 95%	KAE 99%
1	5.05	4.90	4.95	5.00	4.90	4.70	2.18	4.80	1.98	4.75	0.20
2	10.10	9.80	9.90	10.00	9.80	9.40	4.35	9.60	3.96	9.50	0.40
3	20.20	19.60	19.80	20.00	19.60	18.80	8.70	19.20	7.92	19.00	0.79
4	50.49	49.00	49.50	50.00	49.00	47.00	21.75	48.00	19.80	47.50	1.98

PRO – Protocatechuic acid, RUT – Rutin, CAF – Caffeic acid, CHL – Chlorogenic acid, LUT – Luteolin, KAE – Kaempferol, ROS – Rosmarinic acid, QUE – Quercetin, ISO – Isoquercitrin, FER – Ferulic acid, COU – *p*-Coumaric acid.

LC conditions

All separations were performed on the ACQUITY Arc System equipped with an ACQUITY QDa Detector. Empower 3 Software was used for data acquisition and processing. The Column used is a CORTECS C_{18}, 4.6×50 mm, 2.7 μm. Ammonium formate 10 mmol (solvent A) and acetonitrile (solvent B) with a flow rate of 0.5 mL/min. The volume injected was 5 μL, and the analysis time was 21 min. The gradient conditions were: 0 min 8% B, 8 min 20% B, 16 min 27% B, 19 min 60% B, 20 min 60% B, and 21 min 8% B.

MS conditions

Ionization mode ESI-

All compounds were satisfactorily separated. Chromatogram and retention times are presented below (Figure S4 and Table S2).

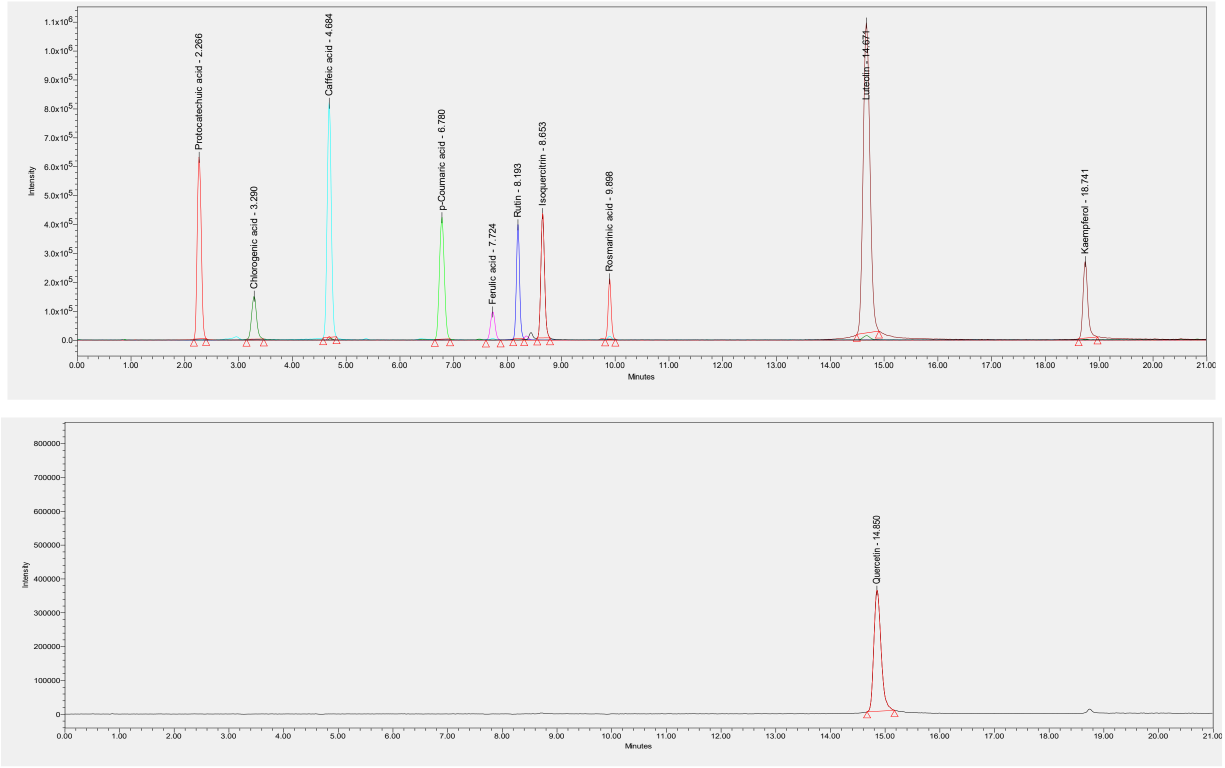

Figure S4. Standard chromatogram.

Table S2. Retention times.

Compound name	Retention time (Rt-Min)
Protocatechuic acid	2.266
Chlorogenic acid	3.290
Caffeic acid	4.684
p-Coumaric acid	6.780
Ferulic acid	7.724
Rutin	8.193
Isoquercitrin	8.653
Rosmarinic acid	9.898
Luteolin	14.671
Quercetin	14.850
Kaempferol	18.741

S.1.3. Antioxidant activity

S.1.3.1 Ascorbic acid standard and correlation with the antioxidant activity

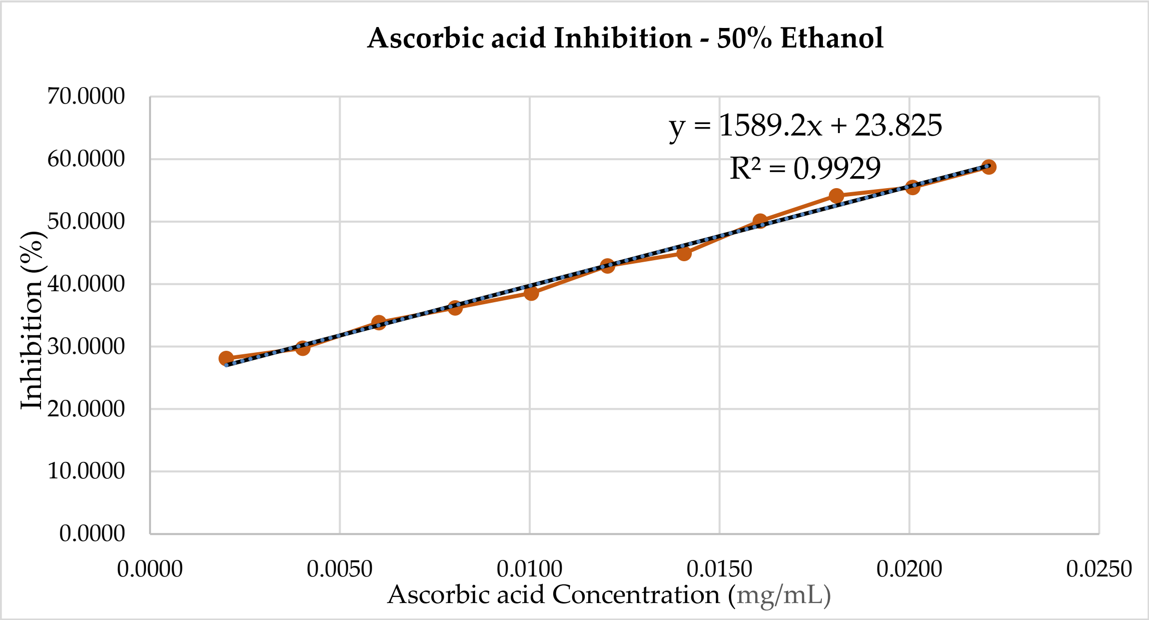

Figure S5. Calibration curve for ascorbic acid (vitamin C) - Antioxidant action in 50% ethanol.

S1.3.2. Trolox standard and correlation with the antioxidant activity

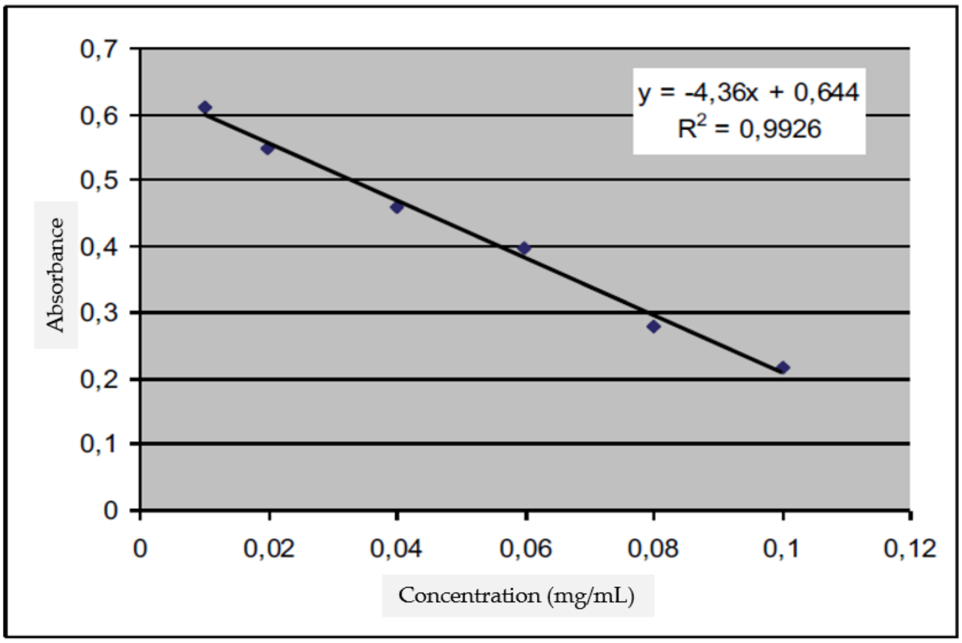

Figure S6. Calibration curve for Trolox - Antioxidant action in 50% ethanol.

S1.3.3. Ferrous sulfate standard and correlation with the antioxidant activity

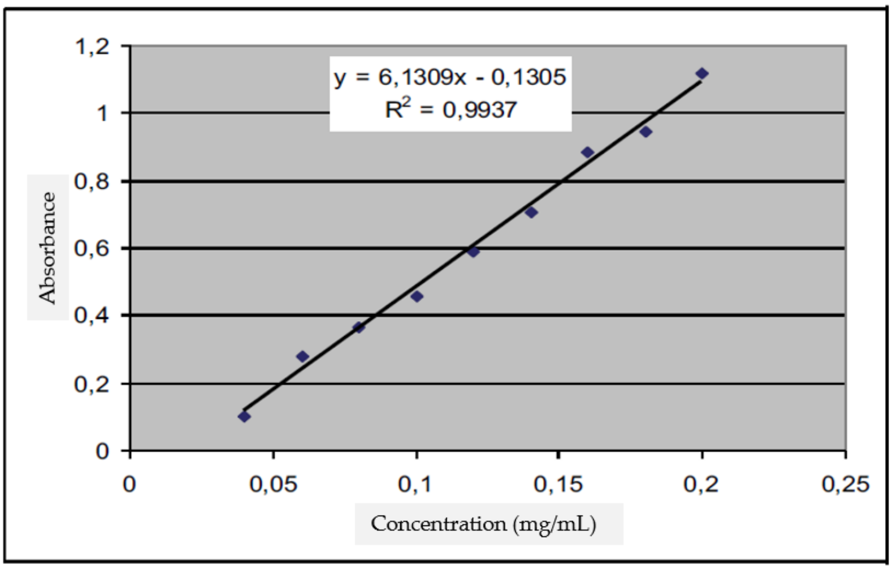

Figure S7. Calibration curve for ferrous sulfate - Antioxidant action in 50% ethanol.

S.2. Results

S2.1. UHPLC MS mass spectra

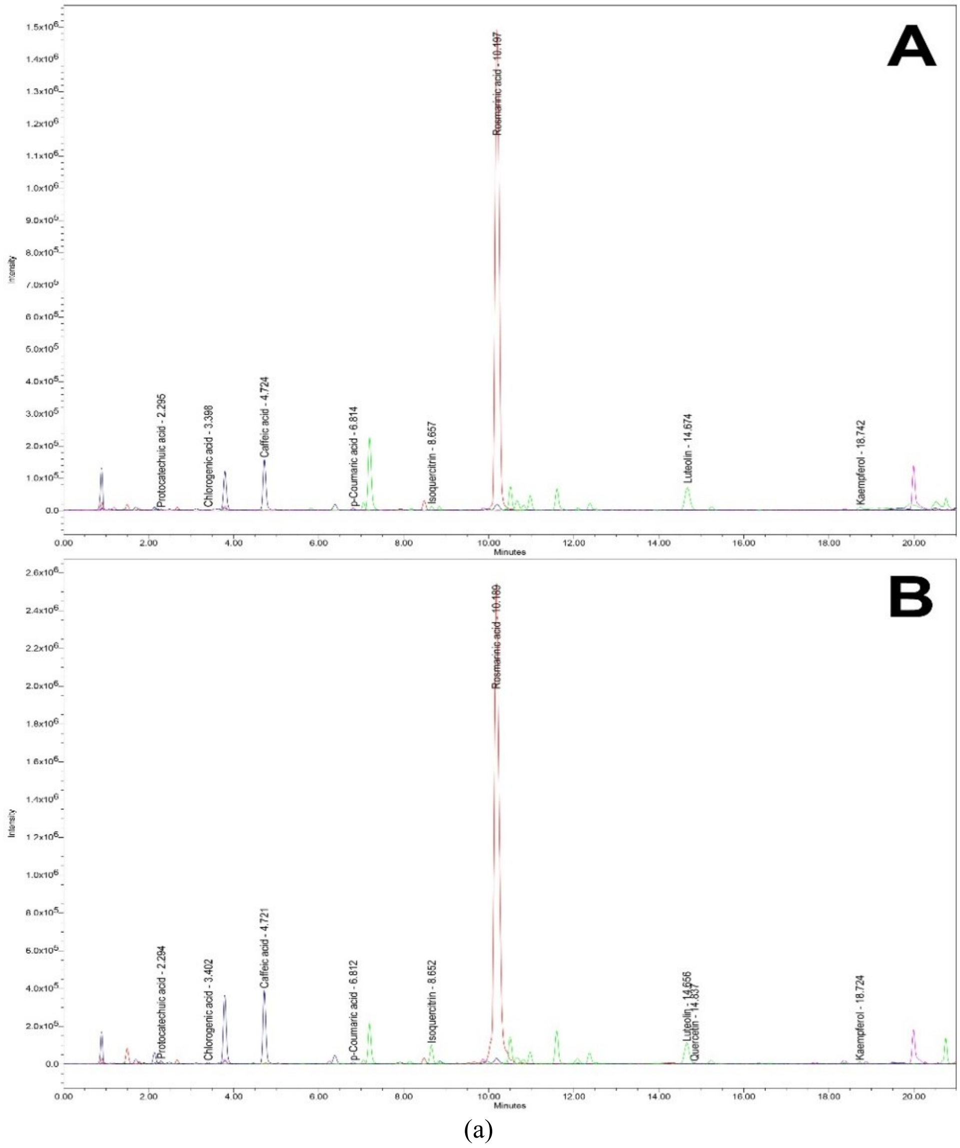

(a)

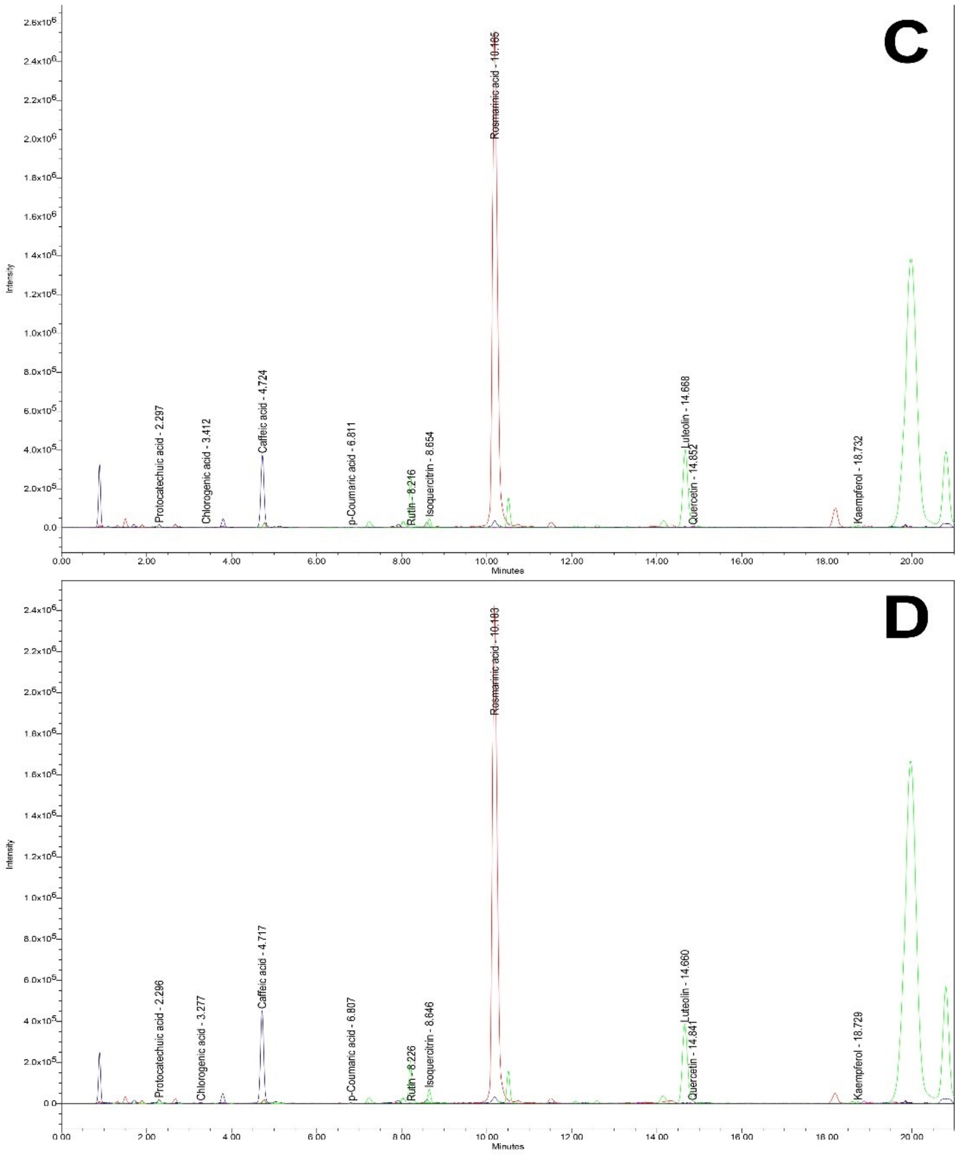

(b)

Figure S8. (**a**) *Rosmarinus* extract, entire spectra, ; (A)—REM and (B)—REF; (b) *Thymus* extract, entire spectra, ESI−; (C)—TEM and (D)—TEF, SIR chromatogram. Legend: PRO—Protocatechuic acid, RUT—Rutin, CAF—Caffeic acid, CHL—Chlorogenic acid, LUT—Luteolin, KAE—Kaempferol, ROS—Rosmarinic acid, QUE—Quercetin, ISO—Isoquercitrin, FER—Ferulic acid, COU—*p*-Coumaric acid

S2.2. FT-ICR MS mass spectra

S2.2.1.**ESI+ ionisation**

Rosmarinus officinalis L.
Green color chromatogram – REM (rosemary extract from control crop) - a
Violet color chromatogram – REF (rosemary extract from common crop) - b
Grey color chromatogram – theoretical peak obtained by the computer program - c

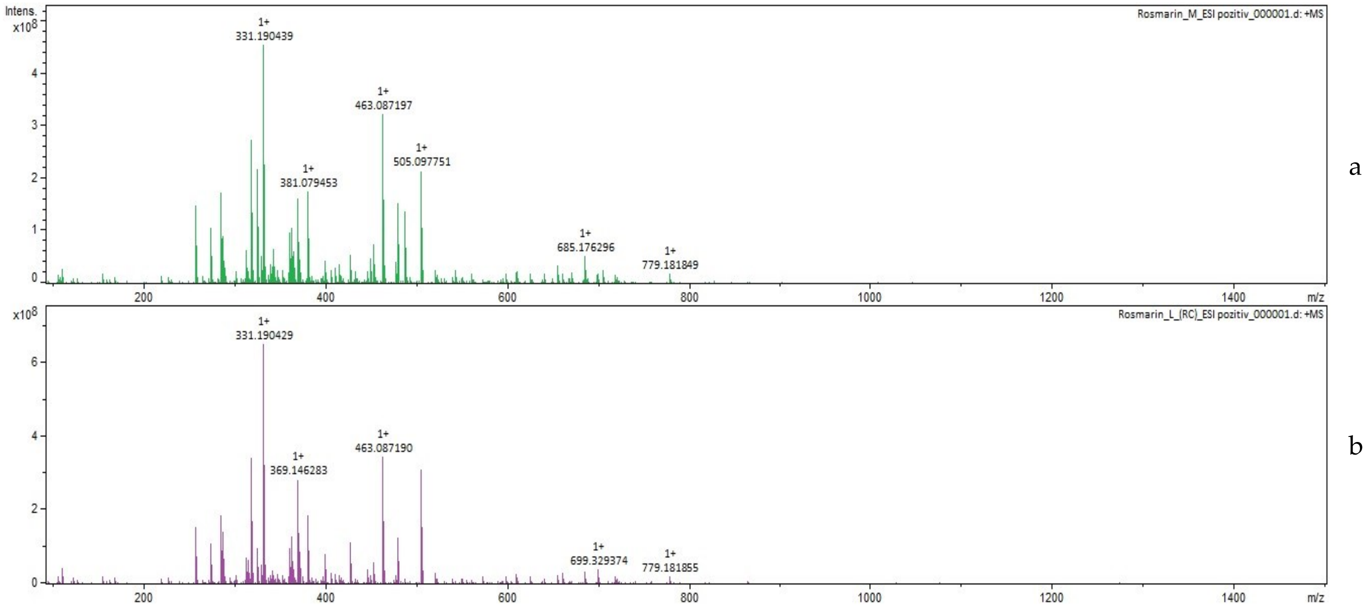

Figure S9. (a) and (b) – entire mass spectra of rosemary dry extract obtained on positive ionization.

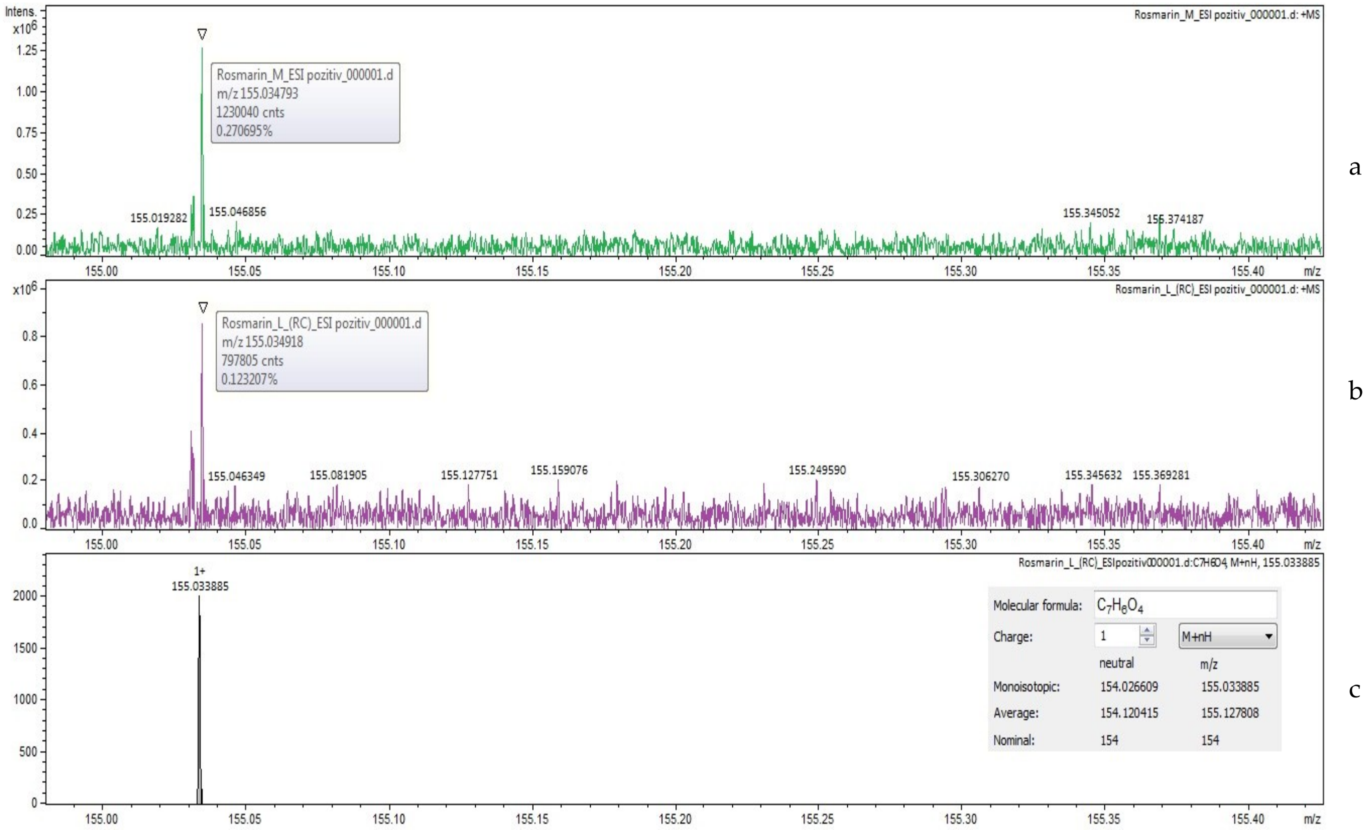

Figure S10. Protocatechuic acid ($C_7H_6O_4$) – m/z is 155.03, ESI+.

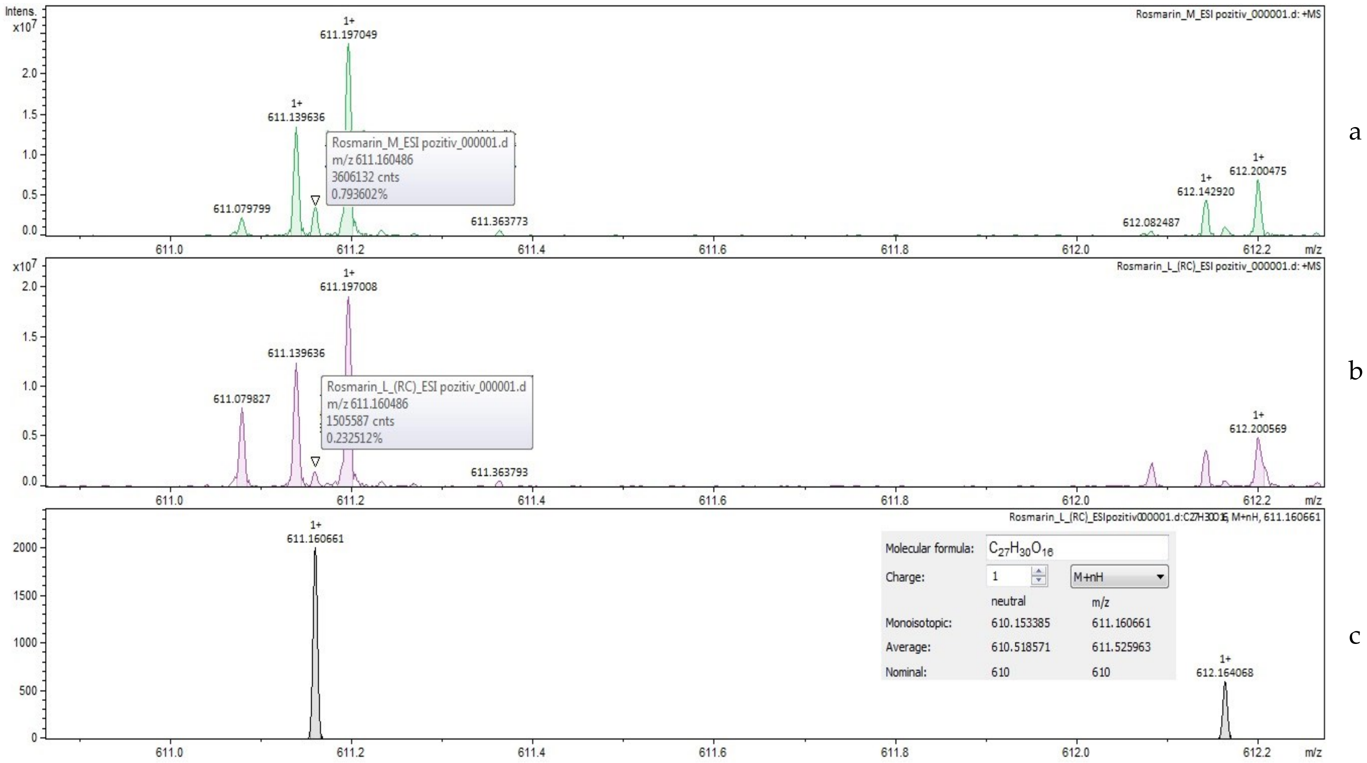

Figure S11. Rutin ($C_{27}H_{30}O_{16}$) – m/z is 611.16, ESI+.

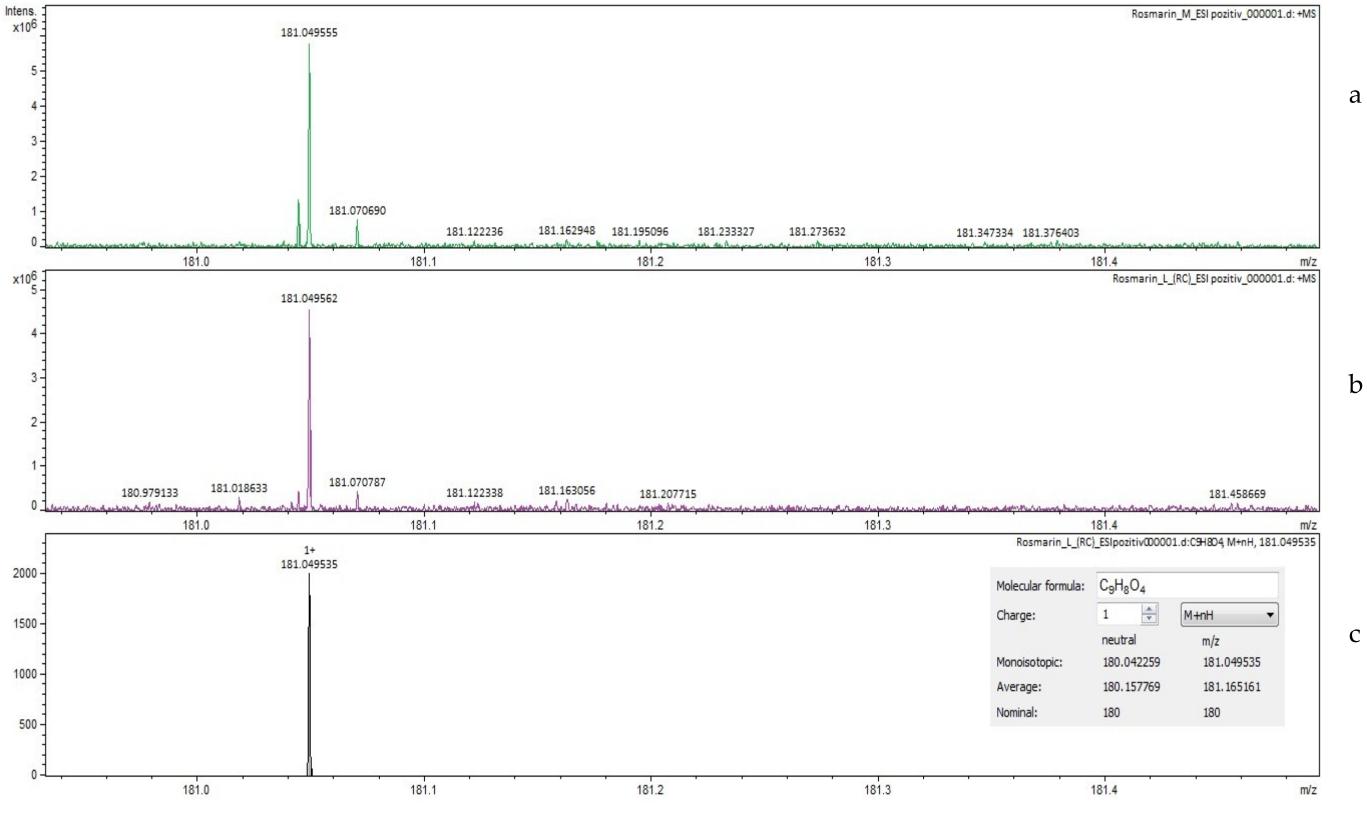

Figure S12. Caffeic acid ($C_9H_8O_4$) – m/z is 181.04, ESI+.

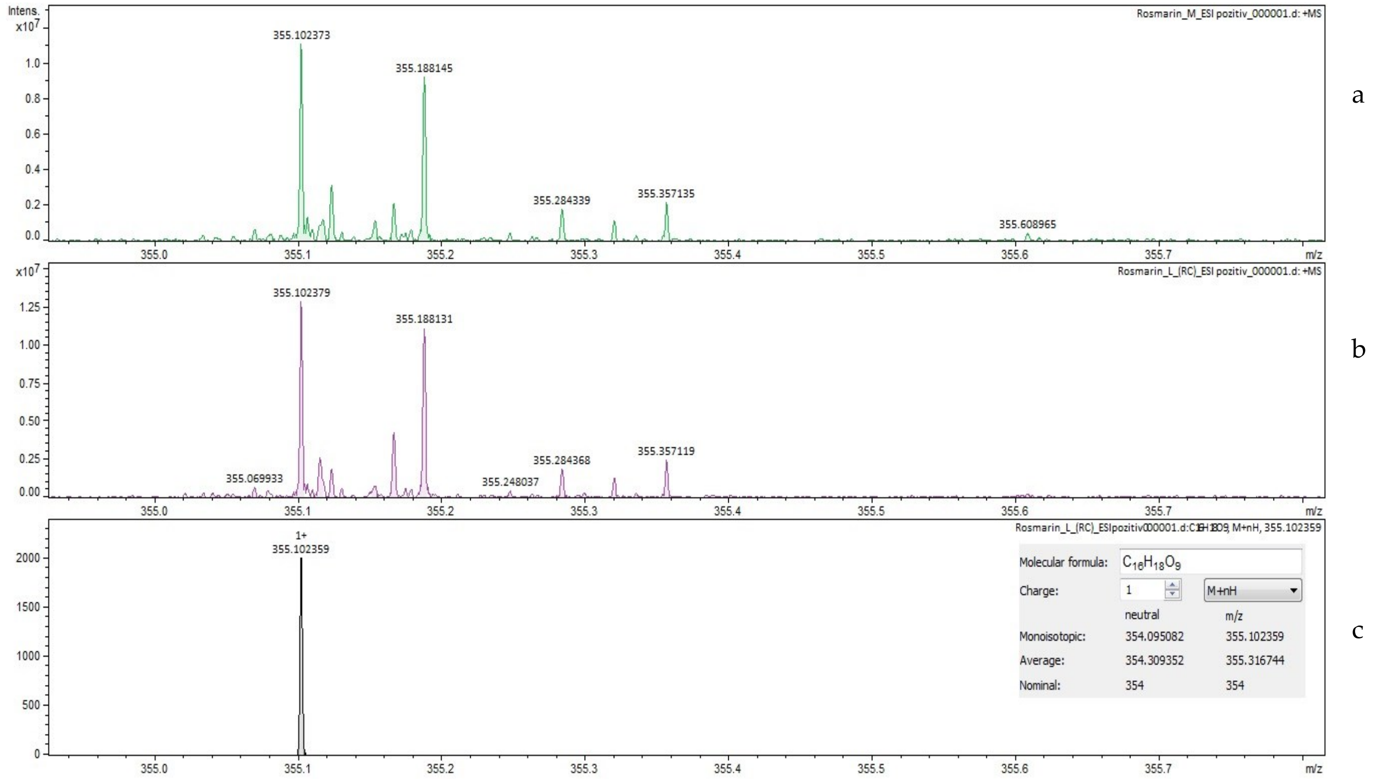

Figure S13. Chlorogenic acid ($C_{16}H_{18}O_9$) – m/z is 355.10, ESI+.

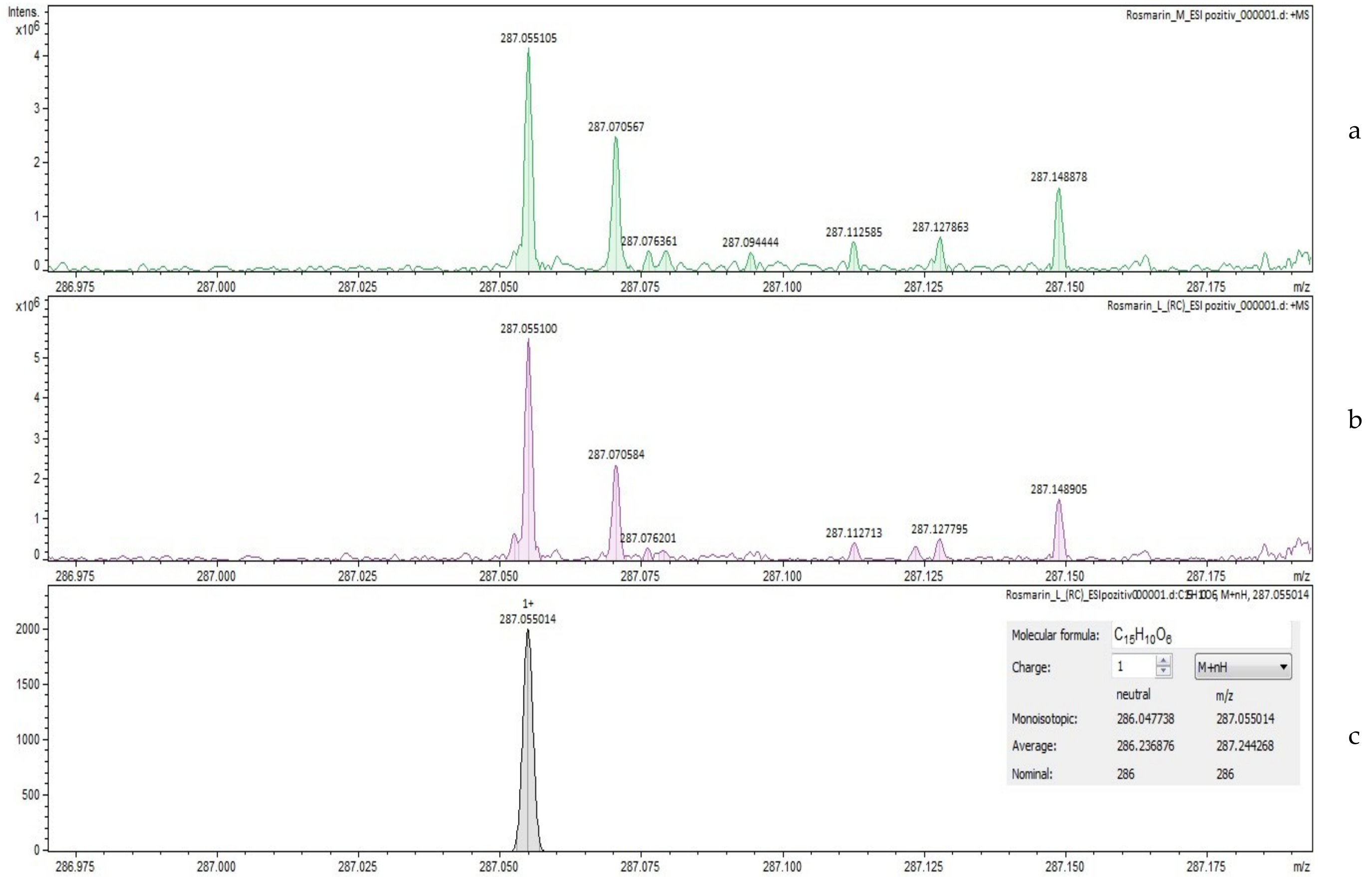

Figure S14. Luteolin + Kaempferol ($C_{15}H_{10}O_6$) – m/z is 287.05, ESI+.

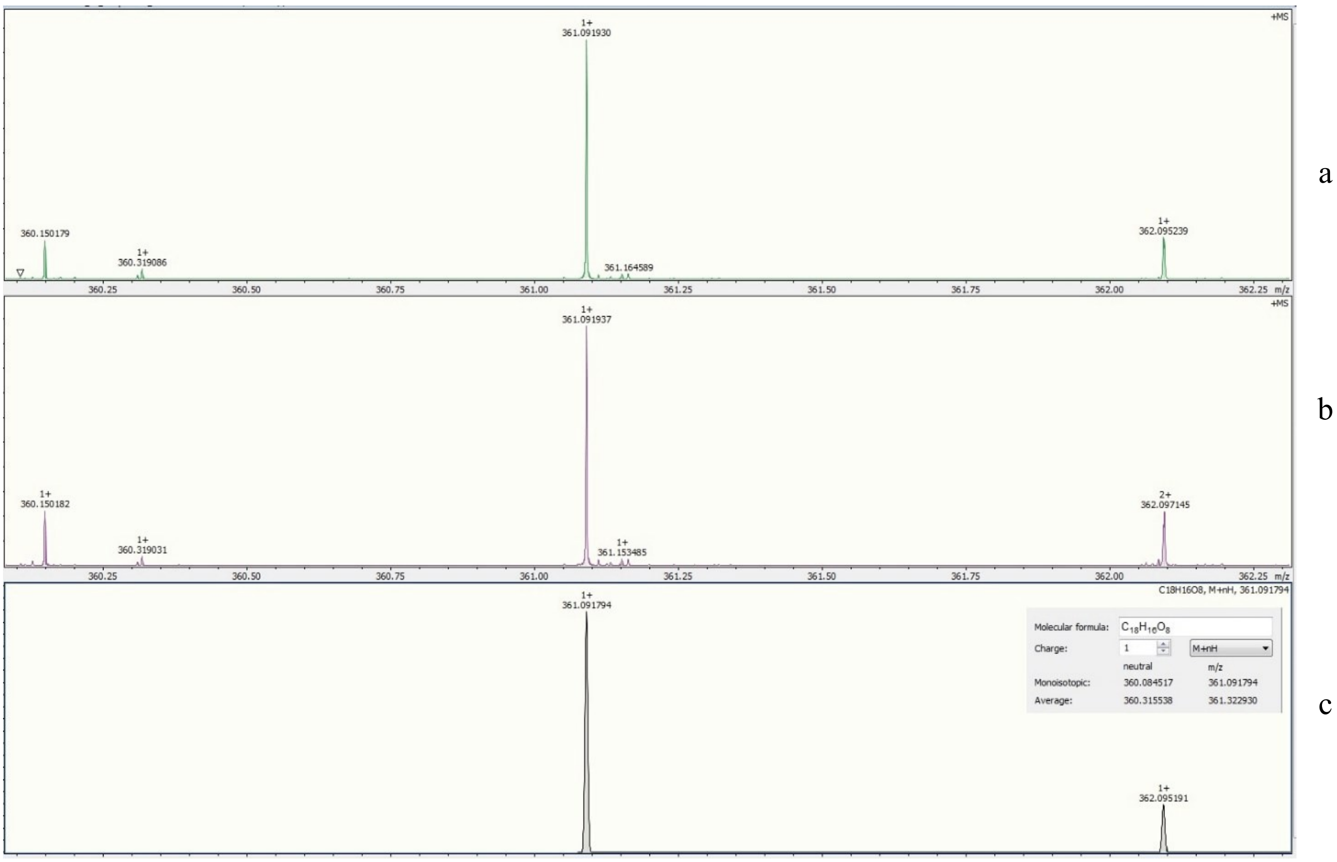

Figure S15. Rosmarinic acid ($C_{18}H_{16}O_8$) – m/z is 361.09, ESI+.

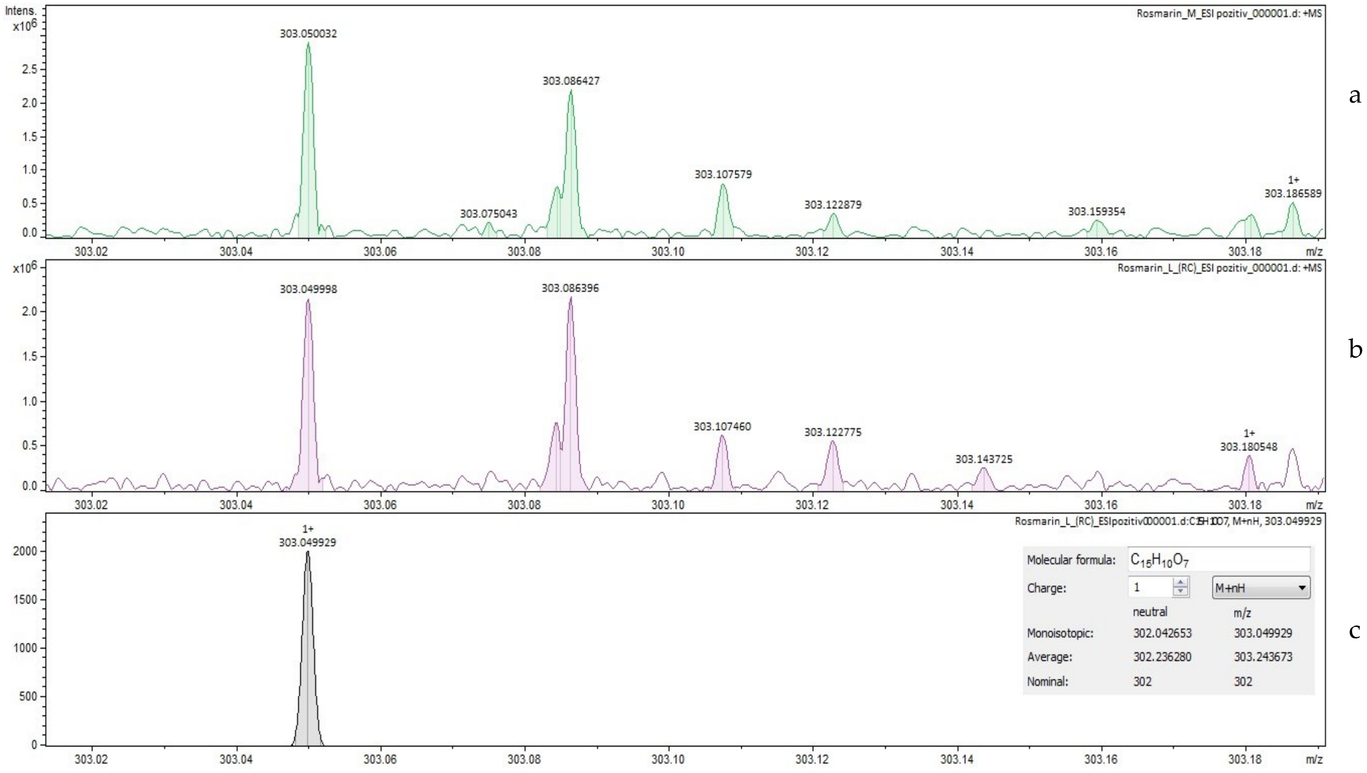

Figure S16. Quercetin ($C_{15}H_{10}O_7$) – m/z is 303.04, ESI+.

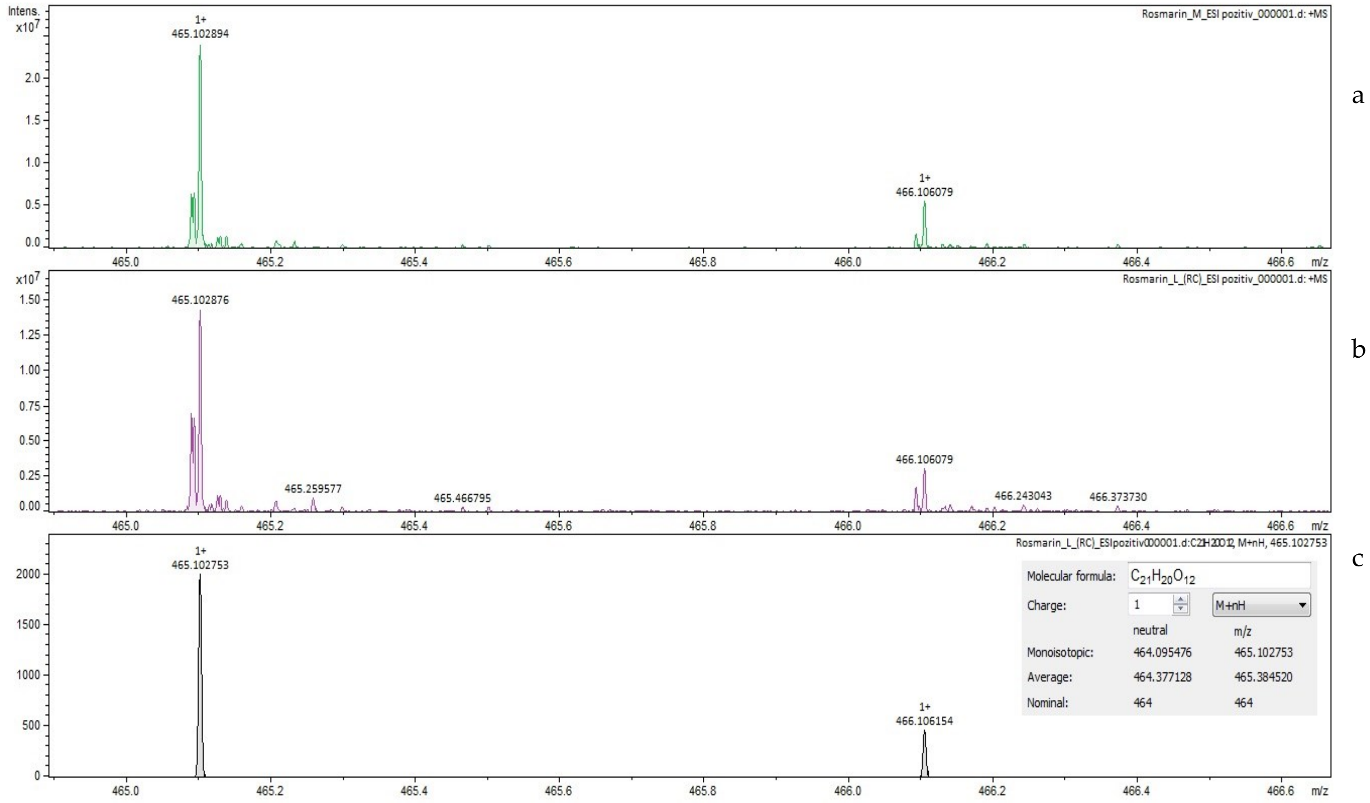

Figure S17. Isoquercitrin ($C_{21}H_{20}O_{12}$) – m/z is 465.10, ESI+.

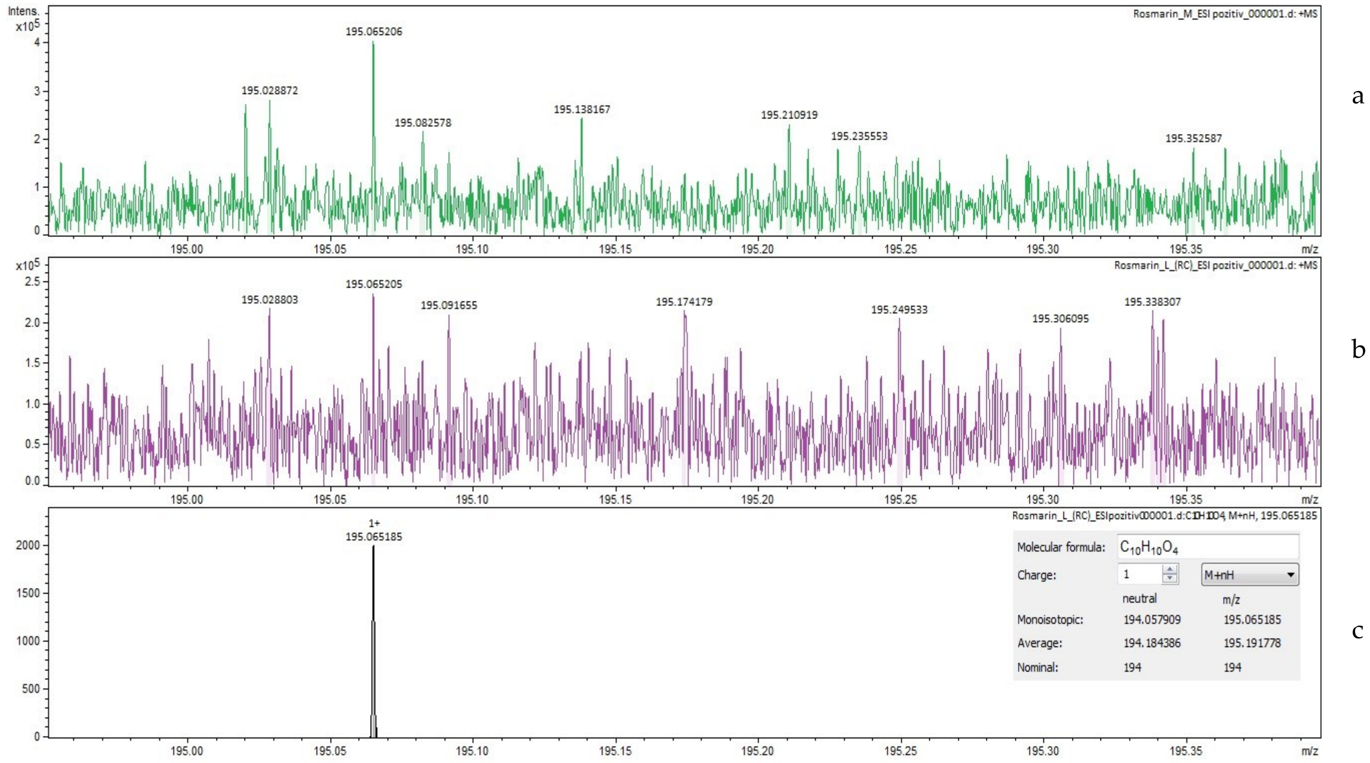

Figure S18. Ferulic acid ($C_{15}H_{10}O_{6}$) – m/z is 195.07, ESI+.

Thymus vulgaris L.
Blue color chromatogram – TEM (thyme extract from control crop) - a
Green color chromatogram – TEF (thyme extract from common crop) - b
Grey color chromatogram – theoretical peak obtained by the computer program - c

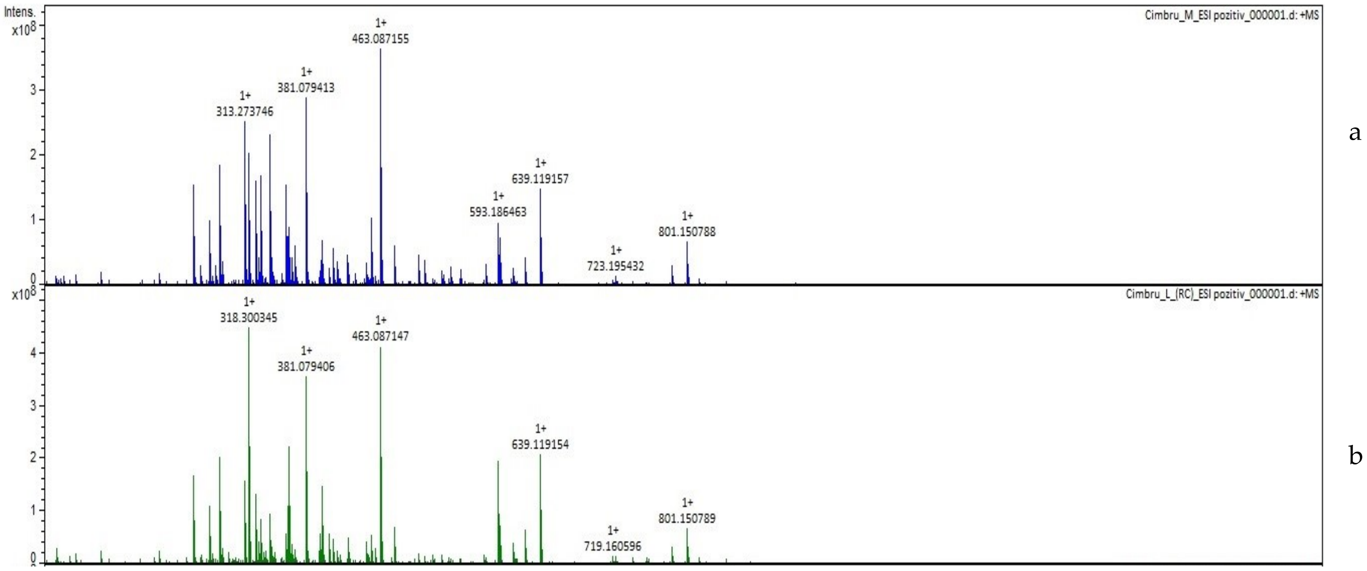

Figure S19. (a) and (b) – entire mass spectra of thyme dry extract obtained on positive ionization.

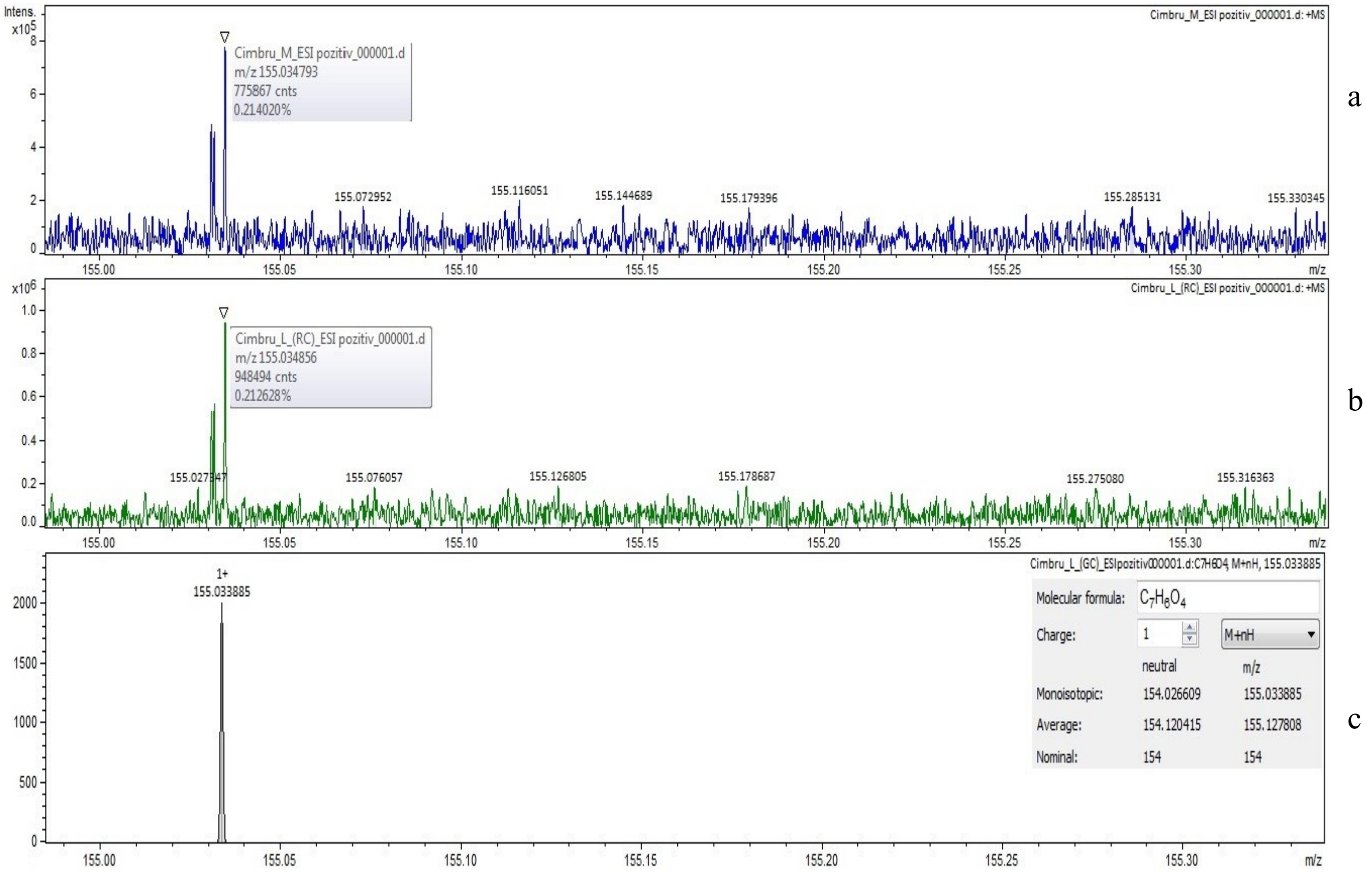

Figure S20. Protocatechuic acid ($C_7H_6O_4$) – m/z is 155.03, ESI+.

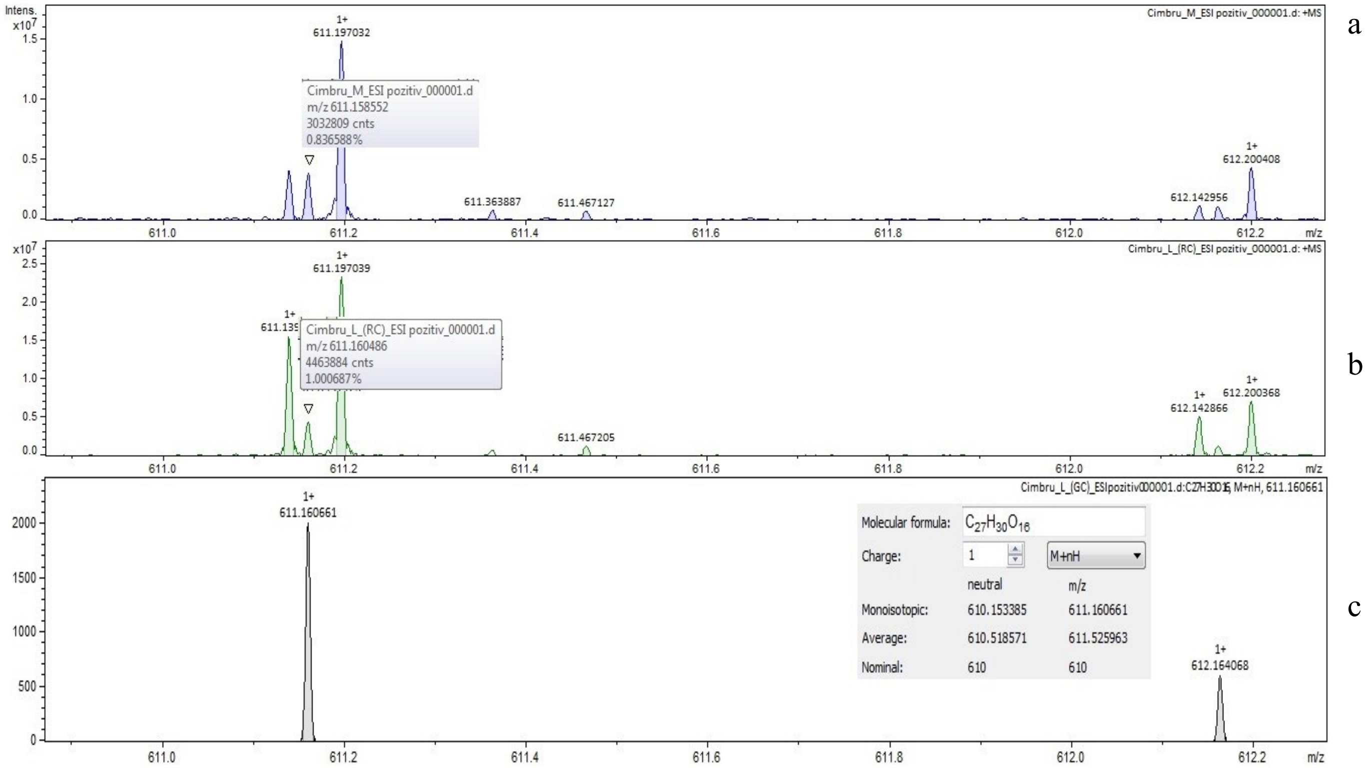

Figure S21. Rutin ($C_{27}H_{30}O_{16}$) – m/z is 611.16, ESI+.

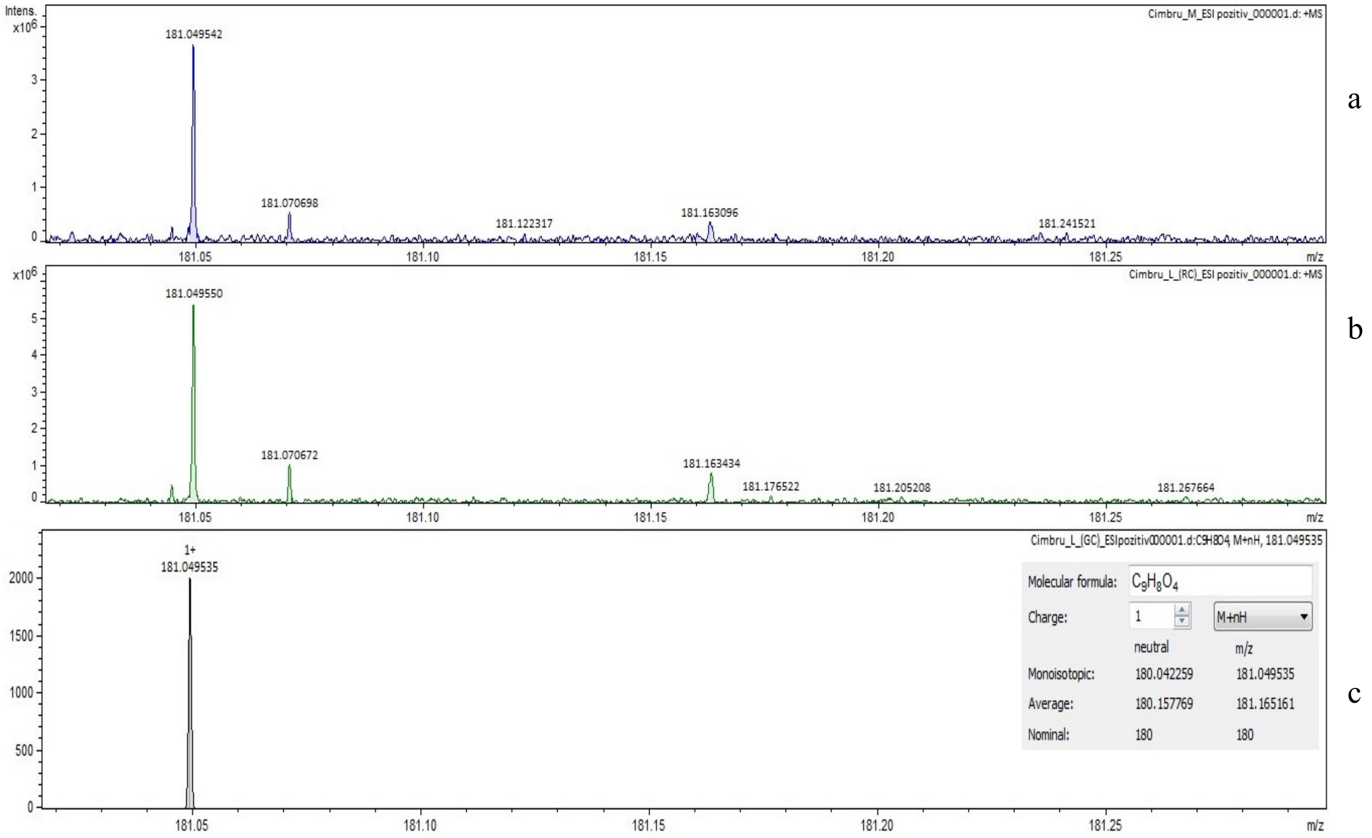

Figure S22. Caffeic acid ($C_9H_8O_4$) – m/z is 181.04, ESI+.

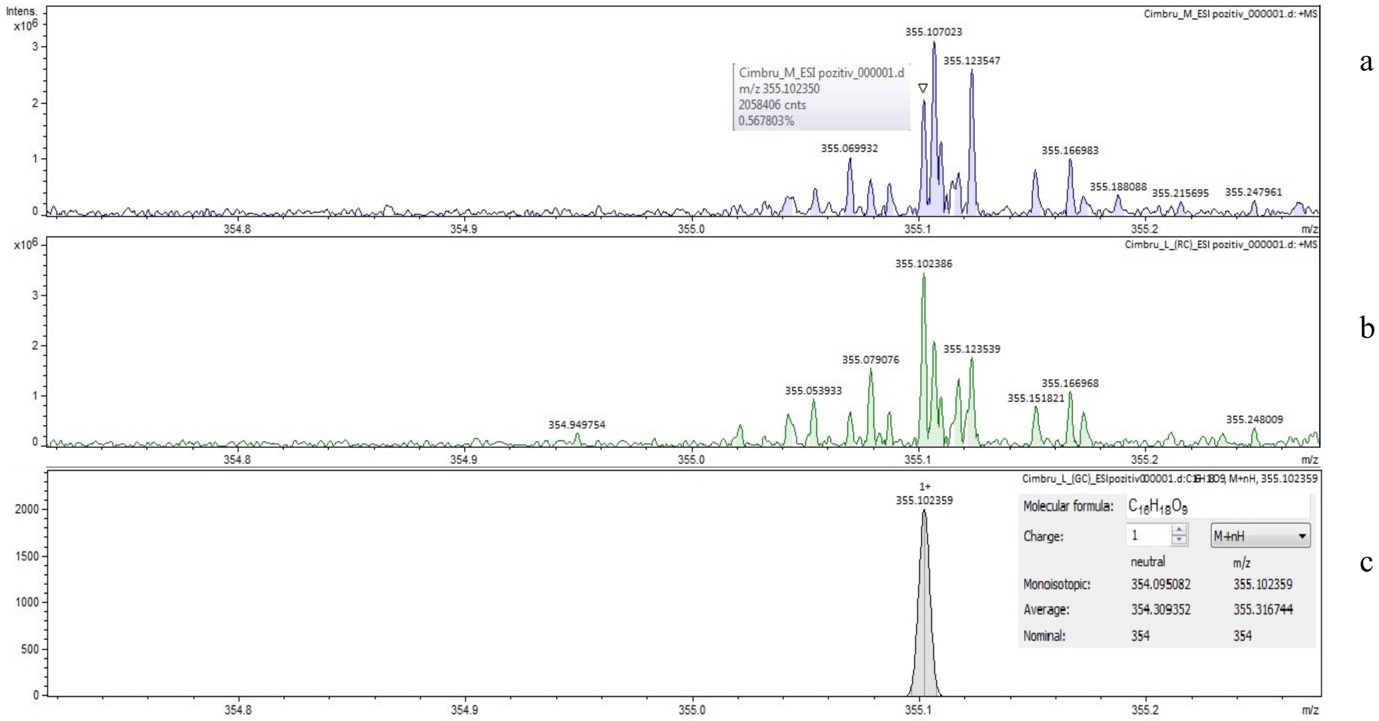

Figure S23. Chlorogenic acid ($C_{16}H_{18}O_9$) – m/z is 355.10, ESI+.

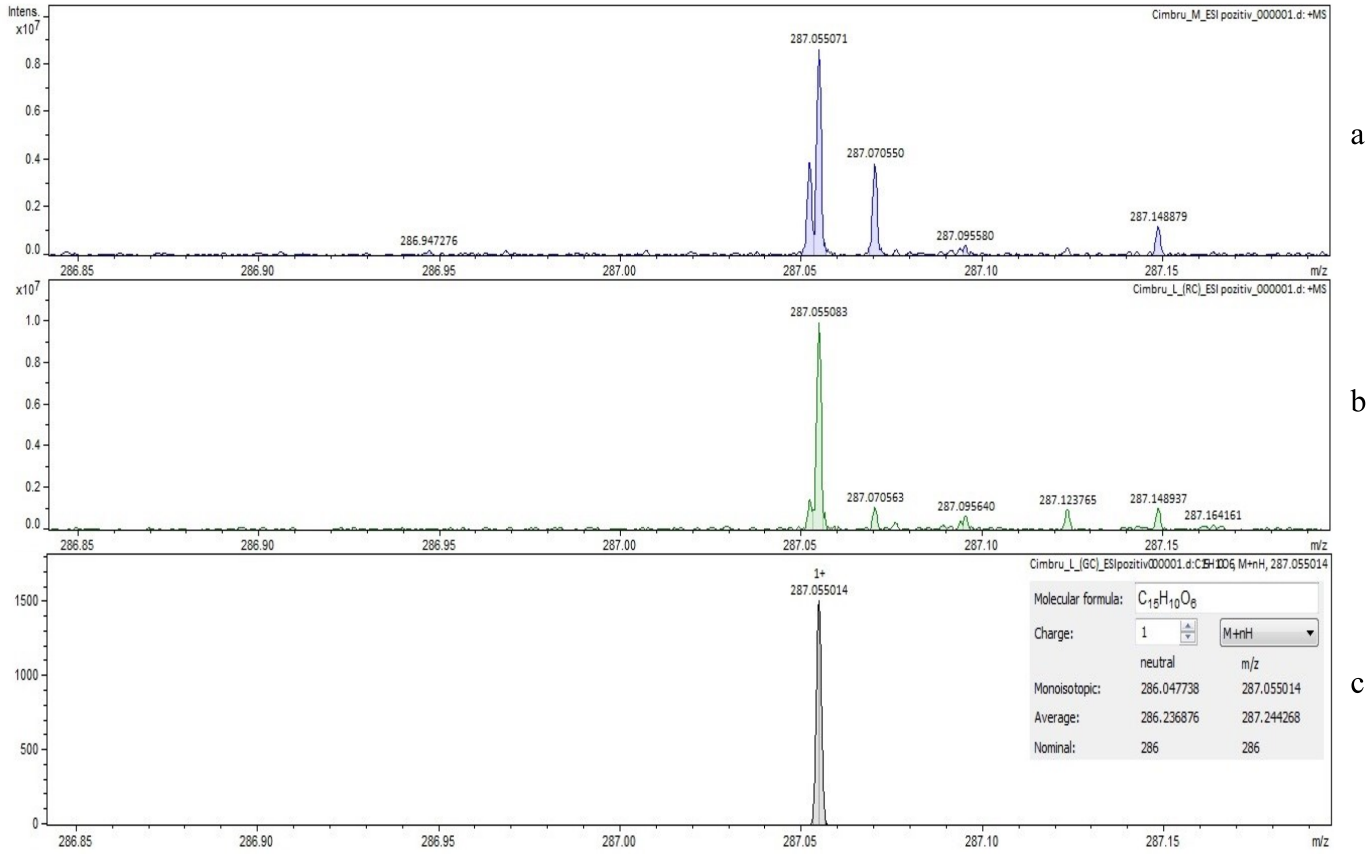

Figure S24. Luteolin + Kaempferol ($C_{15}H_{10}O_6$) – m/z is 287.05, ESI+.

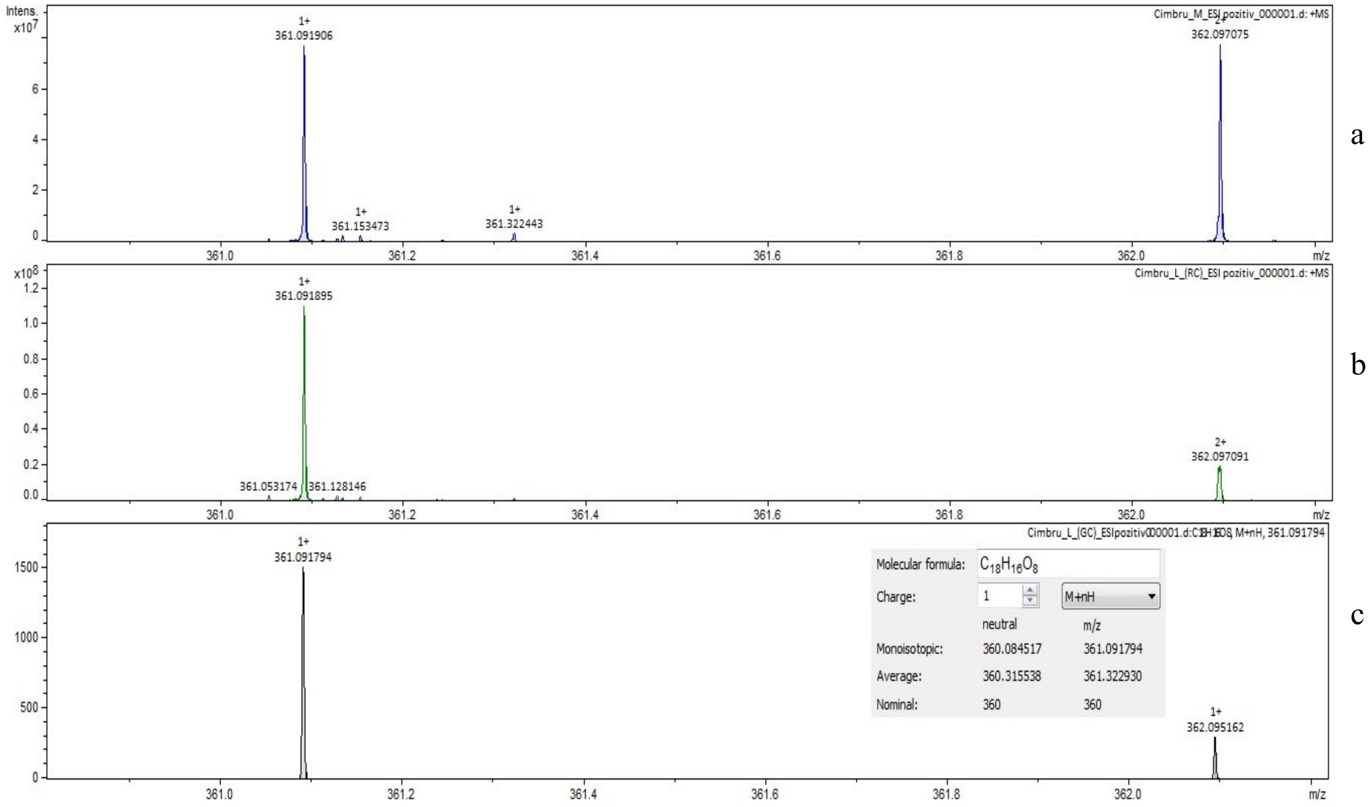

Figure S25. Rosmarinic acid ($C_{18}H_{16}O_8$) – m/z is 361.09, ESI+.

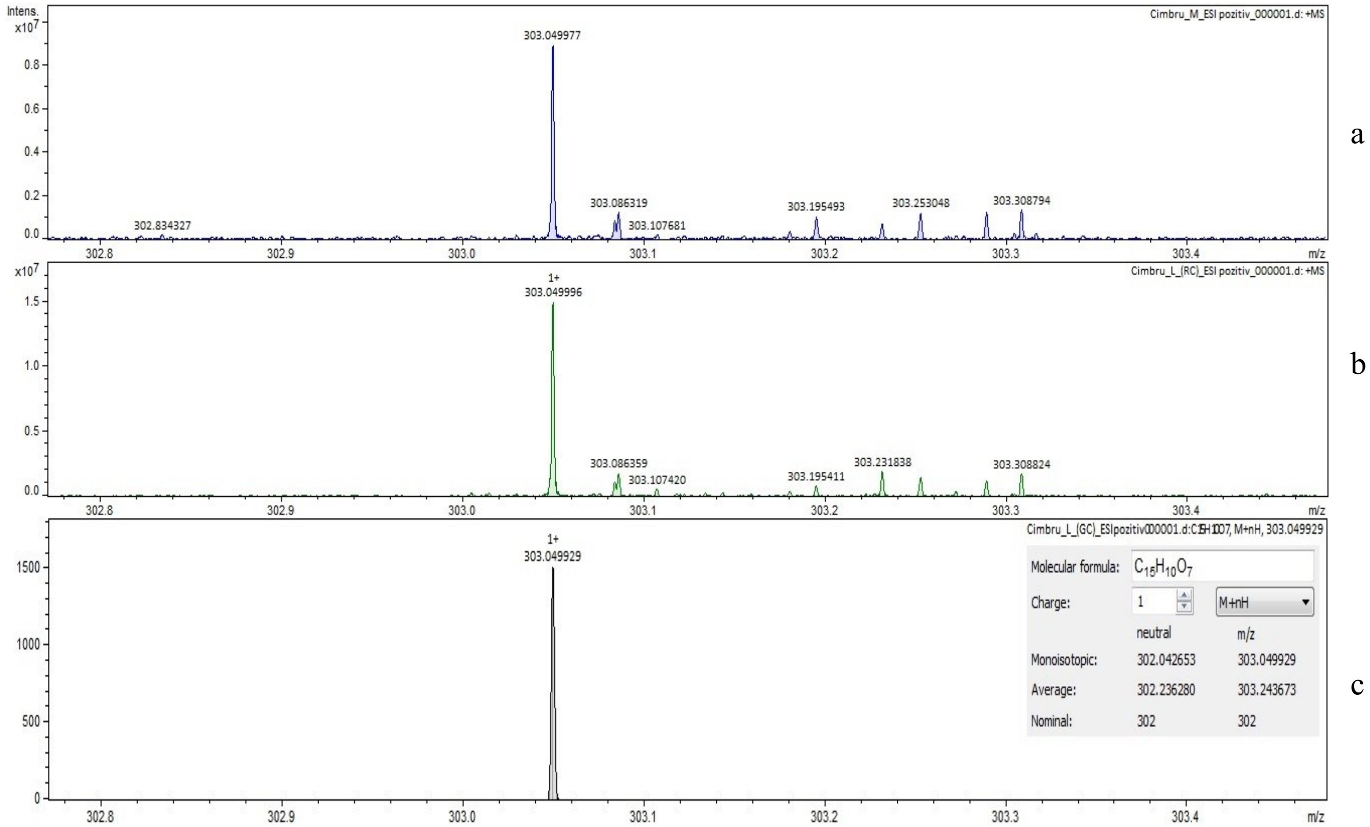

Figure S26. Quercetin ($C_{15}H_{10}O_7$) – m/z is 303.04, ESI+.

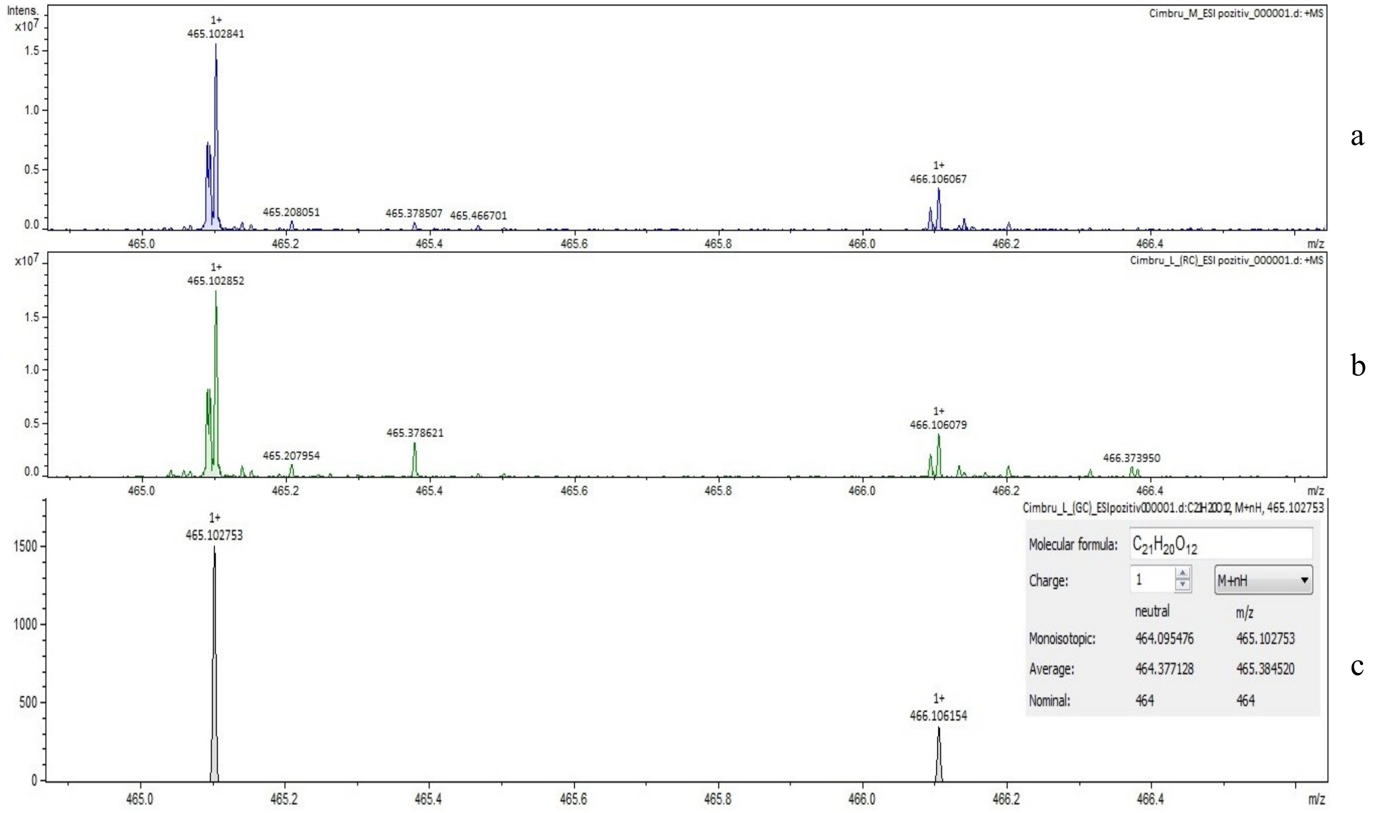

Figure S27. Isoquercitrin ($C_{21}H_{20}O_{12}$) – m/z is 465.10, ESI+.

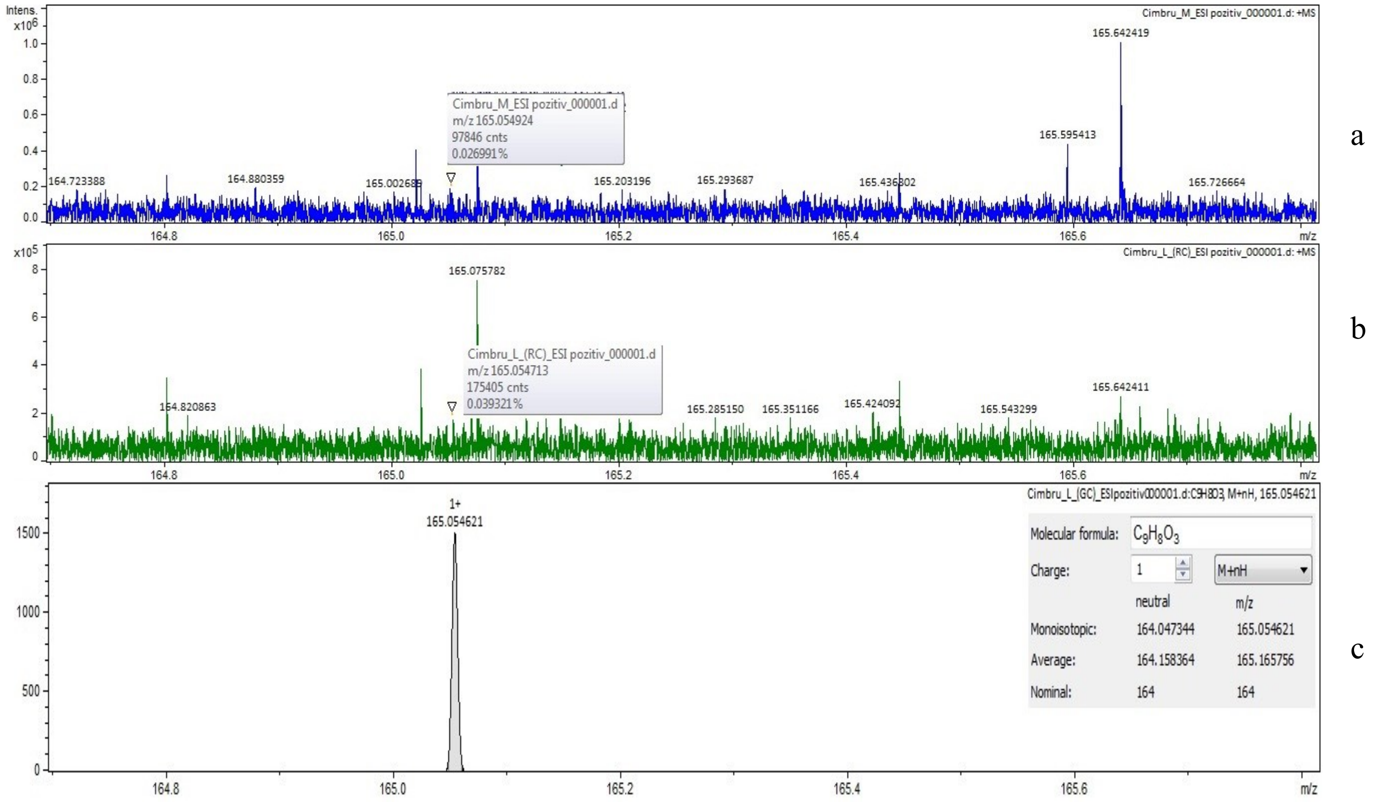

Figure S28. *p*-Coumaric acid ($C_9H_8O_3$) – m/z is 165.05, ESI+.

S2.2.2.**ESI- ionisation**

Rosmarinus officinalis L.
Green color chromatogram – REM (rosemary extract from control crop) - a
Violet color chromatogram – REF (rosemary extract from common crop) - b
Grey color chromatogram – theoretical peak obtained by the computer program - c

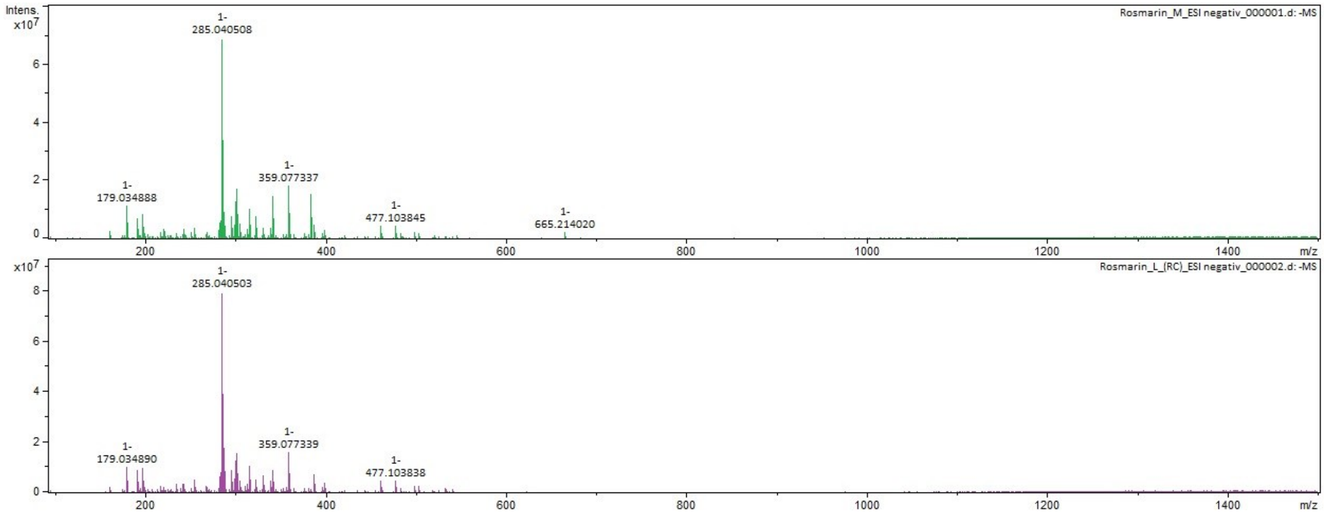

a

b

Figure S29. (a) and (b) – entire mass spectra of rosemary dry extract obtained on negative ionization.

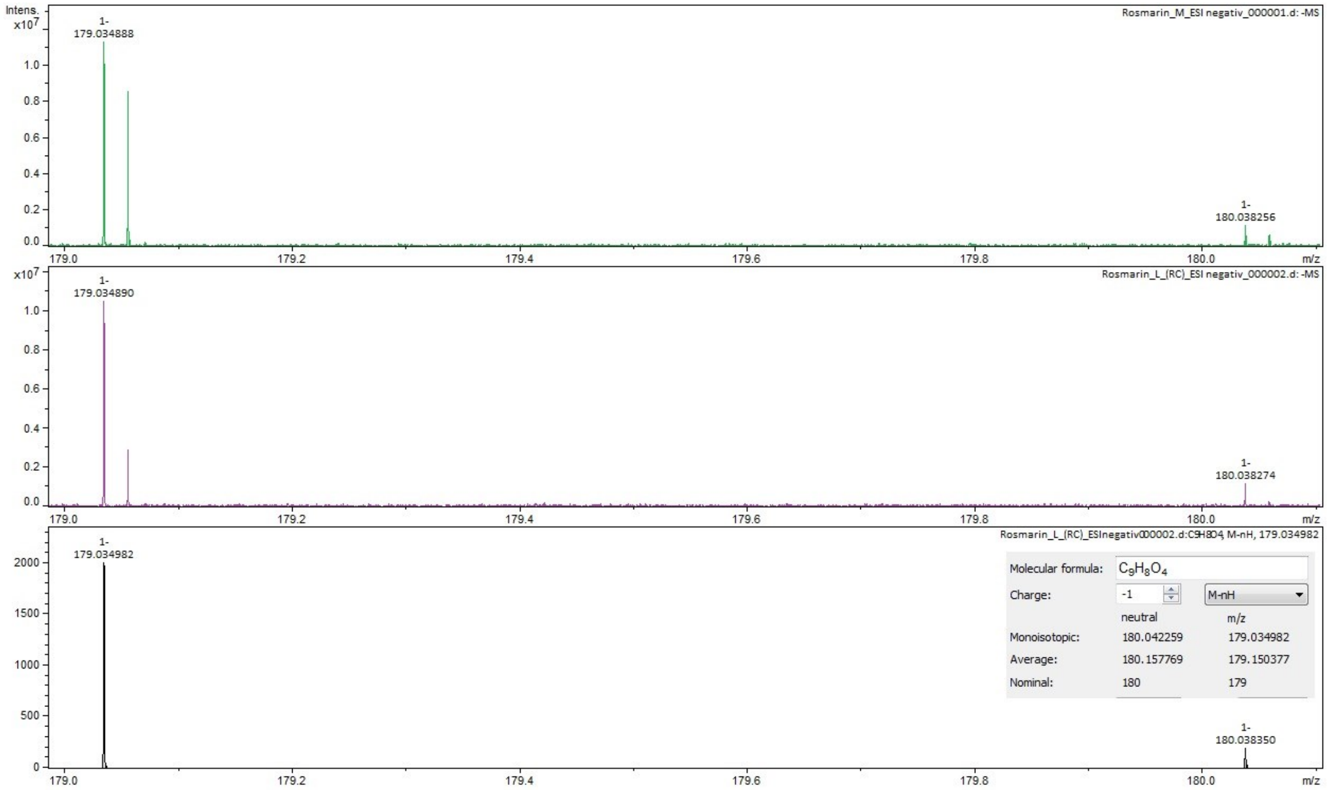

a

b

c

Figure S30. Caffeic acid ($C_9H_8O_4$)– m/z is 179.03, ESI-.

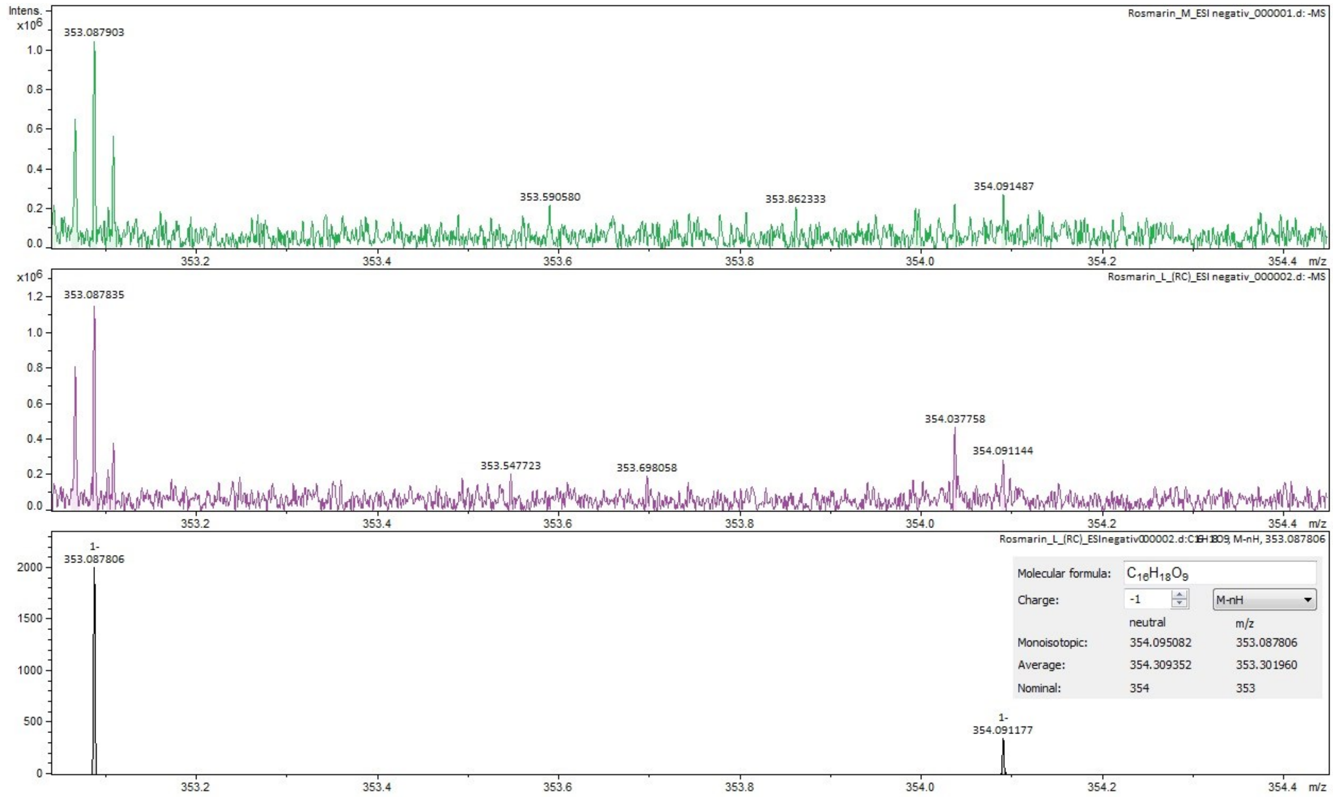

a

b

c

Figure S31. Chlorogenic acid ($C_{16}H_{18}O_9$) – m/z is 353.08, ESI –.

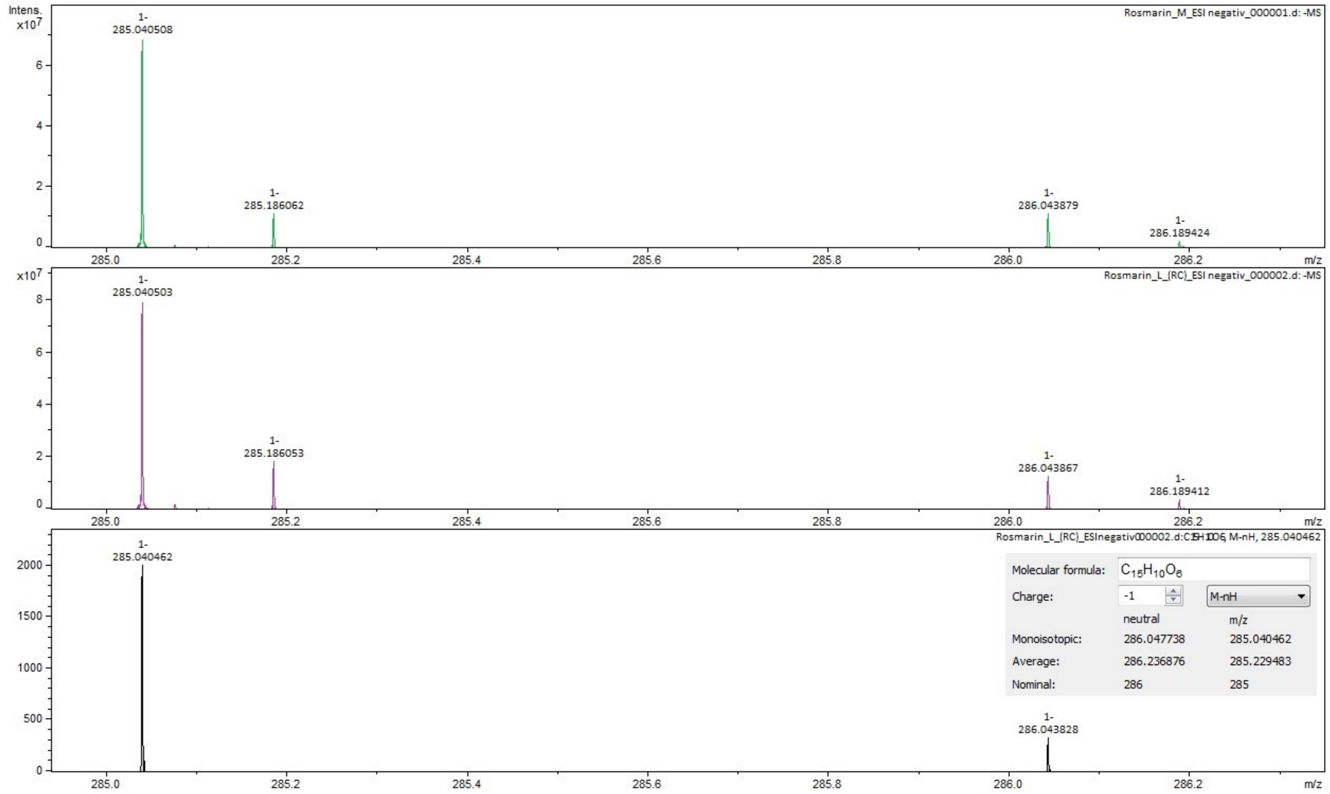

a

b

c

Figure S32. Luteolin + Kaempferol ($C_{15}H_{10}O_6$) – m/z is 285.04, ESI-.

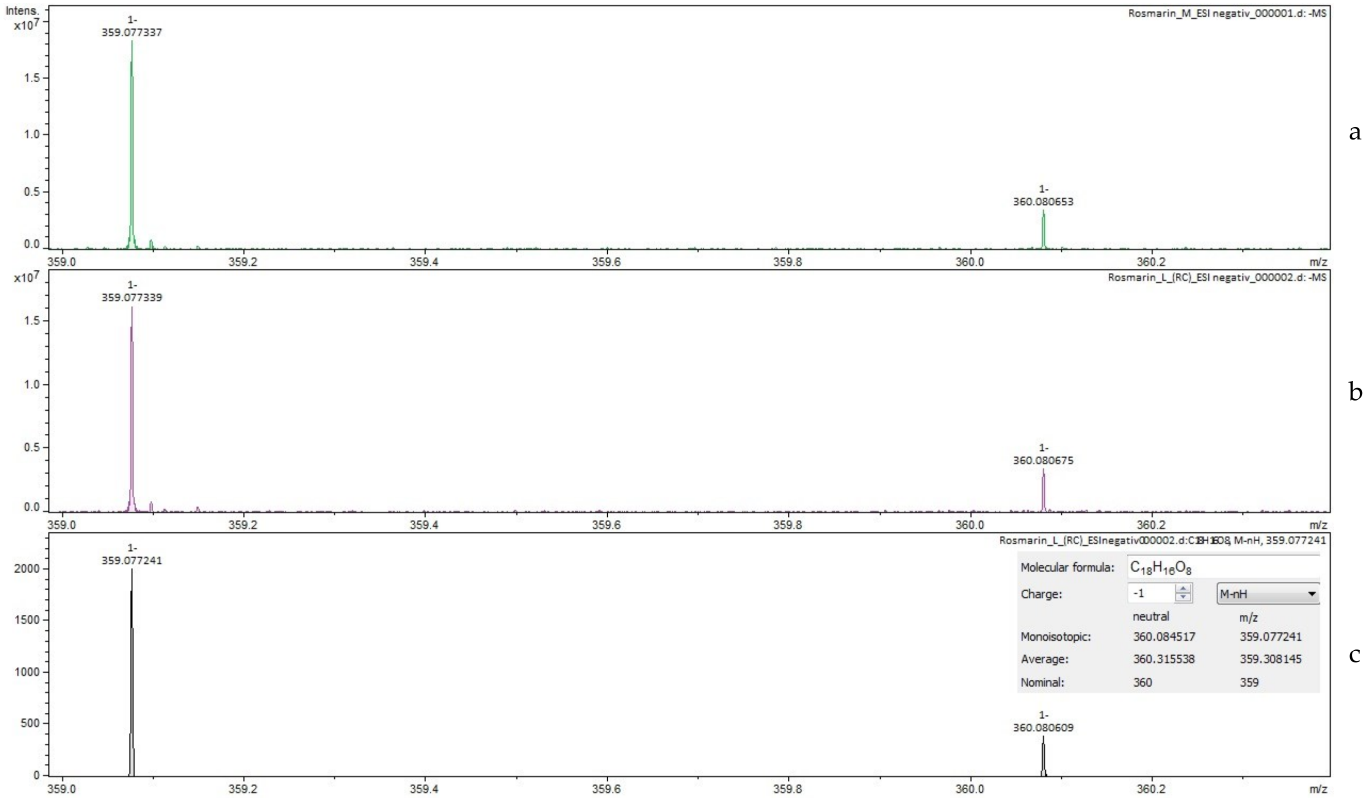

Figure S33. Rosmarinic acid ($C_{18}H_{16}O_8$) – m/z is 359.07, ESI-.

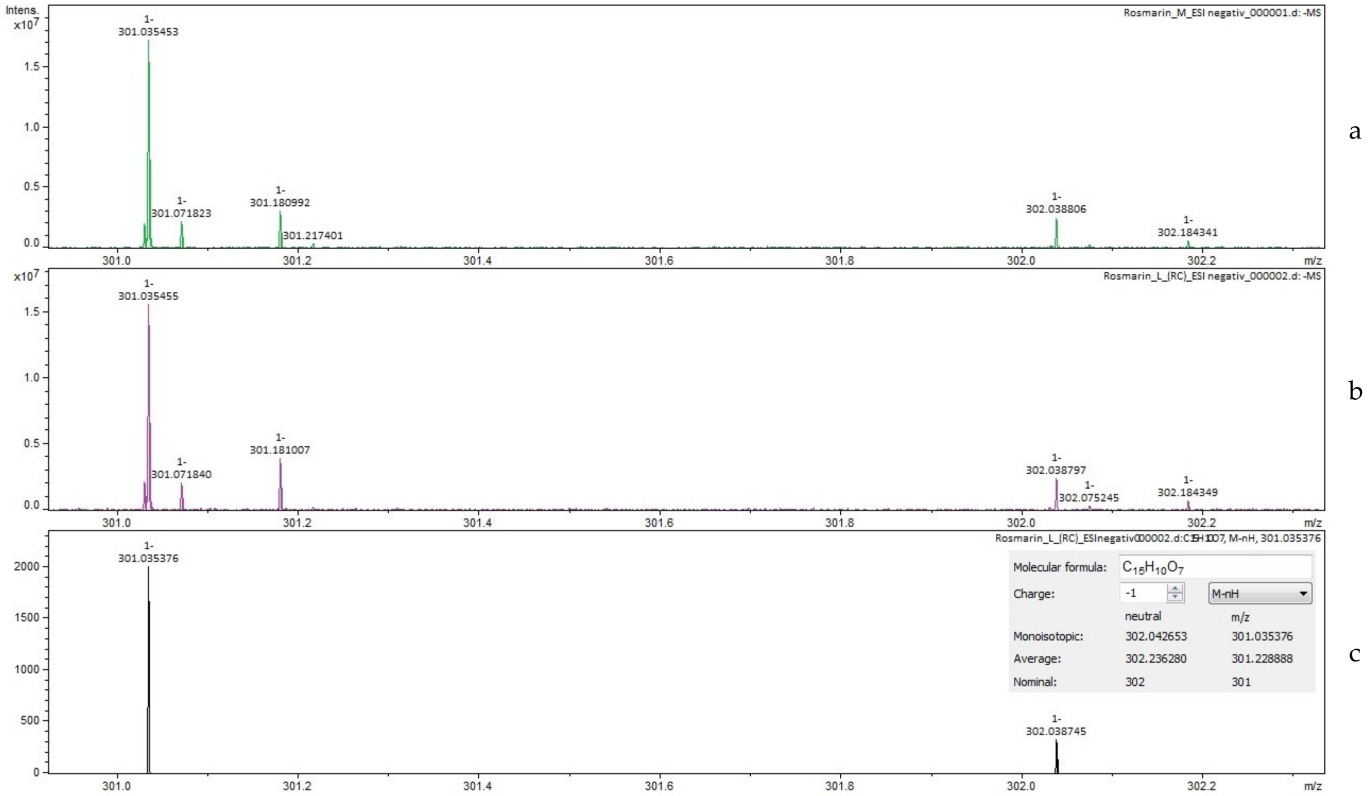

Figure S34. Quercetin ($C_{15}H_{10}O_7$) – m/z is 301.03, ESI-.

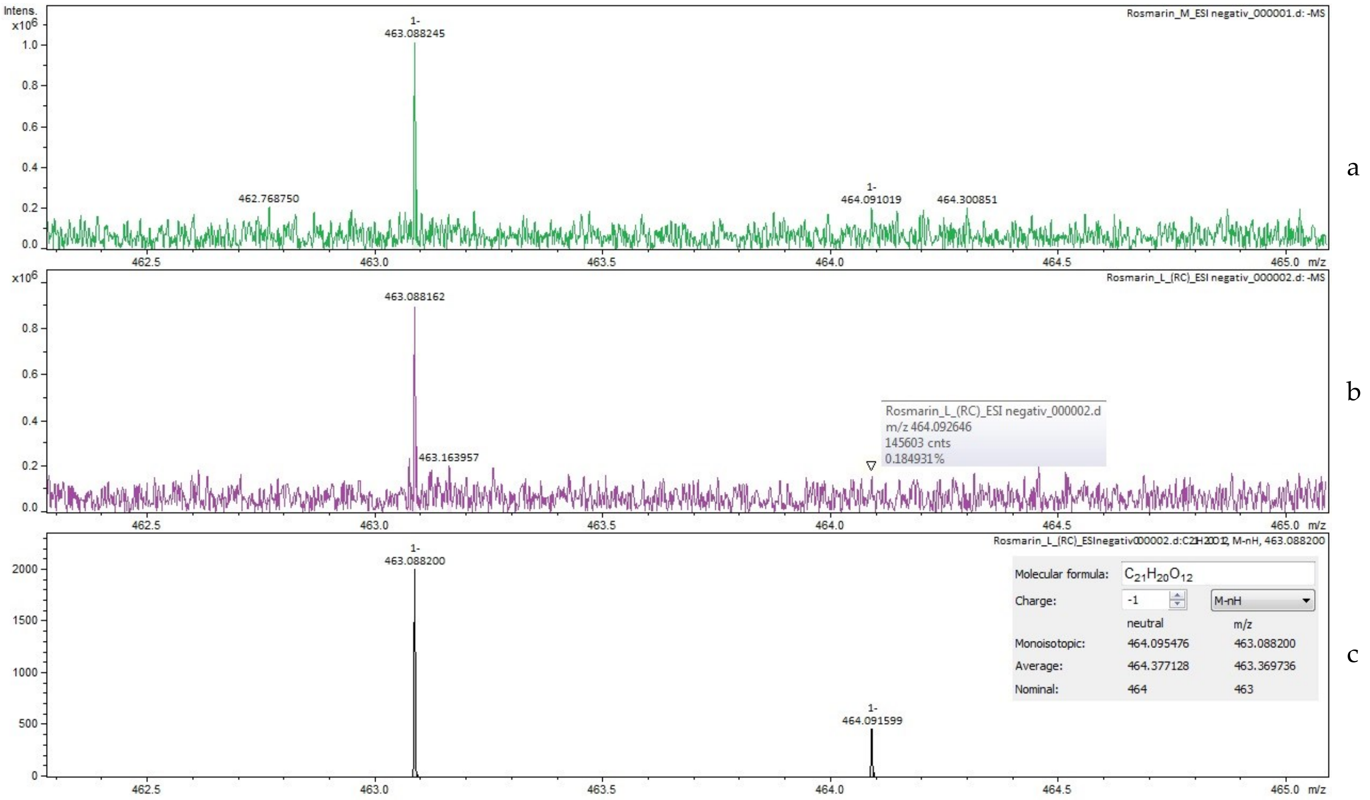

Figure S35. Isoquercitrin ($C_{21}H_{20}O_{12}$) – m/z is 463.08, ESI-.

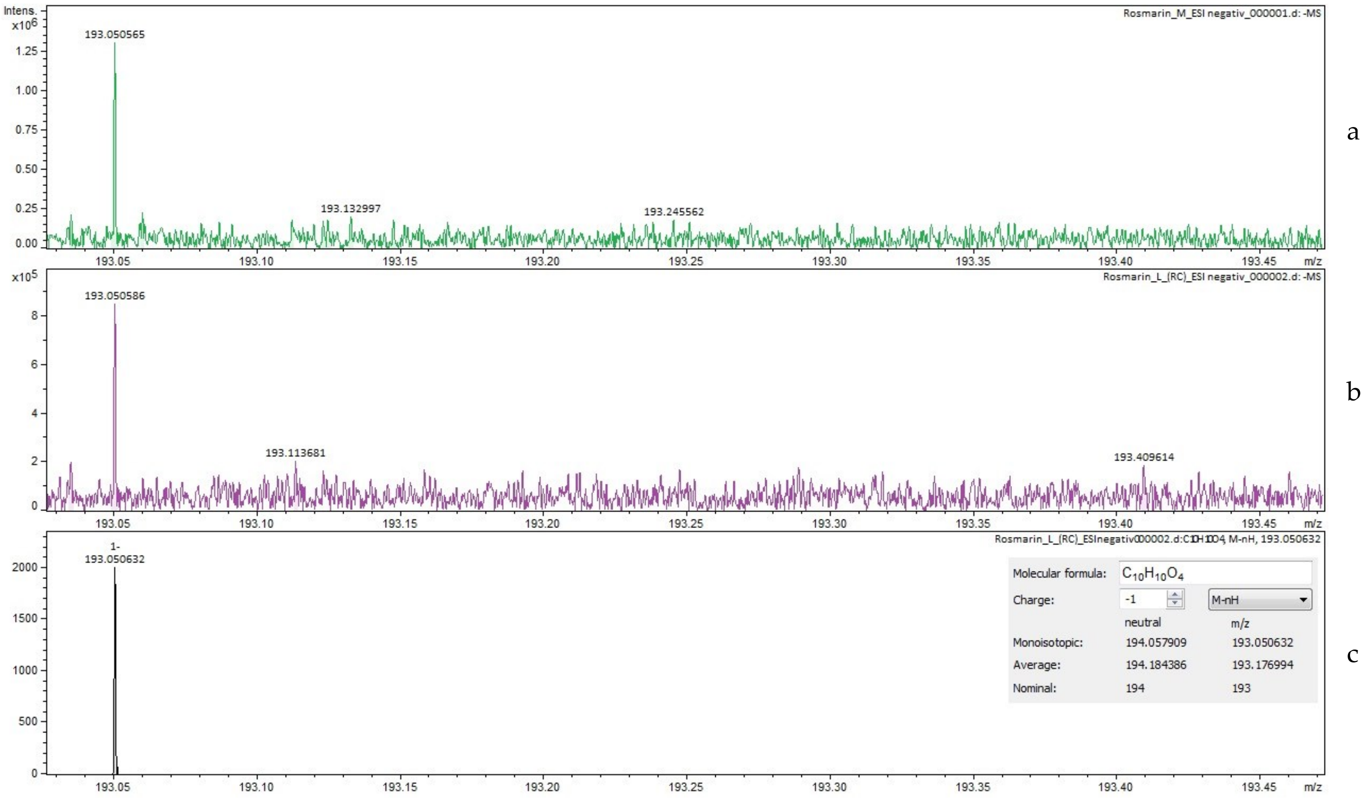

Figure S36. Ferulic acid ($C_{15}H_{10}O_6$) – m/z is 193.05, ESI-.

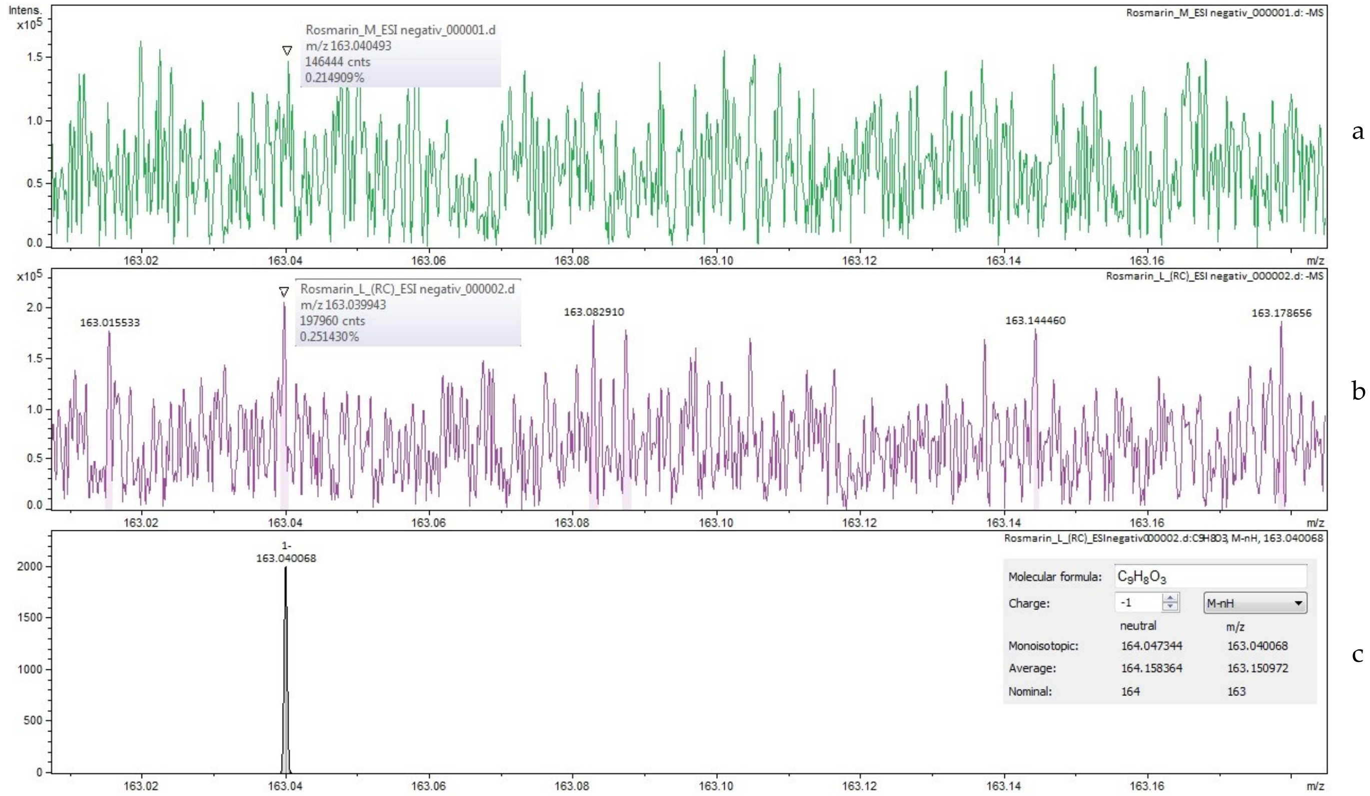

Figure S37. *p*-Coumaric acid ($C_9H_8O_3$) – m/z is 163.04, ESI-.

Thymus vulgaris L.

Blue color chromatogram – TEM (thyme extract from control crop) - a

Green color chromatogram – TEF (thyme extract from common crop) - b

Grey color chromatogram – theoretical peak obtained by the computer program - c

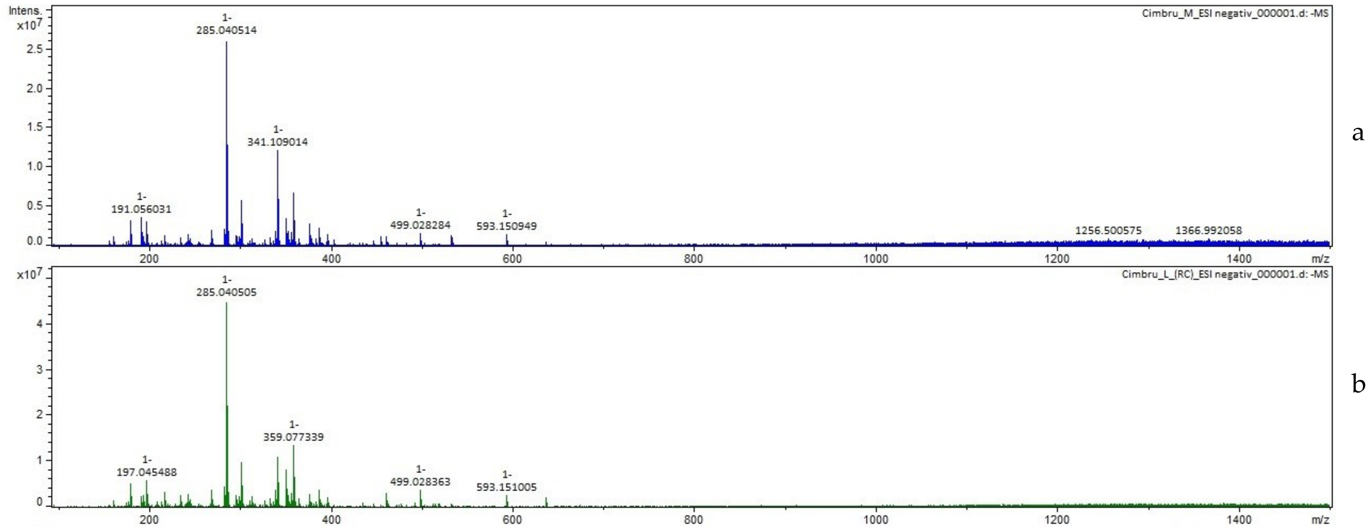

Figure S38. (a) and (b) – entire mass spectra of thyme dry extract obtained on negative ionization.

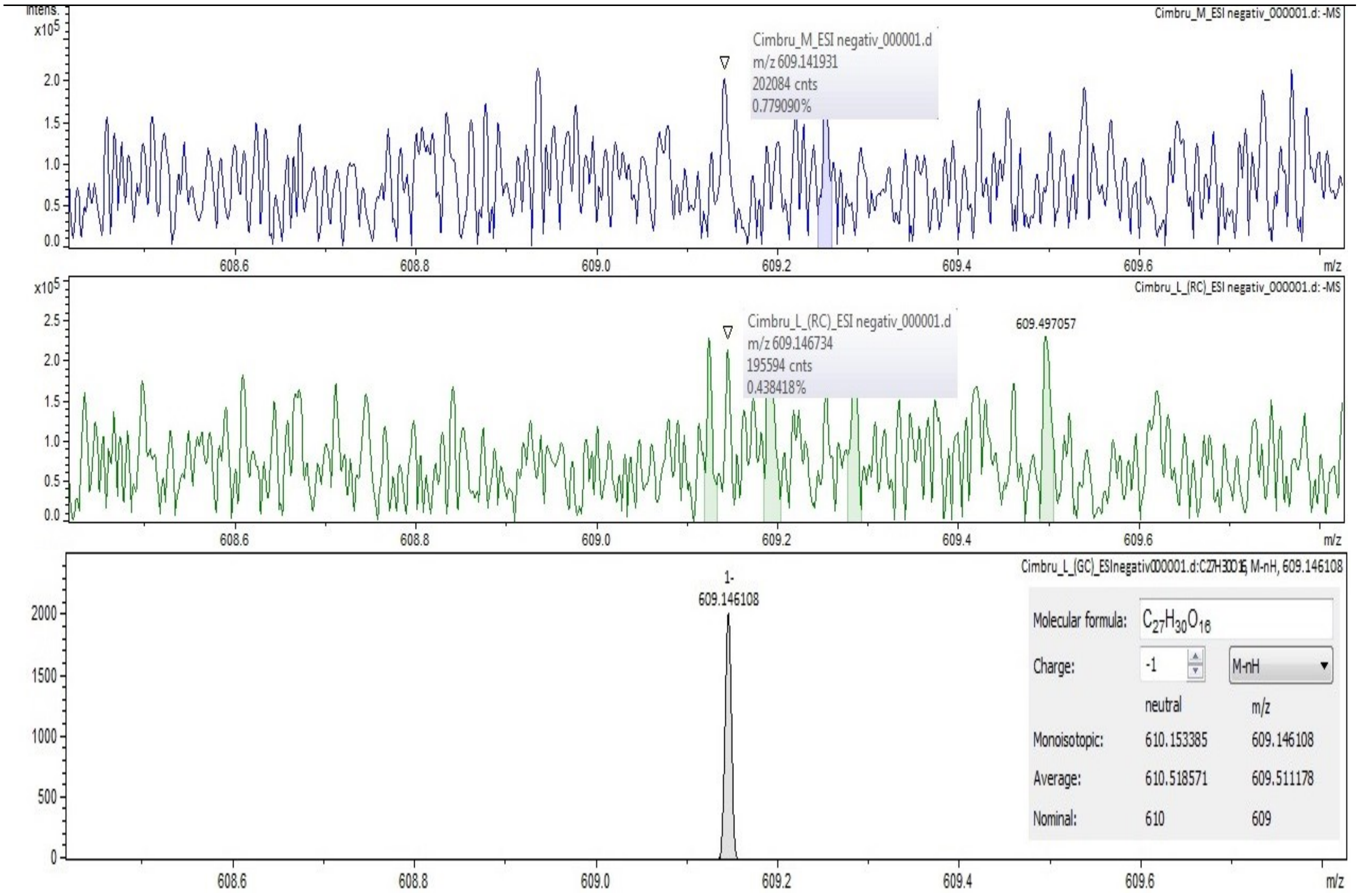

Figure S39. Rutin ($C_9H_8O_3$) – m/z is 609.15, ESI–.

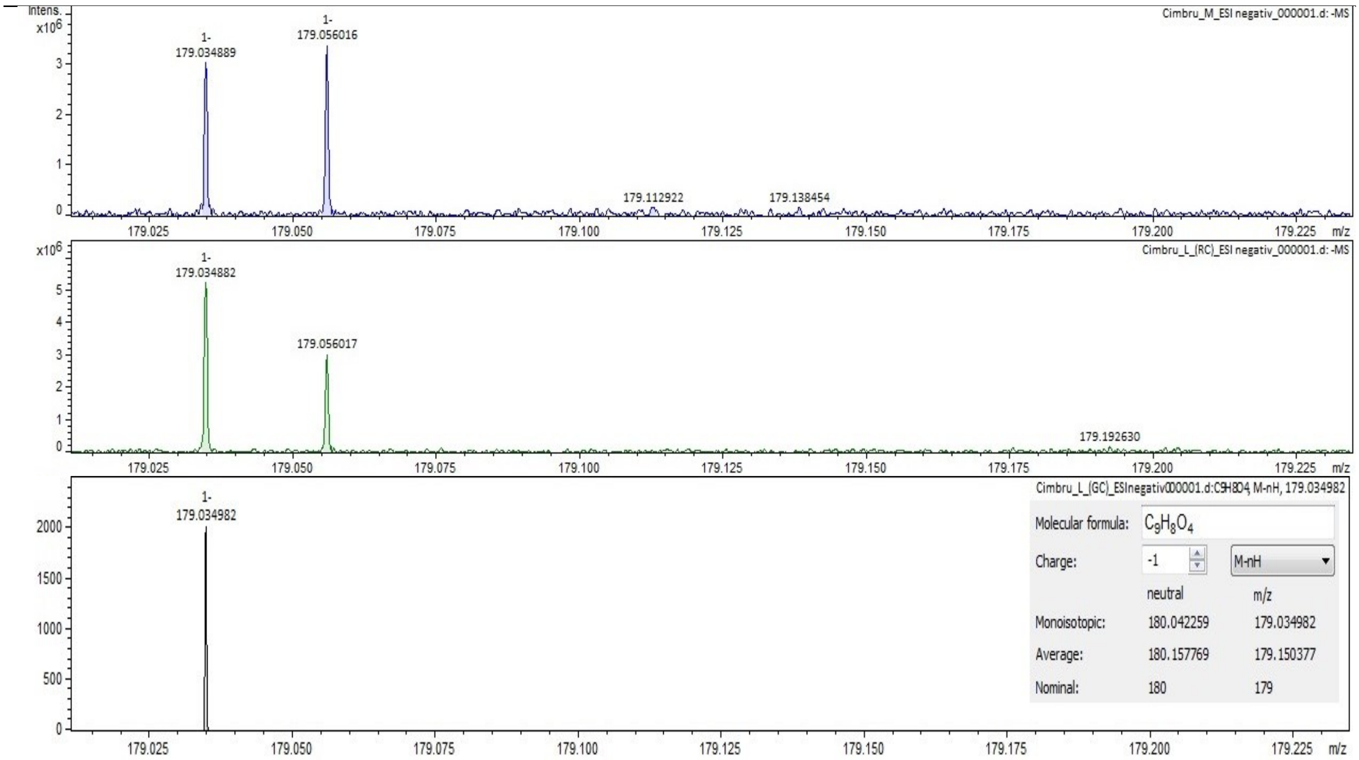

Figure S40. Caffeic acid ($C_9H_8O_4$) – entire chromatogram, m/z is 179.03, ESI-.

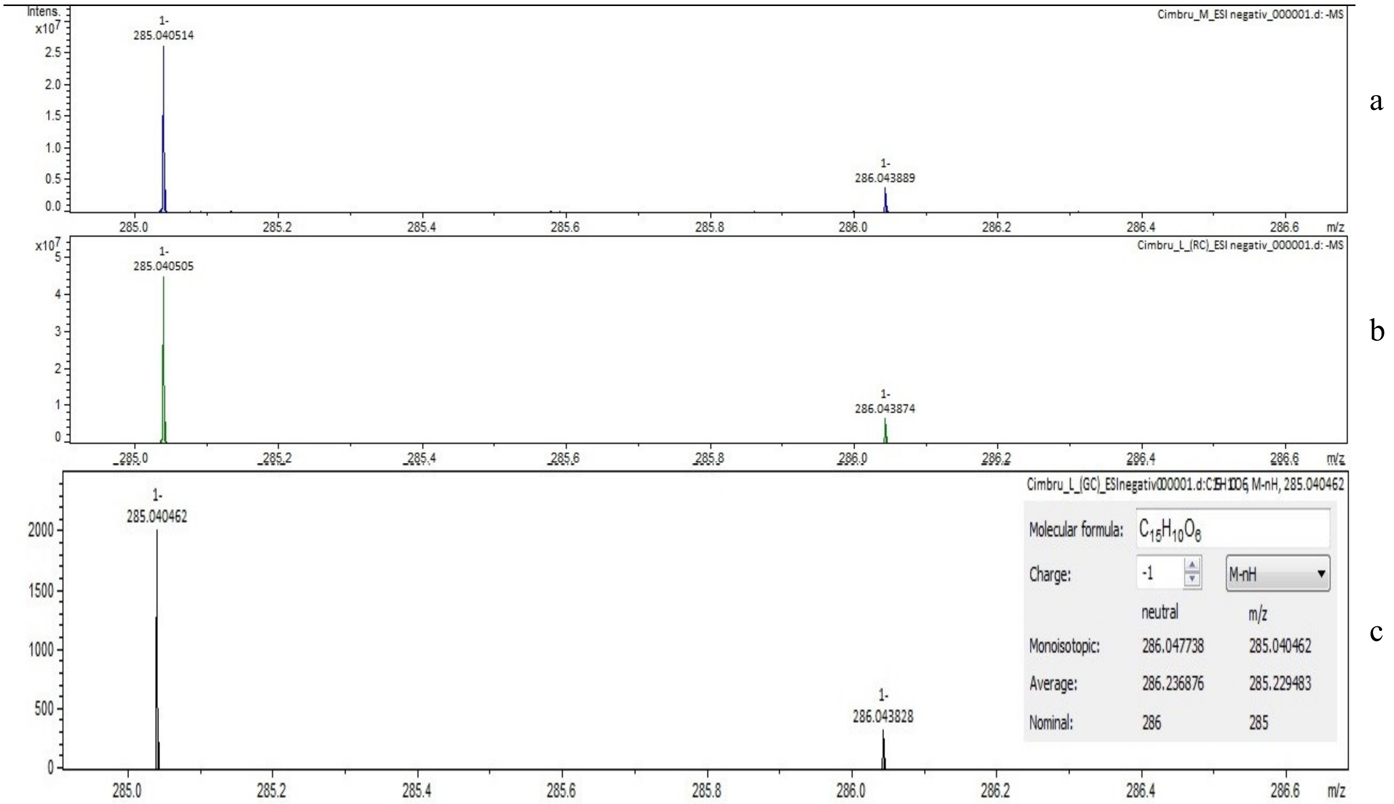

Figure S41. Luteolin + Kaempferol ($C_{15}H_{10}O_6$) – m/z is 285.04, ESI-.

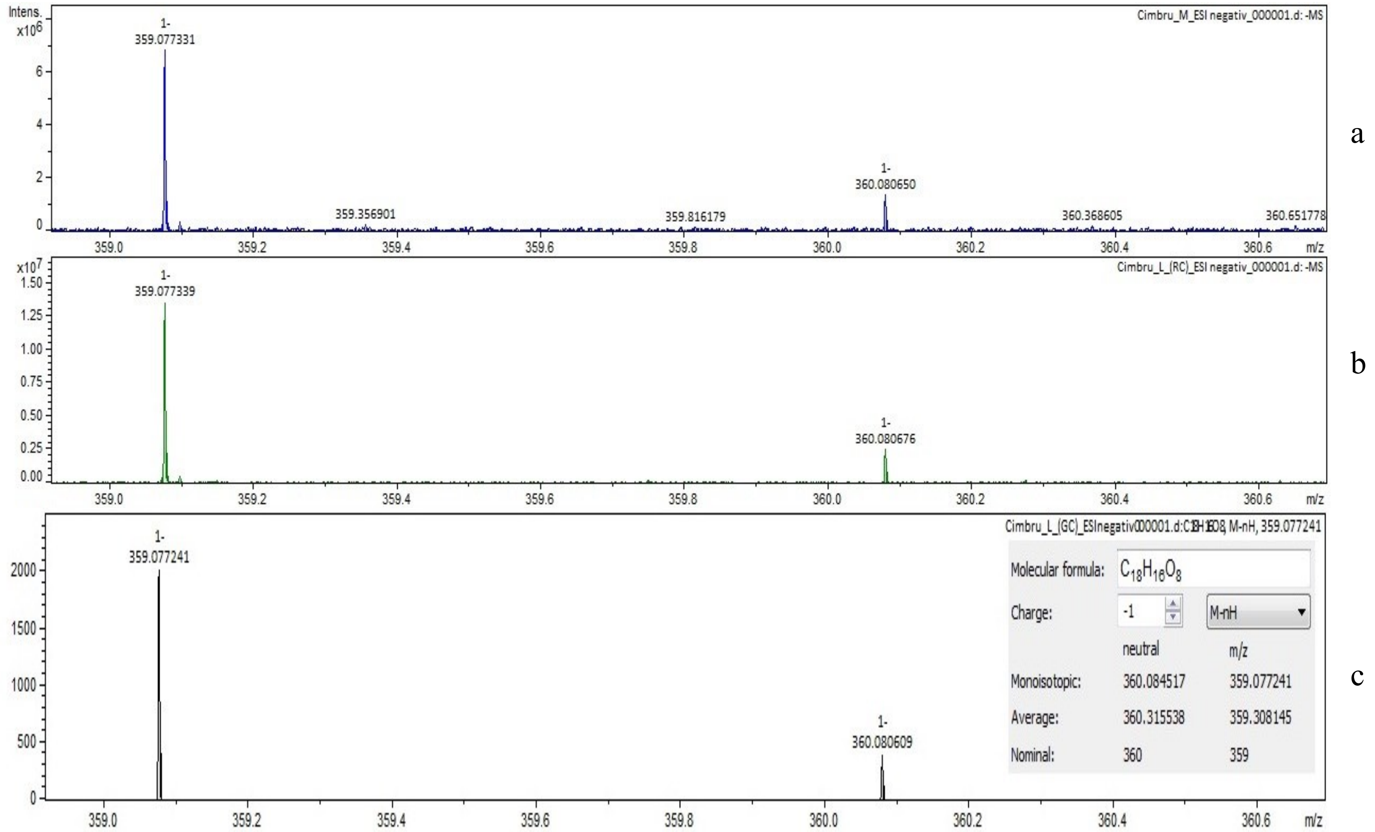

Figure S42. Rosmarinic acid ($C_{18}H_{16}O_8$) – m/z is 359.07, ESI-.

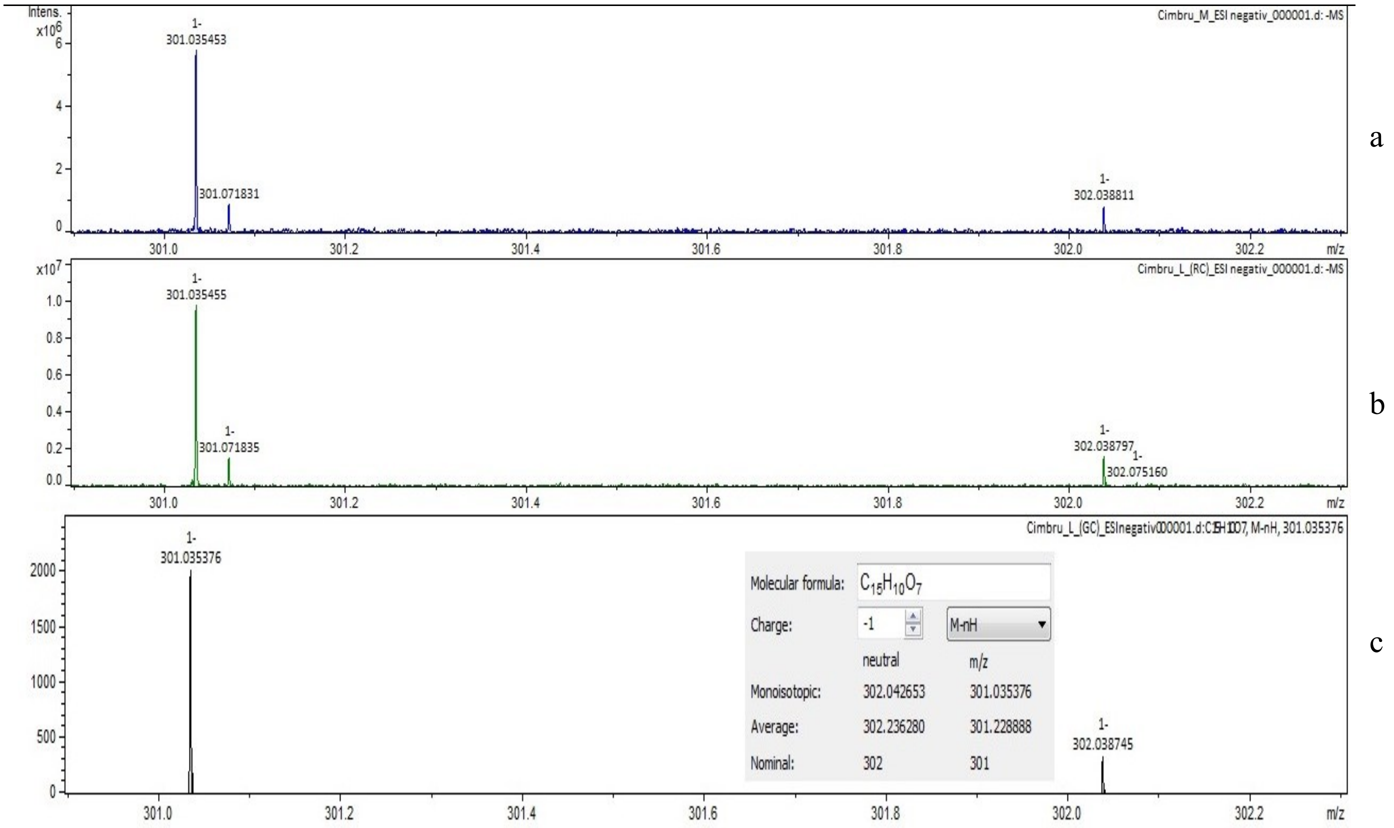

Figure S43. Quercetin ($C_{15}H_{10}O_7$) – m/z is 301.03, ESI-.

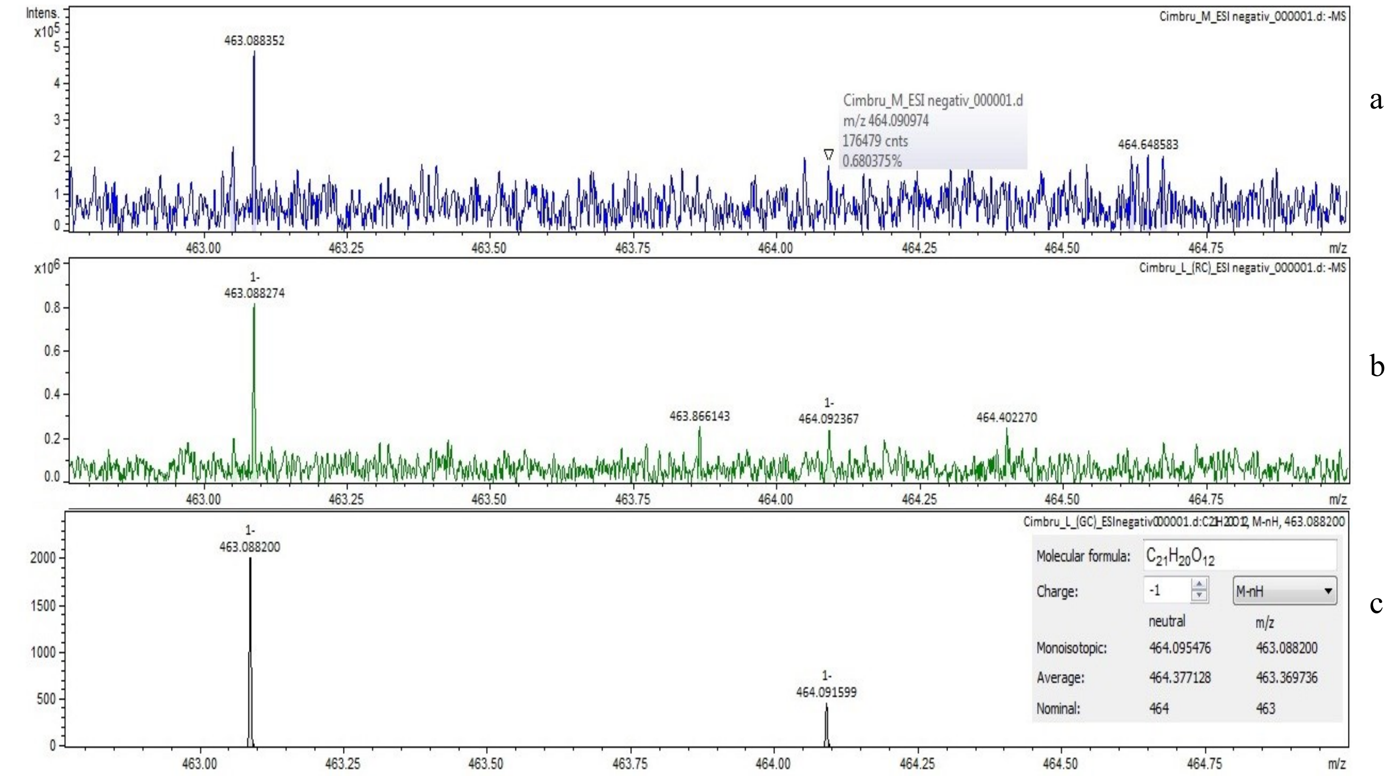

Figure S44. Isoquercitrin ($C_{21}H_{20}O_{12}$) – m/z is 463.08, ESI–.

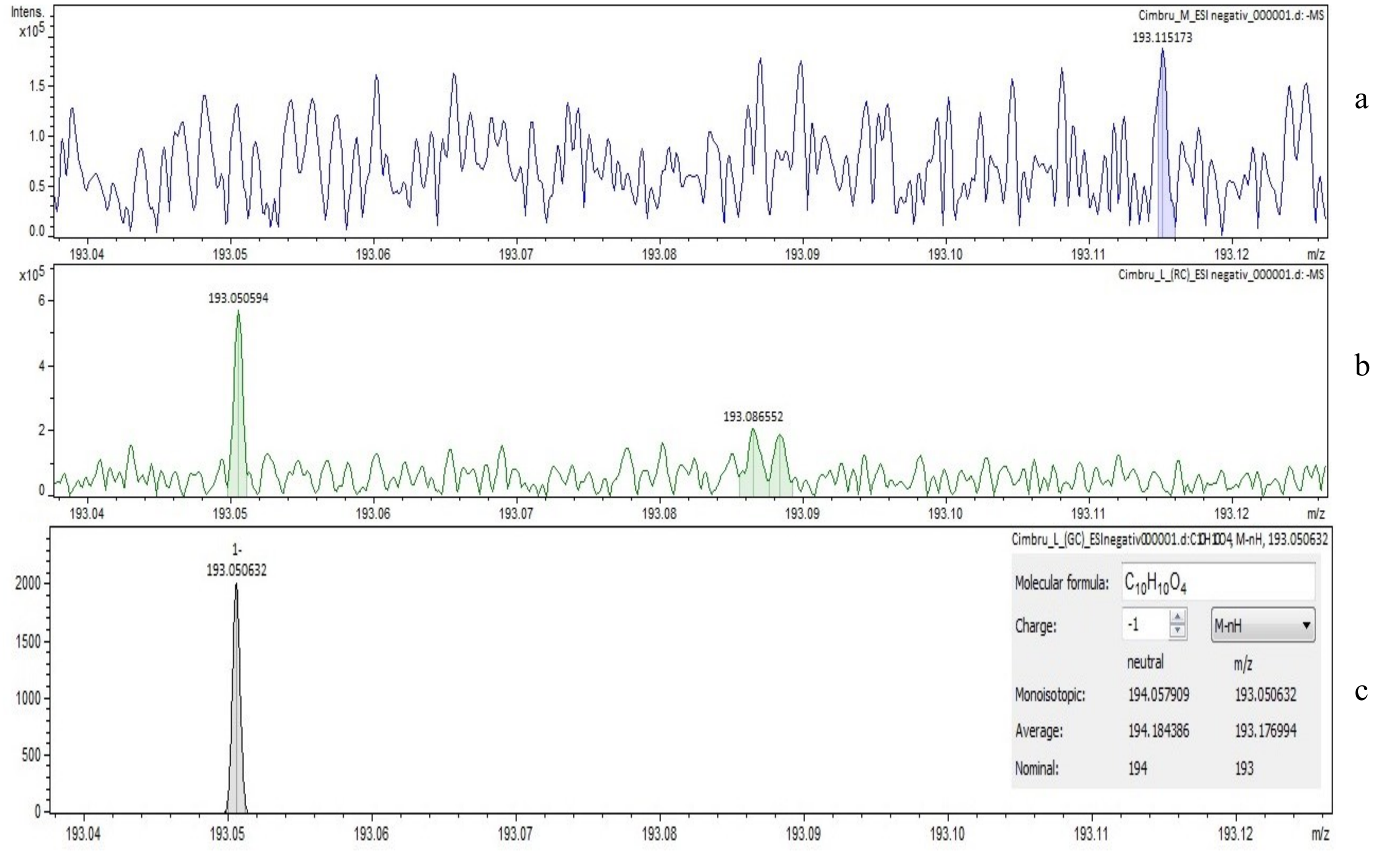

Figure S45. Ferulic acid ($C_{15}H_{10}O_6$) – m/z is 193.05, ESI-.

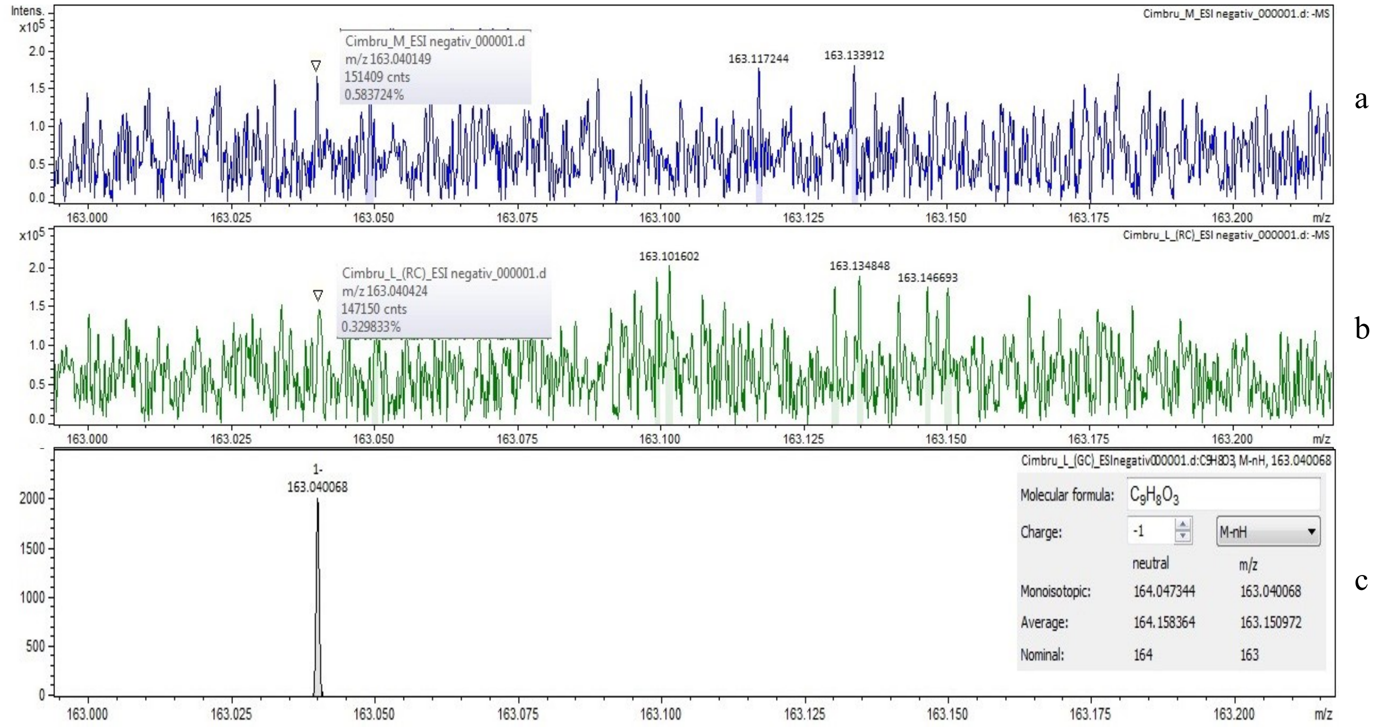

Figure S46. *p*-Coumaric acid ($C_9H_8O_3$) – m/z is 163.04, ESI-.

S2.3. Antioxidant activity

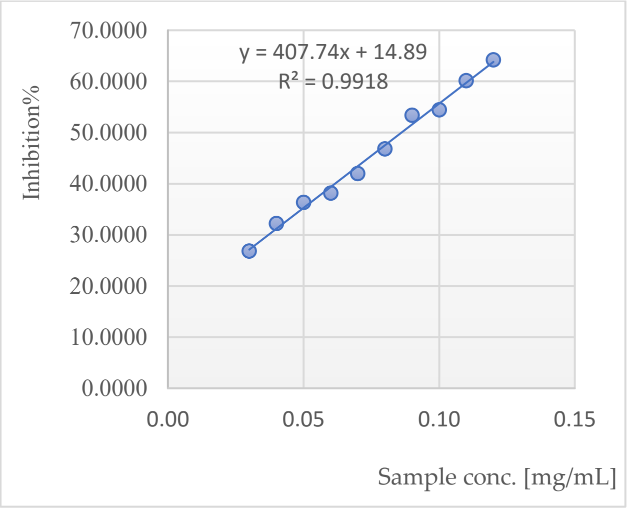

(a) REM

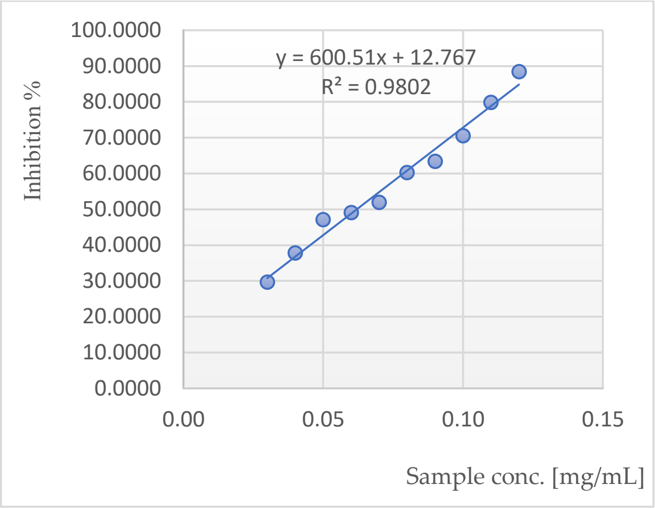

(b) REF

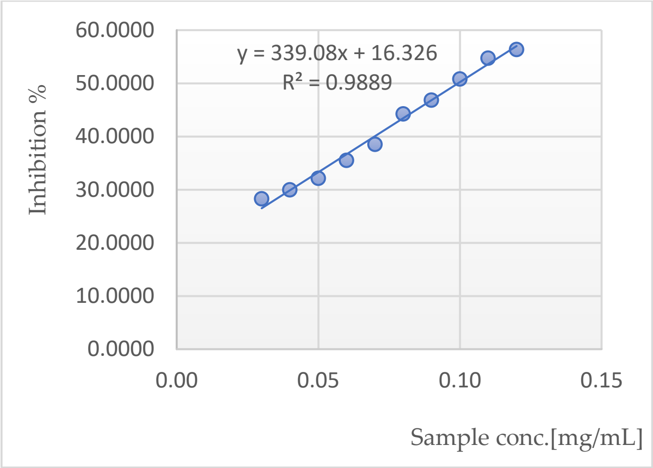

(c) TEM

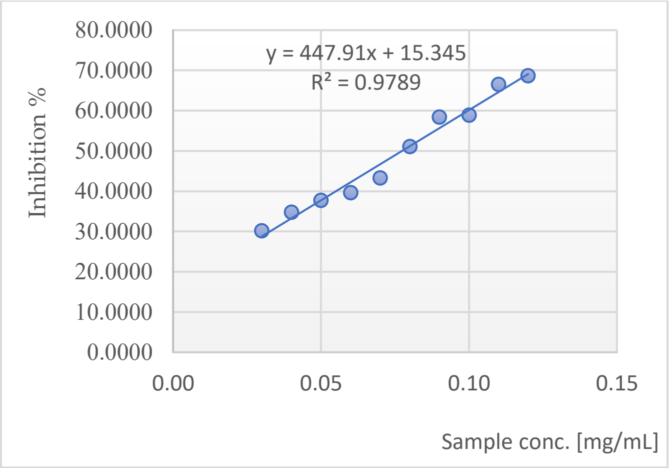

(d) TEF

A. DPPH method

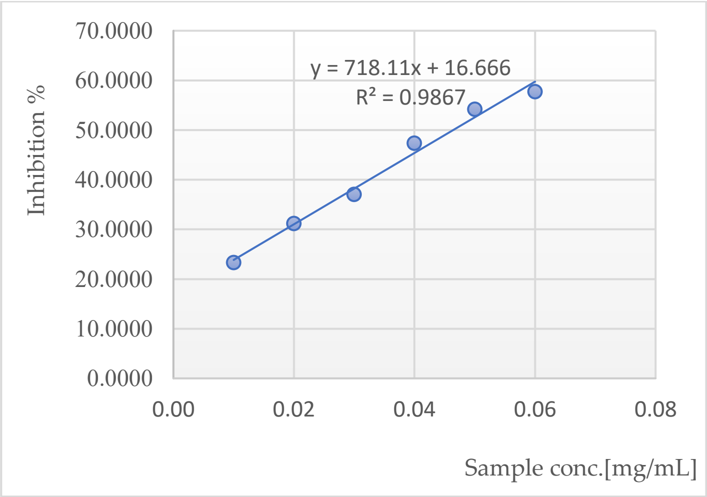

(a) REM

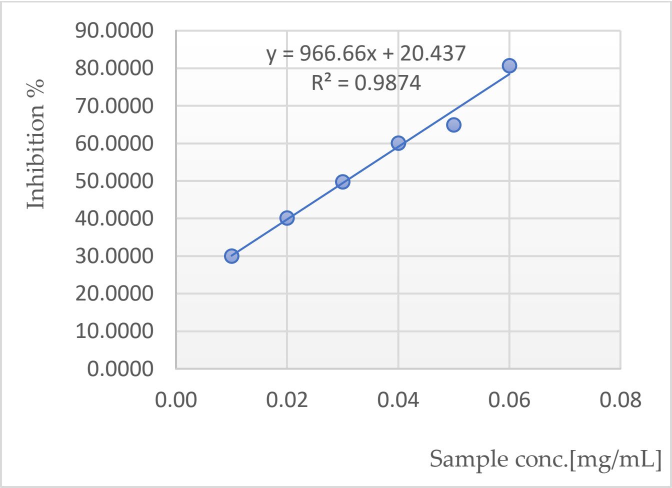

(b) REF

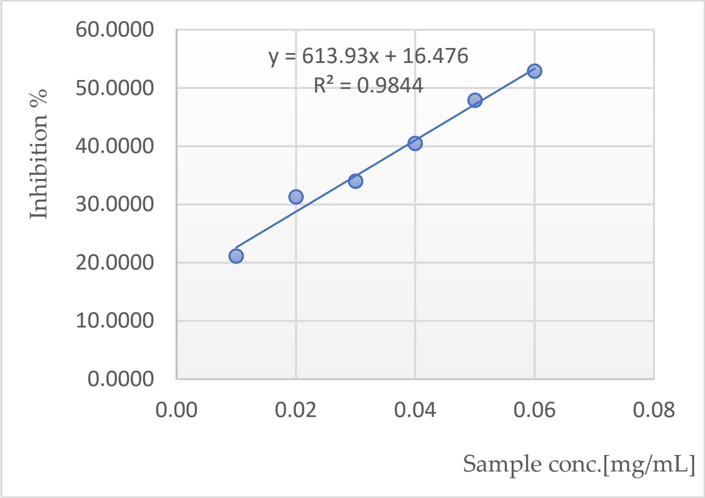

(c) TEM

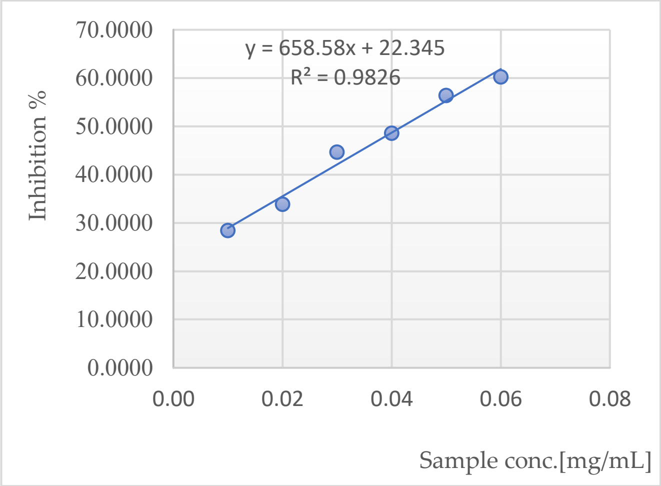

(d) TEF

B. ABTS method

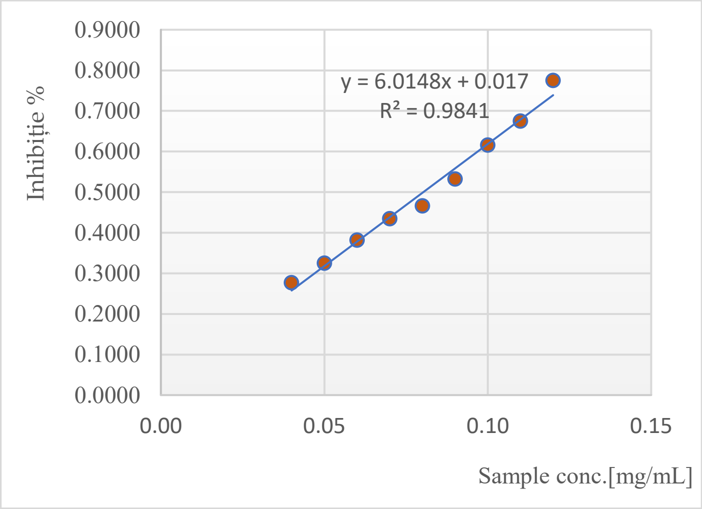

(a) REM

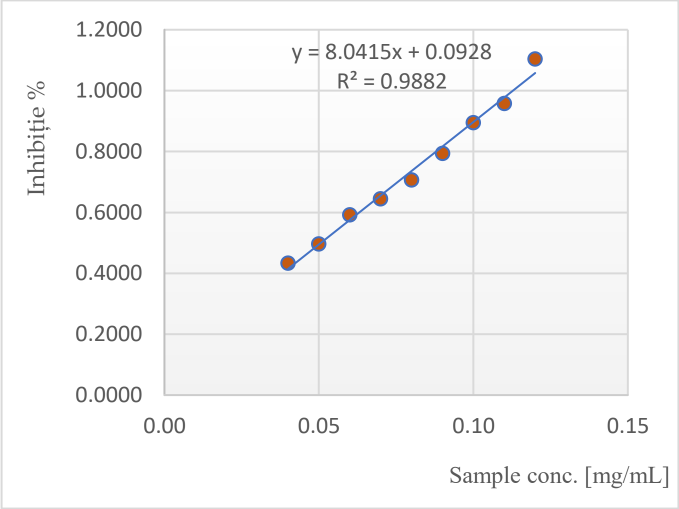

(b) REF

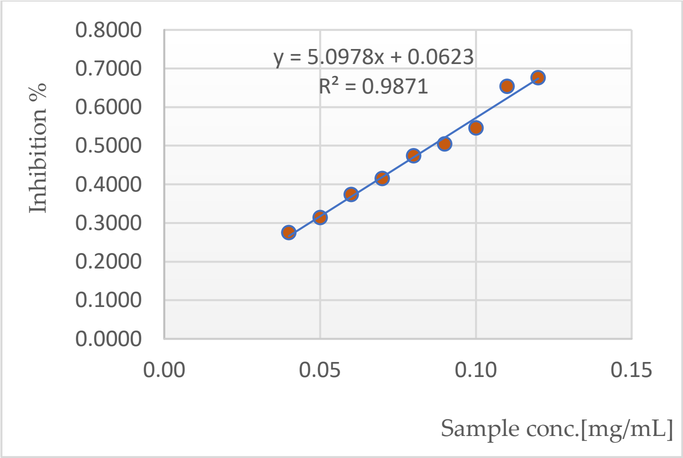

(c) TEM

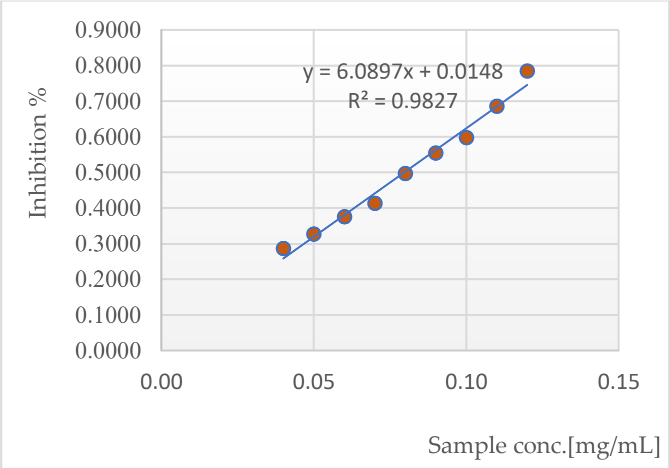

(d) TEF

C. FRAP method

Figure S47. The inhibitions depending on the concentrations for all dry extracts analyzed for (A) DPPH, (B)ABTS, and (C) FRAP method

Legend: REM – rosemary extract, control crop; REF rosemary extract, common crop; TEM – thyme extract, control crop; TEF - thyme extract, common crop

Table S3. Descriptive statistics and normality tests for the DPPH method.

Parameter	REM	TEM	REF	TEF	Δ_ROS_ DPPH	Δ_THYM _DPPH
Mean	45.4704	41.7573	57.8046	48.9385	12.3342	7.1812
Standard deviation (SD)	12.3957	10.3237	18.3644	13.7066	6.3346	3.6361
Sample size (N)	10	10	10	10	10	10
Std. error of mean (SEM)	3.9199	3.2646	5.8073	4.3344	2.0032	1.1498
Lower 95% conf. limit for mean	36.6031	34.3722	44.6674	39.1333	7.8027	4.5800
Upper 95% conf. limit for mean	54.3378	49.1424	70.9417	58.7436	16.8656	9.7823
Minimum	26.8293	28.2696	29.6860	30.2169	2.8567	1.9473
Maximum	64.2053	56.3985	88.4043	68.7172	24.1990	12.3187
Normality Test Shapiro-Wilk	0.967	0.932	0.983	0.932	0.961	0.908
Normality Test P value	0.861	0.470	0.978	0.471	0.792	0.267
Passed normality tests?	Yes ($p > 0.05$)	Yes ($p > 0.05$)	Yes ($p > 0.05$)	Yes ($p > 0.05$)	Yes ($p > 0.05$)	Yes ($p > 0.05$)

Legend : REM – rosemary extract, control crop; REF – rosemary extract, common crop; TEM – thyme extract, control crop; TEF – thyme extract, common crop; Δ_ROS_DPPH = DPPH_REF – DPPH_REM; Δ_THYM_DPPH = DPPH_TEF – DPPH_TEM.

Table S4. Descriptive statistics and normality tests for the ABTS method.

Group	REM	TEM	REF	TEF	Δ_ROS_ ABTS	Δ_THYM _ABTS
Mean	41.8003	37.9635	54.2699	45.3957	12.4696	7.4322
Standard deviation (SD)	13.5247	11.5762	18.1994	12.4294	5.6389	2.6795
Sample size (N)	6	6	6	6	6	6
Std. error of the mean (SEM)	5.5214	4.7260	7.4299	5.0743	2.3021	1.0939
Lower 95% conf. limit for mean	27.6070	25.8150	35.1708	32.3518	6.5519	4.6203
Upper 95% conf. limit for mean	55.9936	50.1120	73.3690	58.4396	18.3873	10.2441

Minimum	23.3391	21.1628	30.0163	28.4835	6.6772	2.5630
Maximum	57.7477	52.8924	80.6980	60.2762	22.9503	10.6735
Normality Test Shapiro-Wilk	0.951	0.980	0.989	0.952	0.863	0.879
Normality Test P value	0.747	0.953	0.988	0.755	0.198	0.263
Passed normality tests?	Yes<br>($p > 0.05$)	Yes<br>($p > 0.05$)	Yes<br>($p > 0.05$)	Yes<br>($p > 0.05$)	No<br>*(Highly skewed)*	No<br>*(Highly skewed)*

Legend: REM – rosemary extract, control crop; REF – rosemary extract, common crop; TEM – thyme extract, control crop; TEF – thyme extract, common crop; Δ_ROS_ABTS = ABTS_REF – ABTS_REM, Δ_THYM_ ABTS = ABTS _TEF – ABTS _TEM.

Table S5. Descriptive statistics and normality tests for the FRAP method.

Group	REM	TEM	REF	TEF	Δ_ROS_FRAP	Δ_THYM_FRAP (sqrt)
Mean	0.4982	0.4701	0.7361	0.5020	0.2379	0.1701
Standard deviation (SD)	0.1661	0.1405	0.2215	0.1682	0.0562	0.0899
Sample size (N)	9	9	9	9	9	9
Std. error of mean (SEM)	0.0554	0.0468	0.0738	0.0561	0.0187	0.0318
Lower 95% conf. limit for mean	0.3706	0.3621	0.5658	0.3727	0.1947	0.0949
Upper 95% conf. limit for mean	0.6259	0.5781	0.9064	0.6313	0.2811	0.2453
Minimum	0.2772	0.2749	0.4331	0.2859	0.1559	0.0374
Maximum	0.7753	0.6759	1.1042	0.7841	0.3289	0.3289
Normality Test Shapiro-Wilk	0.9696	0.9580	0.9760	0.9632	0.9708	0.9783
Normality Test P value	0.8911	0.7777	0.9403	0.8311	0.9017	0.9541
Passed normality tests?	Yes ($p > 0.05$)	Yes ($p > 0.05$)	Yes ($p > 0.05$)	Yes ($p > 0.05$)	Yes ($p > 0.05$)	Yes ($p > 0.05$)

Legend: REM – rosemary extract, control crop; REF – rosemary extract, common crop; TEM – thyme extract, control crop; TEF – thyme extract, common crop; Δ_ROS_FRAP = FRAP_REF – FRAP_REM; Δ_THYM_ FRAP = FRAP_TEF – FRAP_TEM.

Table S6. Independent Samples Test for FRAP method.

		Levene's Test		t-test for Equality of Means							
						Significance				90% CI of the Difference	
		F	Sig.	t	*df*	One-Sided *p*	Two-Sided *p*	Mean Diff.	Std. Error Diff.	Lower Bound	Upper Bound
Score	Equal variances assumed	1.307	0.271	1.890	15	0.039	0.078*	0.0678	0.0359	0.0049	0.1308

*. The significance level is 0.10.
